# Triazolopeptides Inhibiting the Interaction between Neuropilin-1 and Vascular Endothelial Growth Factor-165

**DOI:** 10.3390/molecules24091756

**Published:** 2019-05-06

**Authors:** Bartlomiej Fedorczyk, Piotr F. J. Lipiński, Anna K. Puszko, Dagmara Tymecka, Beata Wilenska, Wioleta Dudka, Gerard Y. Perret, Rafal Wieczorek, Aleksandra Misicka

**Affiliations:** 1 Faculty of Chemistry, University of Warsaw, Pasteura 1, 02-093 Warsaw, Poland; apuszko@chem.uw.edu.pl (A.K.P.); dulok@chem.uw.edu.pl (D.T.); bwilenska@chem.uw.edu.pl (B.W.); wieczorek@chem.uw.edu.pl (R.W.); 2 Department of Neuropeptides, Mossakowski Medical Research Centre, Polish Academy of Sciences, Pawinskiego 5, 02-106 Warsaw, Poland; plipinski@imdik.pan.pl; 3 Laboratory of Cytometry, Nencki Institute of Experimental Biology, Polish Academy of Sciences, Pasteura 3, 02-093 Warsaw, Poland; wioletadudka@gmail.com; 4 Université Paris 13, Sorbonne Paris Cité, INSERM U1125, 74 rue Marcel Cachin, 93017 Bobigny, France; perretgerard@sfr.fr

**Keywords:** peptidomimetics, VEGF_165_, neuropilin-1, molecular dynamics, structure–activity relationship

## Abstract

Inhibiting the interaction of neuropilin-1 (NRP-1) with vascular endothelial growth factor (VEGF) has become an interesting mechanism for potential anticancer therapies. In our previous works, we have obtained several submicromolar inhibitors of this interaction, including branched pentapeptides of general structure Lys(Har)-Xxx-Xxx-Arg. With the intent to improve the proteolytic stability of our inhibitors, we turned our attention to 1,4-disubstituted 1,2,3-triazoles as peptide bond isosteres. In the present contribution, we report the synthesis of 23 novel triazolopeptides along with their inhibitory activity. The compounds were synthesized using typical peptide chemistry methods, but with a conversion of amine into azide completely on solid support. The inhibitory activity of the synthesized derivatives spans from 9.2% to 58.1% at 10 μM concentration (the best compound Lys(Har)-GlyΨ[Trl]GlyΨ[Trl]Arg, **3**, IC_50_ = 8.39 μM). Synthesized peptidotriazoles were tested for stability in human plasma and showed remarkable resistance toward proteolysis, with half-life times far exceeding 48 h. In vitro cell survival test resulted in no significant impact on bone marrow derived murine cells 32D viability. By means of molecular dynamics, we were able to propose a binding mode for compound **3** and discuss the observed structure–activity relationships.

## 1. Introduction

Neuropilin-1 (NRP-1), vascular endothelial growth factor receptor type-2 (VEGFR-2) and vascular endothelial growth factor 165 (VEGF_165_) form a complex (VEGFR-2/NRP-1/VEGF_165_), which modulates the process of angiogenesis [1]. The interaction of VEGF_165_ with NRP-1 significantly increases VEGF_165_/VEGFR-2 binding strength [2,3] and, as a result, it increases the proangiogenic signal exerted by this complex. NRP-1 is not only expressed by endothelial cells of blood vessels, but also by several immune system cell types such as plasmacytoid dendritic cells [4] or regulatory T cells (Tregs) [5]. The protein is thus involved in the regulation of immune response [6].

Aside from the part that NRP-1 plays in physiological processes, it plays many roles in cancer. The protein is expressed by numerous types of cancer cells [7] and its overexpression was found to be related to tumor malignancy and poor prognosis [8,9]. It is believed that this phenomenon is caused by an autocrine pathway through the VEGF_165_/NRP-1 axis, which favors cancer cells’ migration, proliferation, and growth [10], and also increases their survivability [11]. Additionally, Tregs tend to express large amounts of NRP-1 on their surface. Thanks to this, they migrate toward the source of the chemoattractant, tumor originated VEGF_165_, and are recruited into the tumor microenvironment, causing cancer-associated immunosuppression [12].

The involvement of VEGF_165_/NRP-1 interaction in cancer pathology makes its inhibition an interesting approach for finding novel anticancer therapies [13,14,15]. Several groups (including ours) proposed a number of different VEGF_165_/NRP-1 inhibitors. These include small molecules [16,17,18,19,20,21], VEGF_165_ derived peptides [22,23], or cyclic peptides [24,25,26]. Another set of the inhibitors proposed so far consists of the peptide A7R (ATWLPPR) [27] and derivatives inspired by this sequence [28,29,30]. A7R exhibits a relatively good inhibition in in vitro tests with an IC_50_ of 5.86 μM [24]. Further, the compound has been proven to be active in vivo in nude mice xenografted with drug-resistant MDA-MB-231 human breast cancer cells [31].

In our previous works, we aimed at modifying the A7R sequence in order to improve the activity and stability of the inhibitors. In their course, we have obtained branched pentapeptides of the general structure Lys(Har)-Xaa-Xaa-Arg [29]. The results of their in vitro testing and that of the other derivatives of ours [31,32,33] led us to the conclusion that N-terminus branching is beneficial for inhibitory activity of the VEGF_165_/NRP-1 interaction.

Regarding stability, we tested the degradation pathway under proteolytic conditions of the strongest inhibitors Lys(Har)-Dab/Dap-Pro-Arg (IC_50_ = 0.2 μM) and Lys(Har)-Pro-Ala/Dab-Arg (IC_50_ = 0.2/0.3 μM, respectively) [29]. There, we observed that first enzymatic cleavage occurred in the middle part of the molecule, leading to splitting of the molecule into halves possessing no inhibitory activity. For this reason, we decided to prepare derivatives with a non-classical peptide bond bioisostere at the sites prone to proteolysis (in the middle part of the molecule). Our attention was turned to the 1,4-disubstituted 1,2,3-triazole ring. Incorporation of triazoles into a peptide chain is a popular approach for the preparation of mimetics [33,34] owing to its efficient and easy preparation step through 1,3-dipolar cycloaddition catalyzed with copper ions, a click reaction [35,36].

In the present contribution, we report the synthesis and in vitro inhibitory activity of 23 triazolopeptide analogues. Further, the rationalization for the observed structure–activity trends is provided based on molecular dynamics simulations. For the best hits, we have also investigated their proteolytic resistance in human plasma as well as viability against normal cell lines.

## 2. Results and Discussion

**Figure 1:**
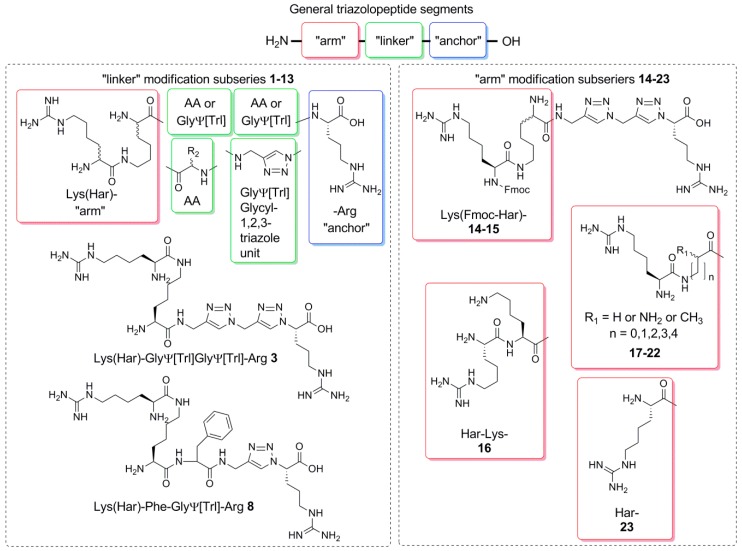
The general description of used notation and two exemplary structures **3** and **8**. Between the N-terminal Har and C-terminal Arg, there are two or three amino acids/glycyl-1,2,3-triazole units in various configurations, where AA stands for canonical amino acid residue and GlyΨ[Trl] is a glycyl-1,2,3-triazole unit mimicking glycine (triazole ring substitution instead of peptide bond). All analogues were divided into two subseries with modification in the “linker” site of the molecule and at the “arm” site.

**Figure 2:**
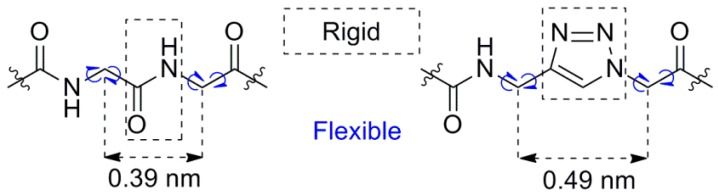
General comparison of trans peptide bond and 1,4-disubstituted 1,2,3-triazole ring as trans peptide bond non-classical bioisostere, adapted from the work of [37]. Every such triazole ring modification leads to backbone elongation and different conformational latitude.

Previous works have shown that strong inhibition of the NRP-1/VEGF_165_ interaction is obtained when the structure contains a C-terminal arginine (“anchor”) and a certain positively charged residue at the N-terminus (“arm”), spaced by two residues in the middle part (“linker”) [28,29,30,32]. Modifying this “linker” part by introducing triazole rings (in a glycyl-1,2,3-triazole unit—Figure 1) as peptide bond mimetics changes the flexibility of the backbone. Furthermore, it increases the geometrical distance between the “arm” and the “anchor” (Figure 2). The replacement of a peptide bond for a triazole ring elongates the distance between neighboring Cα atoms by about 10 nm.

Both changes (flexibility and length) can be expected to influence the activity. Therefore, we set out for rational investigation of structure–activity relationships within the triazolopeptide inhibitors. The research focused on two lines of variation. First, we modified the “linker” (“linker” subseries) with the aim to establish the best configuration of triazole rings in the backbone. Then, the “arm” was subjected to variation (“arm” subseries). On the basis of best hits from “linker” subseries, we wanted to see if changes related to backbone elongation can be compensated by modification of the “arm” (Figure 1). The scheme of the modifications is given in Figure 1. The full list of compounds with their sequences is presented in Table 1.

### 2.1. Synthesis of Triazolopeptides on Solid Support

**Figure 3:**
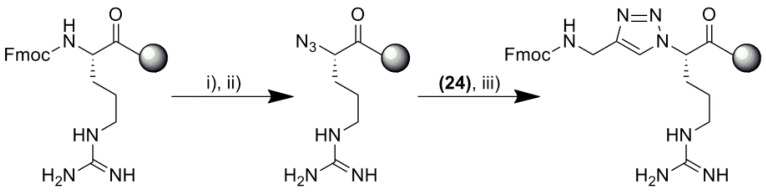
The general scheme of triazole rings synthesis on solid support. Reagents and conditions: (i) 20% piperidine in DMF; (ii) trifluoromethanesulfonyl azide in DCM, MeOH and K_2_CO_3_, CuSO_4_ in H_2_O (2:1:1 *v*/*v*/*v*), and Fmoc-propargylamine **24**; (iii) THF and CuSO_4_, sodium ascorbate in H_2_O (2:1 *v*/*v*).

The designed triazolopeptides were prepared on solid support, using standard polystyrene Wang resin preloaded with Fmoc-Arg(Pbf). Fmoc group deprotection (20% piperidine in DMF) and amino acid coupling (DIC/OxymaPure/Fmoc-AA) steps were proceeded in the standard manner. What is less usual, we incorporated 1,4-disubstituted 1,2,3-triazole moiety in the peptide backbone, completely on solid support. For this purpose, a modification of a standard solid phase peptide synthesis was carried out. The general synthesis scheme of triazole rings is shown in Figure 3.

#### 2.1.1. Conversion of Primary Amine Group into Azide on Solid Support

First, we transformed primary amine group of peptidyl-resin into N-terminal azide. To achieve this, we used the Wong diazotransfer reaction [38], carried out according to protocol from the literature [39], but adjusted to proceed directly on the solid support. Peptidyl-resin was treated with mixture of trifluoromethanesulfonyl azide (c.a. 2 eq.) in DCM, MeOH, and H_2_O with dissolved potassium carbonate (1 eq.) and copper(II) sulfate pentahydrate (0.067 eq.). Solvents volume ratio was 2:1:1 (DCM/MeOH/H_2_O). The reaction was left overnight with constant shaking, not stirring. Completion of the reaction was monitored by Kaiser test (negative result after one repetition).

#### 2.1.2. Click Reaction on Solid Support

The peptidyl-resin with N-terminal azide group was treated with a mixture of Fmoc-propargylamine (**24**) (2 eq.) dissolved in THF and copper(II) sulfate pentahydrate (0.2 eq) reduced by sodium ascorbate (0.67 eq.), both dissolved in H_2_O. The solvent volume ratio was 2:1 (THF:H_2_O). The reaction was left for at least 10 h under an argon atmosphere. Completion of the reaction was determined by modified Kaiser test with azide reduction beforehand according to the procedure described in the literature [40] (negative result after one repetition).

#### 2.1.3. Homoarginine (Har) Synthesis on Solid Support

Har residue was obtained through guanylation reaction of ε-amino group of Boc-Lys(Fmoc) after Fmoc deprotection. For this purpose, we used 3,5-dimethylpyrazole-1-carboxamidine nitrate (10 eq.) and DIPEA (to pH c.a. 10–11), following a procedure described in the literature [41].

#### 2.1.4. Final Cleavage from Solid Support and Triazolopeptides Isolation

The complete sequence was cleaved from solid support with the mixture of TFA/TIS/H_2_O with a volume ratio of 95:2.5:2.5. The reaction was carried out for at least three hours. Then, the resin was filtrated and washed with neat TFA three times. Solvents were connected and evaporated. To the residual oil, a cold diethyl ether was added in c.a. 15 times volume to precipitate triazolopeptides. Further, the crude product was filtered off, analyzed, purified on RP-HPLC with C-12 resin, and characterized with mass spectrometry. Analytical data for the compounds **1**–**23** are given in Table 1.

### 2.2. Inhibition of NRP-1/VEGF-A

The inhibitory activity of obtained triazolopeptides **1**–**23** toward binding of VEGF_165_ to NRP-1 was measured with the enzyme-linked immunosorbent assay (ELISA). This protocol was described earlier in the literature [42] and was broadly used by our group [24,26,28,29,30,32]. VEGF_165_ was used in biotinylated form (bt-VEGF_165_).

The inhibitory activity listed in Table 2 (at 10 μM, denoted further as *inh*) of the obtained derivatives spans from 9.2% (**12**) to 58.1% (**3**). None of the compounds described here are better than reference A7R (IC_50_ = 5.86) [24] or than the best of the branched pentapeptides that we have reported before (IC_50_ ~ 0.2 μM) [29]. Thus, at least within the structural space explored herein, triazole mimetics of the peptide bond are not very favourable for the activity on their own.

The obtained derivatives were grouped into two lines of structural variation (“linker” and “arm” subseries). The first one was aimed at deciphering the optimal triazole-containing spacer for H-Lys(Har)-linker-Arg-OH structure (“linker” series). Among the studied bridges, -GlyΨ[Trl]Arg-; -Pro-GlyΨ[Trl]-; -Phe-GlyΨ[Trl]-; -GlyΨ[Trl]Ala-; -GlyΨ[Trl]Gly-; -GlyΨ[Trl]GlyΨ[Trl]-; -GlyΨ[Trl]GlyΨ[Trl]GlyΨ[Trl]-; and the one containing two triazole units, -GlyΨ[Trl]GlyΨ[Trl]-, seem to provide the best spacing. This bridge is present in the strongest derivative presented here, H-Lys(hArg)-GlyΨ[Trl]GlyΨ[Trl]Arg-OH (cmpd **3**, *inh* = 58.1%, IC_50_ = 8.39). The isomer of this compound with D-Lys at the first position exhibits a slightly lower inhibition **4** (*inh* = 52.6%, IC_50_ = 10.22). If there is a simultaneous exchange for D-Har at the branched side-chain **5**, (*inh* = 48.5%, IC_50_ = 9.11), the inhibition is in between. As to the remaining bridges of the “linker” series, shorter derivatives **1** and **2** (containing -GlyΨ[Trl]Arg) are significantly worse inhibitors (~30% of inhibition). Elongating this spacer by a natural AA, either before or after the triazole-AA, usually improves the inhibition, but not to a large extent and not in each case (**6**–**11**).

Having identified -GlyΨ[Trl]GlyΨ[Trl]- as the optimal spacer, we conceived the design of several analogues of structure **3**, in which the N-terminal residue is varied (“arm” subseries). Here, it turned out that free amine at the Har residue is not of crucial importance. When it is masked by Fmoc (**14**, *inh* = 57.7%), there is no drop in inhibitory activity compared with compound **3**. Some decrease upon Fmoc-masking of this amine, however, is observed in the derivative pair with D-Lys at the first position (**15**, *inh* = 43.7%).

The shortening of the first position’s side chain is adverse to activity (**17**, *inh* = 41.3%; **18**, *inh* = 25.3%). Additionally, it is to be noted that 6Ahx, which replaces Lys in this derivative, differs from the latter by the lack of an α-NH_2_ group. In fact, the lack of the N-terminal amine group seems to be deteriorating to activity as the derivative with 6-aminohexanoic acid (**19**, *inh* = 30.5%) and 5-aminopentanoic acid (**20**, *inh* = 29.7%) are significantly worse than the parent.

Derivatives in which Har is attached to the backbone (via Cα and not via the side-chain) are significantly worse than the parent **3** (cmpds **12**, **16**, **21**, **22**, **23**, inhibitory activity in the range of 9.2% to 36.5%), indicating that Har residue must be attached at the side chain of the lysine.

#### Correlation Analysis

The discussed observations are also quantitatively captured by correlation analysis. Herein, we correlated activity against variables describing the presence/absence of particular structural features and other structural characteristics. The former were accounted for by descriptors of binary type (with values, 1—presence and 0—absence). Other types of variables included topological distance between important structural features. The descriptor matrix used for the analysis is provided in Supporting Materials (Table SM-COR-1).

**Figure 4:**
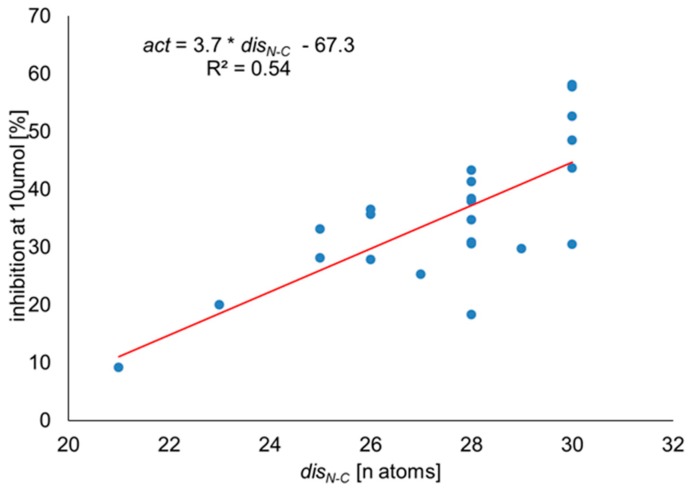
Correlation of inhibitory activity at 10 μM with the topological distance between guanidine groups at the N- and C-termini of the ligands (Model 1).

According to the correlation analysis, a single structural factor able to explain as much as 54% (Model 1, coefficient of determination, R^2^ = 0.54, Figure 4) of the observed variance is the topological distance between guanidine groups at the N- and C-termini of the ligands (*dis_N-C_*). Thus, within our set of compounds, the larger the separation of the guanidines, the higher the observed inhibitory activity. Including more explanatory variables obviously improves the model (listing SM-COR-1); however, owing to a rather small number of points analyzed, this improvement cannot be stated to be statistically robust. Nevertheless, as the variables included are structurally well-understood, the models provide at least approximate explanation of the tendencies.

**Figure 5:**
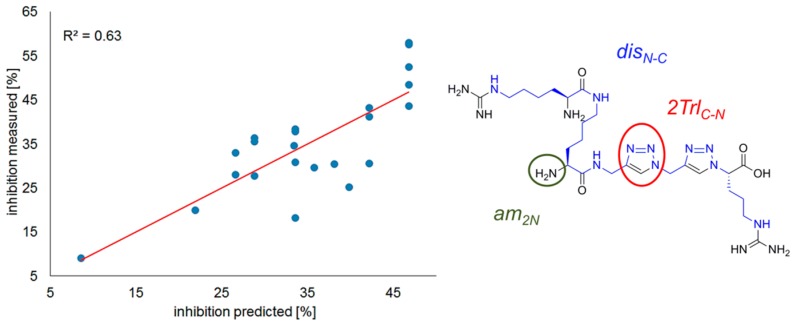
Correlation of observed vs. predicted (Model 2) inhibitory activity with marked variables.

The best tradeoff between quality of fit and number of variables is obtained when three variables are used (Model 2, Figure 5): (a) (normalized) topological distance between guanidine groups at the N- and C-termini of the ligands (*dis_N-C, norm_*); (b) triazole unit instead of the second amide/peptide bond (C to N direction) (*2Trl_C-N_*); (c) free amine present by the second amide/peptide bond (going from the N-terminus or from the residue attached to the N-terminal side chain) (*am_2N_*). The model equation reads as follows:

*inh* = 8.6 (± 5.0) + 20.9 (± 8.6) * *dis_N-C, norm_ +* 8.7 (± 4.5) * *am_2N_* + 8.6 (± 4.6) * *2Trl_C-N_* R^2^ = 0.63, n = 23. (1)

The high coefficient of *dis_N-C, norm_* once again stresses the importance of the guanidines’ separation. Some positive influence on activity is exerted by triazole unit at the second position counting from C to N (*2Trl_C-N_*) and by a free amine at the second position counting from N to C (*am_2N_*).

### 2.3. Molecular Modelling

In order to rationalize the obtained activity trends in structural terms, we have attempted to model complex of neuropilin-1 with compound **3** by means of molecular dynamics simulations. The starting binding mode was chosen by superimposing the compounds’ arginine residue on the arginine residue of tuftsin in 2ORZ crystallographic structure [43]. The remaining elements of compound **3** (Lys(Har)-GlyΨ[Trl]GlyΨ[Trl]-) were added manually so that they were in an all-extended conformation (pointing to the solvent bulk). Three simulations (SIM-I, SIM-II, and SIM-III) with production runs of 420 ns in length were carried out with the expectation of finding a reliable binding mode for the compound.

**Figure 6:**
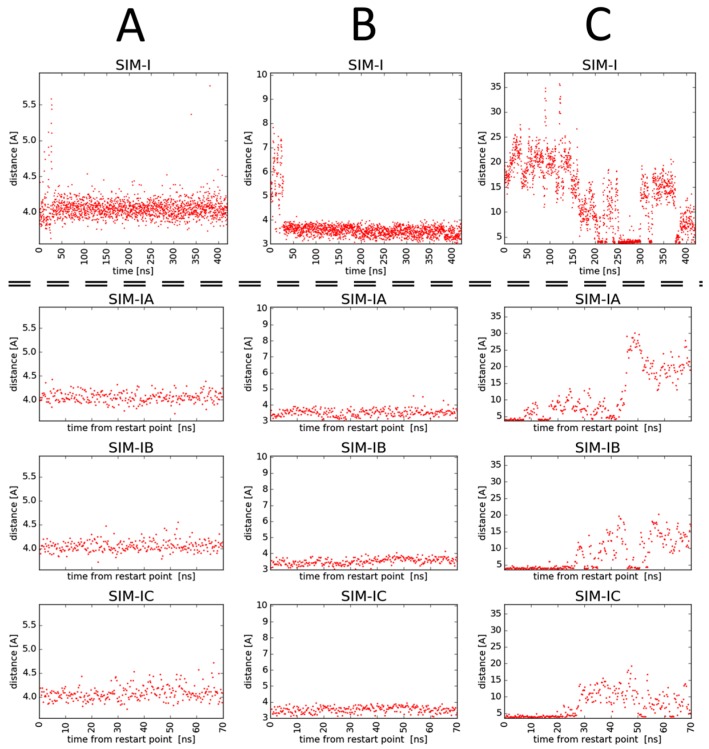
Time evolution of distances between (**A**) Cγ atom of Asp320 and Cζ of Arg residue in compound **3**; (**B**) Oγ atom of Ser346 and C of Arg residue in compound **3**; (**C**) Cδ atom of Glu319 and Cζ of Har residue in compound **3**. Data come from simulations SIM-I, SIM-IA, SIM-IB, and SIM-IC.

In all three simulations, the guanidine group of the C-terminal arginine kept an ionic interaction with Asp320 (Figure 6A and SM-SIM-1, Table SM-SIM-1). In SIM-I, it has also maintained the hydrogen bonds of the free carboxylate with Ser346 and Thr349 (Figure 6B). In the other two (SIM-II—Figure SM-SIM-4-F-H and SIM-III—Figure SM-SIM-4-I-J), the arginine rotated in the manner that, while keeping the ionic contact with Asp320, the carboxylate was exposed to the solvent (Figure SM-SIM-2).

**Figure 7:**
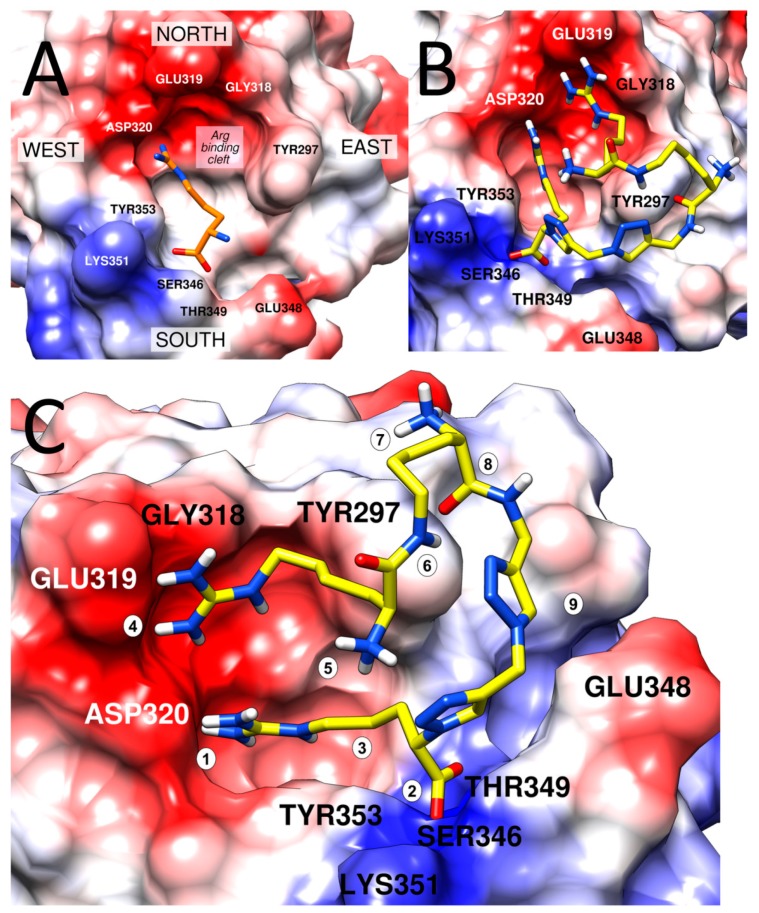
Binding site at the neuropilin-1. The protein is depicted as an electrostatic colour-coded surface (red: negative charges, white: neutral, blue: positive). (**A**) The binding site presented with the conventional assignment of directions as introduced in our previous contributions [28,29,30]. C-terminal arginine residue (orange, found in our inhibitors, but also in VEGF (4DEQ [44]) or tuftsin (2ORZ [43]) or arginine derivatives (5JGI, 5IYY, 5J1X, 5JGQ, 5JHK [45]) is shown in the cleft it occupies in crystal structures as well in simulations. (**B**) Compound **3** (yellow) at the NRP-1 binding site. A representative snapshot from SIM-I showing the discussed binding mode. Top projection. (**C**) Compound **3** (yellow) at the NRP-1 binding site. A representative snapshot from SIM-I showing the discussed binding mode. Rotated top projection. Marked are characteristic elements of the binding mode. 1—ionic interaction of Arg guanidine with Asp320; 2—hydrogen bonds of free carboxylate with Ser346 and Thr349; 3—Arg chain buried within the cleft, extended between Tyr353 and Tyr297, further apolar interactions with Trp301, Thr316, Gly414, Ile415, and Ser416; 4—Har interacts with Glu319 and Gly318; 5—Har free amine exposed to the solvent; 6—H of the amide bond interacts either with phenolic function of Tyr297 or forms an intramolecular hydrogen bond with triazole ring (N3); 7—Lys free amine exposed to the solvent; 8—carbonyl of the peptide bond forms an H-bond to the phenolic function of Tyr297; 9—C5H of the triazole ring is able to be involved in the H-bond with Glu348 depending on the rotameric state of this residue.

The ‘linker’ and the ‘arm’ parts of the inhibitor molecule perambulated over the protein surface, forming short-lived contacts with diverse residues. Several interaction patterns were more stable or appeared more than once. In SIM-I, the ‘arm’ tended to interact with the residues of the conventional ‘north’ of the binding site, while in SIM-II and SIM-III, the Har residue was directed to the ‘south-east’ (Figure 7A for explanation of the convention, Figures SM-SIM-4-F-J for representative examples from SIM-II and SIM-III).

According to the crystallographic [43,44,45], mutagenetic [21], and molecular modelling studies (including our previous contributions [24,26,28,29,30]), three contacts of Arginine residue with Asp320, Ser346, and Thr349 are particularly important for binding of inhibitors with NRP1; therefore, we have decided to focus on SIM-I in which all three of these were maintained.

Three other replicas were run starting from the snapshot of SIM-I at t = 220.0 ns, because around this time point, there appeared a relatively stable binding pose (Figure 7B,C). In this configuration, the ligand formed the following contacts with the NRP1:

- Ionic interaction of C-terminal Arg guanidine with Asp320;
- Hydrogen bonds of C-terminal Arg carboxylate with Ser346 and Thr349;
- Dispersive contacts of C-terminal Arg aliphatic portion with the residue lining the binding cleft Tyr353, Tyr 297, Trp301, Thr316, Gly414, Ile415, and Ser416;
- Ionic interaction of Har guanidine with Glu319 or an H-bond of this moiety with carbonyl group of Gly318.

Additionally, hydrogen of the amide bond joining Har and Lys formed an H-bond with the phenolic function of Tyr297 or an intra-molecular hydrogen bond with 2Trl_C-N_. Furthermore, this triazole had transient H-bond interactions with Glu348 (via C5H). Another transient contact included H-bond between carbonyl of the peptide bond between Lys and Gly[Trl] and the phenol moiety of Tyr297.

It seems that this binding mode could be useful for explaining some of the SAR trends for our triazolopeptides. First, the stereochemistry of the Lys residue is roughly neutral to activity. The amine moiety does not form interactions with the protein. Configuration inversion at Lys Cα could be compensated by rotations of the arm’s flexible bonds so that the Har interactions with Glu319/Gly318 are preserved.

Second, masking of Har α-amine by Fmoc moiety (**14**, **15**) also does not affect activity, as the amine interacts neither with the protein, nor with any elements of the peptide.

Further, exchanging Lys for 5Ava/Dab/Dap (shorter, cmpds **17**, **18**, **20**) changes the position of the amide bond in the branched side-chain. In this way, formation of the intramolecular H-bond (Figure 7; characteristic element 6) or interaction with Tyr297 is distorted.

Assuming that the presented interaction mode is optimal for the compounds reported here, it is clear why Har is preferably attached by the side-chain rather than the main chain. Side-chain attachment allows for a more facile reaching of the negatively charged elements at the ‘northern’ wall of the binding cleft.

Still, the presented interaction mode was stable for only 20%, 32%, 52%, and 45% of simulation times of SIM-I, SIM-IA, SIM-IB, and SIM-IC, respectively (note that the latter three are shorter and restarted from a snapshot with this very configuration). This relative instability of the binding mode found in molecular dynamics (MD) simulations seems consistent with only moderate inhibitory activity of the compound **3**. The simulations reveal that even when the arginine residue sits tight in the cleft, the triazole-based linker and the Lys(Har) arm retain significant mobility, which compete against stable inhibitor–protein interactions. Thus, improving the inhibitory activity for peptidomimetics of the type presented here could be presumably achieved by introducing elements decreasing the conformational freedom and/or enabling additional interactions with the protein.

### 2.4. In Vitro Proteolysis of Triazolopeptides

Two of the most potent triazolopeptides, compounds **3** and **4**, were investigated as to their proteolytic resistance by incubation in human plasma at 37 °C. The samples of the incubated solutions were taken at constant time intervals (every 8 h) and analyzed with the use of HPLC-MS. This technique allows for determination of the proteolysis rate, and enables finding the cleavage sites by structural determination of appearing metabolites. To this aim, we used a previously established protocol [29].

**Figure 8:**
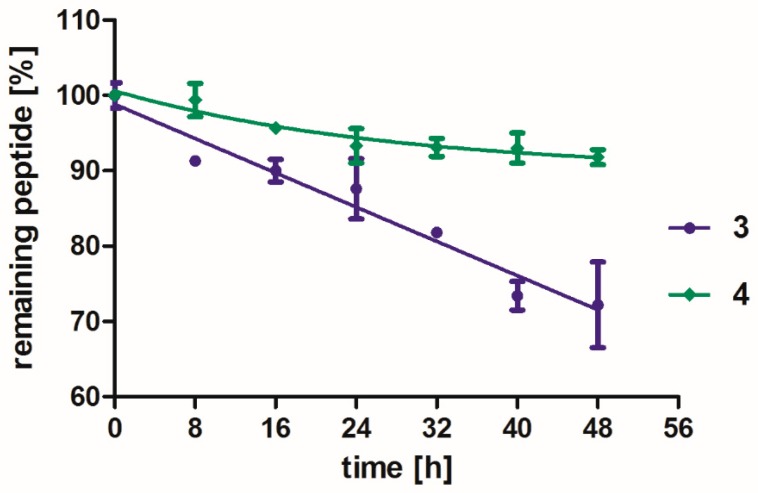
In vitro stability toward proteolysis of two triazolopeptides **3** violet line and **4** green line. Each point is an average result ± SD calculated from three independent experiments.

Figure 8 shows the results of the measurements. During the monitored experiment time, both compounds were stable toward proteolytic cleavage and not fully degraded. In the case of **3**, after 48 h of incubation, ca. 70% of compound remained in the solution and **4** was almost non-degraded. The experiments were stopped after 48 h because of water evaporation/condensation processes in the Eppendorf tube, as these might affect the sample concentration and assay readouts.

The only observed cleavage occurs at the “arm” site of the molecule (Lys(Har)-XXX) between lysine side chain and homo-arginine residue. The difference between stability of these two compounds is related to exchange of L-Lys (**3**) into D-Lys (**4**). Triazole rings were not metabolized within the monitored time of the experiment.

The compounds **3** and **4** exhibit improved stability (t_1/2_ >> 48 h) as compared with our previously reported Lys(Har)-Dab/Dap-Pro-Arg (t_1/2_ = 34 h/41 h) and Lys(Har)-Pro-Ala/Dab-Arg (t_1/2_ = 39 min/44 min) [29]. Thus, it can be concluded that introducing triazole isosteres of the peptide bonds is a good way for improving the proteolytic stability of NRP-1/VEGF_165_ interaction inhibitors. Within the studied series, the best stability with optimal inhibitory activity is achieved when two triazole ring segments and D-Lys at the N-terminal position are simultaneously present.

### 2.5. In Vitro Cell Survival Test

Because of the fact that **4** was observed to be relatively resistant toward proteolysis, one should consider a scenario when the compound stays in the circulation for a prolonged time. For this reason, we carried out a preliminary cytotoxicity assay on murine progenitor cells (myeloblast-like cell line derived from long term cultures of bone marrow 32D) to investigate the impact of **4** at different concentrations on their viability.

**Figure 9:**
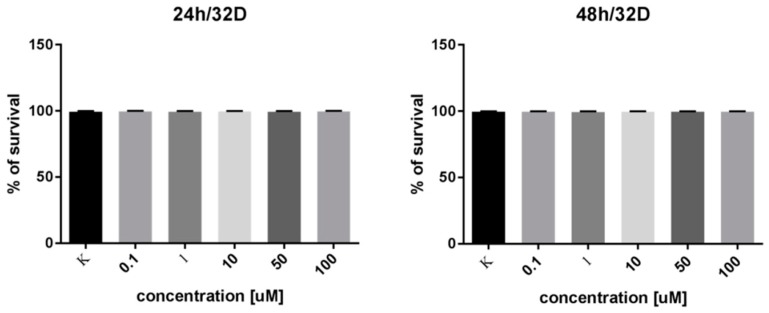
Survivability of myeloblast-like cell line derived from bone marrow at different concentrations of **4**. No significant differences in cell survival using a *t*-test were shown.

According to the results shown in Figure 9, compound **4** does not exhibit any significant impact on cell survivability at relatively high (100 µM—one order of magnitude higher than IC_50_ value) dose and long-term (48 h) incubation. This might give some indication that the triazolopeptides are safe for normal cells; however, further research on a number of different cell types should be undertaken.

## 3. Materials and Methods

### 3.1. Synthesis

All solvents were purchased from local commercial suppliers: DMF, DCM, MeOH, and Et_2_O (puriss) were used without further purification; THF (puriss) was freshly distilled before the use according to the standard protocols. Fmoc protected amino acids and coupling reagents were purchased from Iris Biotech GmbH. Wang resin with preloaded Fmoc-Arg(Pbf) with a capacity of 0.57 mmol/g was obtained from Activotec (Comberton, Cambridge, UK). Propargylamine was purchased from Sigma-Aldrich and Fmoc-OSu was obtained from Merck. HPLC grade solvents can and MeOH were purchased from Merck.

All compounds **1**–**23** were obtained manually using mixed solid phase peptide synthesis and solution phase according to standard coupling procedures for Fmoc/tBu strategy. The quantity of Wang polystyrene resin was fixed to carry out the synthesis in a scale of 0.15 mmol.

#### 3.1.1. Wong Diazotransfer

During a typical reaction procedure, 1 mL of distilled H_2_O sodium azide (98 mg, 1.5 mmol) was dissolved followed by the addition of 1.5 mL of DCM. The mixture was cooled in an ice bath, following which trifluoromethanesulfonic anhydride (85 mg, 53 µL, 0.3 mmol) was added dropwise for approximately five minutes. The reaction was carried out in an ice bath for two hours. After this time, the reaction mixture was transferred to a separatory funnel and the DCM phase was removed. The aqueous phase was extracted twice with 1 mL of DCM. The organic phase was then combined and washed once with saturated sodium carbonate solution and used for diazotransfer on solid support without any further purification. For this purpose, to the reaction vessel with resin beads, a previously described organic phase with 15 was added followed by 1 mL of MeOH and potassium carbonate (21 mg, 0.15 mmol) and copper (II) sulfate pentahydrate (0.25 mg, 1.0 µmole) dissolved in 1 mL of distilled water. The reaction was left overnight. ompletion of the reaction was determined with standard Kaiser test, and we observed a negative result.

#### 3.1.2. Fmoc-Propargylamine (**24**) Preparation

A suspension of N-(9H-fluoren-9-ylmethoxycarbonyloxy) succinimide (338 mg, 1.0 mmol) in 4 mL of freshly dried and distilled THF was cooled with an ice bath, and propargylamine (44 mg, 54 µL, 0.8 mmol) was added dropwise. The reaction mixture was stirred and allowed to warm to room temperature over 2 h. The solvent was removed under reduced pressure to give a residue. The residue was dissolved in 20 mL of EtOAc, transferred to a separatory funnel, and washed a few times with 10 mL of distilled water. The organic layer was dried and the solvent was evaporated, affording 155 mg (0.56 mmol, yield 70%) of white/pale yellow needles.

Fmoc-Propargylamine (24): 1H NMR (DMSO, 400 MHz) δ A 7.89 (2H, d, *J* = 7.6 Hz, Fmoc H4 and H5), B 7.79 (1H, t, *J* = 5.8 Hz, NH), C 7.69 (2H, d, *J* = 7.4 Hz, Fmoc H1 and H8), D 7.33 (2H, td, *J* = 7.6, 1.3, Fmoc H2 and H7), E 7.41 (2H, td, *J* = 7.6, 1.2 Hz, Fmoc H3 and H6), F 4.31 (2H, d, *J* = 7.0 Hz, Fmoc CH2), G 4.22 (1H, t, *J* = 6.9 Hz, Fmoc H9), H 3.77 (2H, dd, *J* = 5.8, 2.5 Hz, H3), I 3.11 (1H, t, *J* = 2.5 Hz, H1); 13C NMR (DMSO, 100 MHz) δ 156.02 (C), 143.87 (C), 143.87 (C), 140.80 (C), 140.80 (C), 127.73 (CH), 127.73 (CH), 127.17 (CH), 127.17 (CH), 125.23 (CH), 125.23 (CH), 120.23 (CH), 120.23 (CH), 81.46 (C), 73.14 (CH), 65.74 (CH2), 46.66 (CH), 29.84 (CH2).

#### 3.1.3. Click Reaction on Solid Support

In 4 mL of THF, a Fmoc-propargylamine (83 mg, 0.3 mmol) was dissolved and the mixture was transferred to the reaction vessel filled with resin beads. In the next step, the copper (II) sulfate pentahydrate (7.5 mg, 0.03 mmol) was added to 2 mL of distilled water. Both aqueous and organic solutions were combined with the resin and a portion of sodium ascorbate (20 mg, 0.1 mmol) was poured to the reaction vessel, after which the color of the solution changed from pale blue to bright yellow. The reaction was left for at least 10 h in an argon atmosphere. Completion of reaction was determined by a modified Kaiser test [40]. A few drops of 5% solution of triphenyl phosphine in THF and distilled water were added to the resin sample and heated in a closed vessel on a hot plate. After that, the standard Kaiser test solution was placed inside the vial, gently heated, and shaken. After cooling down, the negative result was noticed.

#### 3.1.4. Common Procedures for Peptide Chain Elongation on Solid Support

Standard Fmoc deprotection was carried out stepwise with 20% piperidine in DMF through resin shaking for 5 min and a further 20 min with a fresh portion of reagent. For amino acids’ coupling, a three-fold excess of Fmoc-protected amino acid, three-fold excess of OxymaPure, and three-fold excess of DIC were mixed in DMF and added to the solid support. After 60 min of shaking, completion of the reaction was confirmed by a negative result for the Kaiser test.

#### 3.1.5. Guanylation Reaction of Lys to Gain Har

Guanylation reaction was carried out according to the protocol described in the literature [41]. The peptidyl resin with the deprotected ε-amino group was treated with 10 eq. of 3,5-dimethylpyrazole-1-carboxamidine nitrate dissolved in DMF and adjusted pH to ca. 10–11 by means of DIPEA. The reaction was left under constant shaking for 72 h to fully convert lysine into homoarginine.

#### 3.1.6. Peptidotriazole Cleavage from Solid Support

After synthesis completion, the resin was washed trice with DCM, after that with MeOH, and finally with Et_2_O. The resin was further dried under reduced pressure and treated with cleavage cocktail mixed from TFA/TIS/H_2_O (0.95:2.5:2.5 in volume ratio). The reaction was left for at least 3 h and after that, the resin was filtered and washed trice with a fresh portion of TFA. The supernatants’ volume was then reduced and a large excess of cold Et_2_O (at least −20 °C) was added to the vessel to precipitate crude compound, which was centrifuged, decanted, washed trice with fresh Et_2_O, and dried.

#### 3.1.7. HPLC Analysis, Purification, and HRMS Characterization

The crude peptidotriazoles were analyzed and purified on RP-HPLC analytical column Jupiter Proteo I.D. 4.6 mm × 250 mm and preparative column Jupiter Proteo I.D. 21.2 mm × 250 mm. Phase A was ultrapure water + 0.1% volume TFA, phase B was gradient grade acetonitrile + 0.1% volume TFA. For all compounds, three types of linear gradient elution were used: ^1^linear increase of phase B from 1% to 21% in 20 min; ^2^linear increase of phase B from 10% to 40% in 20 min; and ^3^linear increase of phase B from 20% to 40% in 20 min. Detection was carried out at 220 nm. Analytical flow = 1 mL/min, preparative flow = 20 mL/min. Fractions containing pure peptidotriazoles were freeze dried. Pure compounds were then analyzed using high-resolution mass spectrometry to confirm their general formula.

### 3.2. ELISA Assays of Inhibitory Activity

This assay was carried out according to a previously described method [29].

### 3.3. Correlation Analysis

The observed inhibitory activity at 10 μmol inhibitor concentration was correlated against several sets of structural descriptors. The sets included indicator variables, in which presence or absence of diverse structural elements was expressed as 1 or 0, respectively. Further, included was the topological distance between the guanidine groups at the N- and C-termini of the ligands (dis_N-C_), that is, the number of atoms separating the two groups. If normalized, the variables were recalculated so that the smallest value of the set assumed 0 and the largest 1. The variable matrix is given in Supplementary Materials (Table SM-COR-1).

The multivariable linear correlations were performed using Microsoft Excel.

### 3.4. Molecular Dynamics

The complex of compound **3** with NRP-1 was studied by molecular dynamics (MD) in AMBER [46]. The starting structure was prepared by superposing C-terminal Arg of compound **3** onto the coordinates of Arg residue’s atoms of tuftsin (TKPR), as found in 2ORZ crystallographic structure [43]. The remaining parts of compound **3** were added manually so that they were in all-extended conformation. AmberTools [46] was used to solvate the complex and to neutralize the system by adding Na^+^ and Cl^-^ ions. The protonation states (in ligands and in the protein) were set as assumed in pH = 7. Ff14SB force field [47] was used with TIP3 water model. Parameters for triazole-based residues were taken from the work of Marion et al. [48].

The system was minimized and equilibrated (NVT). Then, the production step (NPT ensemble, T = 303.15 K, integration step = 2 fs, cut-off scheme Verlet, Nose–Hoover thermostat, Parrinello-Rahman barostat, LINCS H-bonds constraints) followed. In the first set, three independent simulations of 420 ns production were run (SIM-I, SIM-II, SIM-III). Another series included three simulations started from a SIM-I snapshot at t = 220.0 ns. These lasted 70 ns.

The analyses of the results were performed using AmberTools and in-house Python scripts (using MDAnalysis library [49]). The molecular graphics were prepared in UCSF Chimera [50].

### 3.5. Stability

The human plasma was provided by Mossakowski Medical Research Centre Polish Academy of Sciences (venous blood was obtained from three healthy donors and plasma samples were pooled in equal volumes). Triazolopeptide stock solution was prepared to achieve concentration ca. 1.4 mg/mL. Then, this solution was diluted two-fold into human plasma, affording a final concentration of ca. 1.1 µmol/mL. The samples were mixed on vortex mixer and incubated at 37 °C with 700 rpm mixing in thermoblock. Samples of 100 µL were taken every 8 h; the enzymatic cleavage was quenched by addition of 200 µL of 15% aqueos trichloroacetic acid and vortexed for a while. Precipitated proteins were then centrifuged at 14,000 rpm and 4 °C for 10 min to pellet them. After that, 150 µL of supernatant was taken and analyzed on HPLC-MS (quadrupole mass analyzer and electrospray ionization source). The system was equipped with Jupiter Proteo column I.D. 2.0 mm × 250 mm, phase A: ultrapure water + 0.05% TFA, phase B: gradient grade acetonitrile + 0.05% TFA, detection at 210 nm, quadrupole fixed on scan mode. Elution was with linear increase from 0% to 15% of phase B in 30 min, flow = 0.2 mL/min. All samples were analyzed trice in independent experiments.

### 3.6. In Vitro Cell Survival Test

For the cell survivability test, the myeloblast-like cell line derived from long term cultures of bone marrow (32D, ATCC #CRL-11346) was used. Experiments were carried out using Muse Count & Viability Kit and Muse Cell Analyzer (Merck Millipore) according to the procedure given. Cells were incubated for 24 or 48 h with given **4** concentration. Each experiment was performed in three technical replications in three independent experiments. Graphs were made with the use of GraphPad Prism.

## 4. Conclusions

In previous works, we disclosed several submicromolar (tetrapeptides and branched pentapeptides) inhibitors of the interaction between neuropilin-1 and vascular endothelial growth factor. In the present contribution, we describe follow-up research whose intent was to see whether the introduction of 1,4-disubstituted 1,2,3-triazole rings as peptide bond isosteres could give derivatives with improved proteolytic stability that still retain the inhibitory activity.

Thus, we report here 23 novel triazolopeptides along with their inhibitory activity. The structural variation within this set focused on modifying the “linker” part and the “arm” part of the molecules. The designed derivatives were obtained on solid support, mostly following typical procedures. Less usual, but very convenient was that the introduction of the triazole to the sequence went completely on solid support. By an adjustment of the Wong diazotransfer method, we were able to convert amines into azides directly on peptidyl-resin.

As to the activity, it spans from 9.2% to 58.1% at a concentration of 10 μM. The best three compounds include Lys(Har)-GlyΨ[Trl]GlyΨ[Trl]Arg (**3**, IC_50_ = 8.39 μM), D-Lys(Har)-GlyΨ[Trl]GlyΨ[Trl]Arg (**4**, IC_50_ = 10.22 μM), and D-Lys(D-Har)-GlyΨ[Trl]GlyΨ[Trl]Arg (**5**, IC_50_ = 9.11 μM)). None of the triazolopeptides are better than the reference A7R (ATWLPPR, IC_50_ = 5.86 μM). An analysis of the “linker” subseries allows for the conclusion that the best activity is obtained with -GlyΨ[Trl]GlyΨ[Trl]- in the middle part of the molecule. The stereochemistry at the N-terminal residue (Lys) is not critical. It seems to be more important that the “arm” construction, the derivatives in which Har is attached via the ‘backbone’, are worse than those where the residue is linked via a side-chain amide bond. The shortening of the ‘arm’ is adverse to activity. Thus, within the studied series, the optimal ‘arm’ is Lys(Har)- and other stereovariants thereof are not much worse.

Most of the observed trends in SAR can be explained by a binding mode based on molecular dynamics simulations of compound **3**. Further, the modelling points to large flexibility of the triazolopeptides studied herein. Its conformational freedom competes with the formation of stable protein–inhibitor interactions. Therefore, it seems that the introduction of elements decreasing the conformational space within the ‘linker’ part or forming additional interactions (e.g., branched or charged side-chains) may improve the affinity of NRP-1.

Compounds **3** and **4** were assayed for proteolytic stability. It turned out that both are remarkably stable. At 48 h after the start of the experiment, derivative **4** (with D-Lys(Har)- in the first position) was almost undigested, and in the case of derivative **3** (Lys(Har) in the first position), more than 70% of the substance remained in the sample. The only observed cleavage occurred at the N-terminal site of the molecule and the triazole rings were not metabolized. Thus, compounds **3** and **4** exhibit significantly improved stability compared with our previous inhibitors. Moreover, compound **4** does not exhibit any significant impact on normal cells’ viability at a relatively high concentration (100 µmol) and long-term of incubation (48 h). Our results indicate that triazolopeptides 3 and 4 can be the starting structures for further modification to search for more active VEGF/NRP inhibitors.

## Figures and Tables

**Table 1:** Systematic characterization of synthesized triazolopeptides **1**–**23**.

No.	Sequence	RT [min.]	General Formula	Calc. MS [*m*/*z*]	Meas. MS [*m*/*z*]
**“linker” subseries**					
1	Lys(Har)-GlyΨ[Trl]Arg	11.98 ^1^	C22H43N13O4	554.3634 ^4^	554.3640 ^4^
2	D-Lys(Har)-GlyΨ[Trl]Arg	11.96 ^1^	C22H43N13O4	554.3634 ^4^	554.3656 ^4^
3	Lys(Har)-GlyΨ[Trl]GlyΨ[Trl]Arg	13.22 ^1^	C25H46N16O4	635.3961 ^4^	635.3979 ^4^
4	D-Lys(Har)-GlyΨ[Trl]GlyΨ[Trl]Arg	13.30 ^1^	C25H46N16O4	635.3961 ^4^	635.3980 ^4^
5	D-Lys(D-Har)-GlyΨ[Trl]GlyΨ[Trl]Arg	13.33 ^1^	C25H46N16O4	635.3961 ^4^	635.3982 ^4^
6	Lys(Har)-Pro-GlyΨ[Trl]Arg	14.65 ^1^	C27H50N14O5	651.4161 ^4^	651.4187 ^4^
7	D-Lys(Har)-Pro-GlyΨ[Trl]Arg	14.65 ^1^	C27H50N14O5	651.4161 ^4^	651.4190 ^4^
8	Lys(Har)-Phe-GlyΨ[Trl]Arg	14.89 ^2^	C31H52N14O5	701.4318 ^4^	701.4339 ^4^
9	D-Lys(Har)-Phe-GlyΨ[Trl]Arg	15.04 ^2^	C31H52N14O5	701.4318 ^4^	701.4338 ^4^
10	Lys(Har)-GlyΨ[Trl]Ala-Arg	13.17 ^1^	C25H48N14O5	625.4005 ^4^	625.3994 ^4^
11	Lys(Har)-GlyΨ[Trl]Gly-Arg	12.19 ^1^	C24H46N14O5	611.3848 ^4^	611.3878 ^4^
12	Har-Pro-GlyΨ[Trl]Arg	13.78 ^1^	C21H38N12O4	523.3212 ^4^	523.3221 ^4^
13	Har-GlyΨ[Trl]GlyΨ[Trl]GlyΨ[Trl]Arg	14.52 ^1^	C22H37N17O3	588.3338 ^4^	588.3362 ^4^
**“arm” subseries**					
14	Lys(Fmoc-Har)-GlyΨ[Trl]GlyΨ[Trl]Arg	16.50 ^3^	C40H56N16O6	429.2357 ^5^	429.2371 ^5^
15	D-Lys(Fmoc-Har)-GlyΨ[Trl]GlyΨ[Trl]Arg	16.53 ^3^	C40H56N16O6	429.2357 ^5^	429.2373 ^5^
16	Har-Lys-GlyΨ[Trl]GlyΨ[Trl]Arg	13.05 ^1^	C25H46N16O4	635.3961 ^4^	635.3991 ^4^
17	Dab(Har)-GlyΨ[Trl]GlyΨ[Trl]Arg	12.46 ^1^	C23H42N16O4	607.3648 ^4^	607.3675 ^4^
18	Dap(Har)-GlyΨ[Trl]GlyΨ[Trl]Arg	11.92 ^1^	C22H40N16O4	593.3491 ^4^	593.3517 ^4^
19	Har-6Ahx-GlyΨ[Trl]GlyΨ[Trl]]Arg	16.83 ^1^	C25H45N15O4	620.3852 ^4^	620.3878 ^4^
20	Har-5Ava-GlyΨ[Trl]GlyΨ[Trl]Arg	15.23 ^1^	C24H43N15O4	606.3695 ^4^	606.3721 ^4^
21	Har-Ala-GlyΨ[Trl]GlyΨ[Trl]]Arg	13.46 ^1^	C22H39N15O4	578.3382 ^4^	578.3406 ^4^
22	Har-Gly-GlyΨ[Trl]GlyΨ[Trl]Arg	13.06 ^1^	C21H37N15O4	564.3226 ^4^	564.3244 ^4^
23	Har-GlyΨ[Trl]GlyΨ[Trl]Arg	13.06 ^1^	C19H34N14O3	507.3020 ^4^	507.3011 ^4^

^1^ linear increase of phase B from 1% to 21% in 20 min; ^2^ linear increase of phase B from 10% to 40% in 20 min; ^3^ linear increase of phase B from 20% to 40% in 20 min; ^4^ pseudomolecular ion [M + H]^+^; ^5^ pseudomolecular ion [M + 2H]^2+^.

**Table 2:** Inhibitory activity of the synthesized triazolopeptides.

No	Sequence	Inhibition of bt-VEGF_165_ Binding to NRP-1 [10 µM] ^1^	IC_50_ [µM]
A7R	Ala-Thr-Trp-Lys-Pro-Pro-Arg	61.0 ± 0.4 ^3^	5.86 ^3^
KPPR	Lys-Pro-Pro-Arg	64.5	4.60 ^4^
	Lys(Har)-Dap/Dab-Pro-Arg	96.8/98.5	0.2/0.2 ^4^
**“linker” subseries**			
1	Lys(Har)-GlyΨ[Trl]Arg	28.1 ± 1.2	-
2	D-Lys(Har)-GlyΨ[Trl]Arg	33.1 ± 1.9	-
3	Lys(Har)-GlyΨ[Trl]GlyΨ[Trl]Arg	58.1 ± 2.1	8.39 ^2^
4	D-Lys(Har)-GlyΨ[Trl]GlyΨ[Trl]Arg	52.6 ± 1.3	10.22 ^2^
5	D-Lys(D-Har)-GlyΨ[Trl]GlyΨ[Trl]Arg	48.5 ± 2.9	9.11 ^2^
6	Lys(Har)-Pro-GlyΨ[Trl]Arg	18.3 ± 0.9	-
7	D-Lys(Har)-Pro-GlyΨ[Trl]Arg	38.4 ± 1.5	-
8	Lys(Har)-Phe-GlyΨ[Trl]Arg	30.9 ± 2.1	-
9	D-Lys(Har)-Phe-GlyΨ[Trl]Arg	37.9 ± 1.1	-
10	Lys(Har)-GlyΨ[Trl]Ala-Arg	43.2 ± 1.5	-
11	Lys(Har)-GlyΨ[Trl]Gly-Arg	30.6 ± 1.1	-
12	Har-Pro-GlyΨ[Trl]Arg	9.2 ± 0.8	-
13	Har-GlyΨ[Trl]GlyΨ[Trl]GlyΨ[Trl]Arg	34.8 ± 1.5	-
**“arm” subseries**			
14	Lys(Fmoc-Har)-GlyΨ[Trl]GlyΨ[Trl]Arg	57.7 ± 1.8	-
15	D-Lys(Fmoc-Har)-GlyΨ[Trl]GlyΨ[Trl]Arg	43.7 ± 0.5	-
16	Har-Lys-GlyΨ[Trl]GlyΨ[Trl]Arg	36.5 ± 1.4	-
17	Dab(Har)-GlyΨ[Trl]GlyΨ[Trl]Arg	41.3 ± 0.5	-
18	Dap(Har)-GlyΨ[Trl]GlyΨ[Trl]Arg	25.3 ± 3.5	-
19	Har-6Ahx-GlyΨ[Trl]GlyΨ[Trl]Arg	30.5 ± 0.6	-
20	Har-5Ava-GlyΨ[Trl]GlyΨ[Trl]Arg	29.7 ± 1.8	-
21	Har-Ala-GlyΨ[Trl]GlyΨ[Trl]Arg	27.8 ± 2.1	-
22	Har-Gly-GlyΨ[Trl]GlyΨ[Trl]Arg	35.7 ± 1.6	-
23	Har-GlyΨ[Trl]GlyΨ[Trl]Arg	20.0 ± 2.1	-

^1^ Percentage value for inhibition of vascular endothelial growth factor 165 (VEGF_165_) binding to neuropilin-1 (NRP-1) (P) was calculated according to the equation P = 100% − [(S − NS)∙100%/(P − NS)], where S is signal intensity measured, N is signal measured in negative control, and P is the maximum binding signal obtained with (bt)-VEGF-A165 without triazolopeptide **1**–**23**. The values represent mean ± S.D of at least two independent experiences performed in triplicate; ^2^ **3** logIC_50_ = −5.076 ± 0.06, R^2^ = 0.9859; **4** logIC_50_ = −4.991 ± 0.05, R^2^ = 0.9921; **5** logIC_50_ = −5.040 ± 0.1417, R^2^ = 0.9358; ^3^ values taken from the work of [24]; ^4^ values taken from the work of [29].

## Supplementary Materials

Supplementary Materials (SM) are available online, SM file is divided into sections that contain data pertaining to particular analysis subjects. Items in each section are independently numbered. The sections are as follows: **SM-SYN** (synthetic and analytical details for compounds **1**–**24**, 24 Figures a,b,c), **SM-INH** (dose-response curves for **3**, **4 and 5**, 3 Figures) **SM-COR** (input and results of correlational analysis, 1 Table and 1 Listing), **SM-SIM** (results of molecular dynamics simulations, 6 Figures and 1 table), **SM-RES** (TIC chromatograms and mass spectra 13 Figures, data sheet for linear graph 2 Tables), **SM-SUR** (datasheet from MUSE Cell Analyser, 1 Table).

## References

[1] Fong G.-H., Rossant J., Gertsenstein M., Breitman M.L. (1995). Role of the Flt-1 receptor tyrosine kinase in regulating the assembly of vascular endothelium. Nature.

[2] Shay S., Hua-Quan M., Masashi N., Seiji T., Michael K. (2002). VEGF165 mediates formation of complexes containing VEGFR-2 and neuropilin-1 that enhance VEGF165-receptor binding. J. Cell. Biochem..

[3] Fuh G. (2000). The interaction of Neuropilin-1 with Vascular Endothelial Growth Factor and its receptor Flt-1. J. Biol. Chem..

[4] Tordjman R., Lepelletier Y., Lemarchandel V., Cambot M., Gaulard P., Hermine O., Roméo P.-H. (2002). A neuronal receptor, neuropilin-1, is essential for the initiation of the primary immune response. Nat. Immunol..

[5] Bruder D., Probst-Kepper M., Westendorf A.M., Geffers R., Beissert S., Loser K., von Boehmer H., Buer J., Hansen W. (2004). Frontline: Neuropilin-1: A surface marker of regulatory T cells. Eur. J. Immunol..

[6] Mizui M., Kikutani H. (2008). Neuropilin-1: The Glue between Regulatory T Cells and Dendritic Cells?. Immunity.

[7] Jubb A.M., Strickland L.A., Liu S.D., Mak J., Schmidt M., Koeppen H., Jubb M. A., Strickland A.L., Liu S.D., Mak J. (2012). Neuropilin-1 expression in cancer and development. J. Pathol..

[8] Soker S., Takashima S., Miao H.Q., Neufeld G., Klagsbrun M. (1998). Neuropilin-1 Is Expressed by Endothelial and Tumor Cells as an Isoform-Specific Receptor for Vascular Endothelial Growth Factor. Cell.

[9] Soker S., Fidder H., Neufeld G., Klagsbrun M. (1996). Characterization of novel vascular endothelial growth factor (VEGF) receptors on tumor cells that bind VEGF165 via its exon 7-encoded domain. J. Biol. Chem..

[10] Goel H.L., Mercurio A.M. (2013). VEGF targets the tumour cell. Nat. Rev. Cancer.

[11] Bachelder R.E., Crago A., Chung J., Wendt M.A., Shaw L.M., Robinson G., Mercurio A.M. (2001). Vascular Endothelial Growth Factor Is an Autocrine Survival Factor for Neuropilin-expressing Breast Carcinoma Cells. Cancer Res..

[12] Hansen W. (2013). Neuropilin 1 guides regulatory T cells into vegf-producing melanoma. Oncoimmunology.

[13] Djordjevic S., Driscoll P.C. (2013). Targeting VEGF signalling via the neuropilin co-receptor. Drug Discov. Today.

[14] Peng K., Bai Y., Zhu Q., Hu B., Xu Y. (2019). Targeting VEGF–neuropilin interactions: A promising antitumor strategy. Drug Discov. Today.

[15] Niland S., Eble J.A. (2019). Neuropilins in the Context of Tumor Vasculature. Int. J. Mol. Sci..

[16] Powell J., Mota F., Steadman D., Soudy C., Miyauchi J.T., Crosby S., Jarvis A., Reisinger T., Winfield N., Evans G. (2018). Small Molecule Neuropilin-1 Antagonists Combine Antiangiogenic and Antitumor Activity with Immune Modulation through Reduction of Transforming Growth Factor Beta (TGFβ) Production in Regulatory T-Cells. J. Med. Chem..

[17] Novoa A., Pellegrini-Moïse N., Bechet D., Barberi-Heyob M., Chapleur Y. (2010). Sugar-based peptidomimetics as potential inhibitors of the vascular endothelium growth factor binding to neuropilin-1. Bioorg. Med. Chem..

[18] Liu W.-Q., Lepelletier Y., Montès M., Borriello L., Jarray R., Grépin R., Leforban B., Loukaci A., Benhida R., Hermine O. (2018). NRPa-308, a new neuropilin-1 antagonist, exerts in vitro anti-angiogenic and anti-proliferative effects and in vivo anti-cancer effects in a mouse xenograft model. Cancer Lett..

[19] Borriello L., Montès M., Lepelletier Y., Leforban B., Liu W.-Q.Q., Demange L., Delhomme B., Pavoni S., Jarray R., Boucher J.L. (2014). Structure-based discovery of a small non-peptidic Neuropilins antagonist exerting in vitro and in vivo anti-tumor activity on breast cancer model. Cancer Lett..

[20] Starzec A., Miteva M.A., Ladam P., Villoutreix B.O., Perret G.Y. (2014). Discovery of novel inhibitors of vascular endothelial growth factor-A-Neuropilin-1 interaction by structure-based virtual screening. Bioorg. Med. Chem..

[21] Jarvis A., Allerston C.K., Jia H., Herzog B., Garza-Garcia A., Winfield N., Ellard K., Aqil R., Lynch R., Chapman C. (2010). Small Molecule Inhibitors of the Neuropilin-1 Vascular Endothelial Growth Factor A (VEGF-A) Interaction. J. Med. Chem..

[22] Soker S., Gollamudi-Payne S., Fidder H., Charmahelli H., Klagsbrun M. (1997). Inhibition of Vascular Endothelial Growth Factor (VEGF)-induced Endothelial Cell Proliferation by a Peptide Corresponding to the Exon 7-Encoded Domain of VEGF165. J. Biol. Chem..

[23] Jia H., Bagherzadeh A., Hartzoulakis B., Jarvis A., Löhr M., Shaikh S., Aqil R., Cheng L., Tickner M., Esposito D. (2006). Characterization of a bicyclic peptide neuropilin-1 (NP-1) antagonist (EG3287) reveals importance of vascular endothelial growth factor exon 8 for NP-1 binding and role of NP-1 in KDR signaling. J. Biol. Chem..

[24] Grabowska K., Puszko A.K., Lipiński P.F.J., Laskowska A.K., Wileńska B., Witkowska E., Misicka A. (2016). Design, synthesis and in vitro biological evaluation of a small cyclic peptide as inhibitor of vascular endothelial growth factor binding to neuropilin-1. Bioorg. Med. Chem. Lett..

[25] Getz J.A., Cheneval O., Craik D.J., Daugherty P.S. (2013). Design of a Cyclotide Antagonist of Neuropilin-1 and -2 That Potently Inhibits Endothelial Cell Migration. ACS Chem. Biol..

[26] Grabowska K., Puszko A.K., Lipiński P.F.J., Laskowska A.K., Wileńska B., Witkowska E., Perret G.Y., Misicka A. (2016). Structure-activity relationship study of a small cyclic peptide H-c[Lys-Pro-Glu]-Arg-OH: A potent inhibitor of Vascular Endothelial Growth Factor interaction with Neuropilin-1. Bioorg. Med. Chem..

[27] Binétruy-Tournaire R., Demangel C., Malavaud B., Vassy R., Rouyre S., Kraemer M., Plouët J., Derbin C., Perret G., Mazié J.C. (2000). Identification of a peptide blocking vascular endothelial growth factor (VEGF)-mediated angiogenesis. EMBO J..

[28] Fedorczyk B., Lipiński P.F.J., Tymecka D., Puszko A.K., Wilenska B., Perret G.Y., Misicka A. (2017). Conformational latitude—Activity relationship of KPPR tetrapeptide analogues toward their ability to inhibit binding of Vascular Endothelial Growth Factor 165 to Neuropilin-1. J. Pept. Sci..

[29] Tymecka D., Puszko A.K., Lipiński P.F.J., Fedorczyk B., Wilenska B., Sura K., Perret G.Y., Misicka A. (2018). Branched pentapeptides as potent inhibitors of the vascular endothelial growth factor 165 binding to Neuropilin-1: Design, synthesis and biological activity. Eur. J. Med. Chem..

[30] Tymecka D., Lipiński P.F.J.P.F.J., Fedorczyk B., Puszko A., Wileńska B., Perret G.Y.G.Y., Misicka A., Lipi P.F.J., Puszko A., Wile B. (2017). Structure-activity relationship study of tetrapeptide inhibitors of the Vascular Endothelial Growth Factor A binding to Neuropilin-1. Peptides.

[31] Starzec A., Vassy R., Martin A., Lecouvey M., Di Benedetto M., Crépin M., Perret G.Y. (2006). Antiangiogenic and antitumor activities of peptide inhibiting the vascular endothelial growth factor binding to neuropilin-1. Life Sci..

[32] Puszko A.K., Sosnowski P., Tymecka D., Raynaud F., Hermine O., Lepelletier Y., Misicka A. (2019). Neuropilin-1 peptide-like ligands with proline mimetics, tested using the improved chemiluminescence affinity detection method. Medchemcomm.

[33] Tron G.C., Pirali T., Billington R.A., Canonico P.L., Sorba G., Genazzani A.A. (2008). Click chemistry reactions in medicinal chemistry: Applications of the 1,3-dipolar cycloaddition between azides and alkynes. Med. Res. Rev..

[34] Diness F., Schoffelen S., Meldal M., Lubell W.D. (2017). Advances in Merging Triazoles with Peptides and Proteins. Peptidomimetics I..

[35] Tornøe C.W., Christensen C., Meldal M. (2002). Peptidotriazoles on solid phase: [1,2,3]-Triazoles by regiospecific copper(I)-catalyzed 1,3-dipolar cycloadditions of terminal alkynes to azides. J. Org. Chem..

[36] Rostovtsev V.V., Green L.G., Fokin V.V., Sharpless K.B. (2002). A Stepwise Huisgen Cycloaddition Process: Copper(I)-Catalyzed Regioselective “Ligation” of Azides and Terminal Alkynes. Angew. Chemie Int. Ed..

[37] Ben Haj Salah K., Das S., Ruiz N., Andreu V., Martinez J., Wenger E., Amblard M., Didierjean C., Legrand B., Inguimbert N. (2018). How are 1,2,3-triazoles accommodated in helical secondary structures?. Org. Biomol. Chem..

[38] Nyffeler P.T., Liang C.H., Koeller K.M., Wong C.H. (2002). The chemistry of amine-azide interconversion: Catalytic diazotransfer and regioselective azide reduction. J. Am. Chem. Soc..

[39] Lundquist IV J.T., Pelletier J.C. (2001). Improved solid-phase peptide synthesis method utilizing α-azide-protected amino acids. Org. Lett..

[40] Punna S., Finn M.G. (2004). A Convenient Colorimetric Test for Aliphatic Azides. Synlett.

[41] Bernatowicz M.S., Youling W., Matsueda G.R. (1992). 1H-Pyrazole-1-carboxamidine Hydrochloride: An Attractive Reagent for Guanylation of Amines and Its Application to Peptide Synthesis. J. Org. Chem..

[42] Starzec A., Ladam P., Vassy R., Badache S., Bouchemal N., Navaza A., du Penhoat C.H., Perret G.Y. (2007). Structure-function analysis of the antiangiogenic ATWLPPR peptide inhibiting VEGF165 binding to neuropilin-1 and molecular dynamics simulations of the ATWLPPR/neuropilin-1 complex. Peptides.

[43] Vander Kooi C.W., Jusino M.A., Perman B., Neau D.B., Bellamy H.D., Leahy D.J. (2007). Structural basis for ligand and heparin binding to neuropilin B domains. Proc. Natl. Acad. Sci. USA.

[44] Parker M.W., Xu P., Li X., Vander Kooi C.W. (2012). Structural Basis for Selective Vascular Endothelial Growth Factor-A (VEGF-A) Binding to Neuropilin-1. J. Biol. Chem..

[45] Mota F., Fotinou C., Rhana R., Chan A.W.E., Yelland T., Arooz M.T., O’Leary A.P., Hutton J., Frankel P., Zachary I. (2018). Architecture and hydration of the arginine-binding site of neuropilin-1. FEBS J..

[46] Case D.A., Babin V., Berryman J.T., Betz R.M., Cai Q., Cerutti D.S., Cheatham T.E.I., Darden T.A., Duke R.E., Gohlke H. (2014). Amber14.

[47] Maier J.A., Martinez C., Kasavajhala K., Wickstrom L., Hauser K.E., Simmerling C. (2015). ff14SB: Improving the Accuracy of Protein Side Chain and Backbone Parameters from ff99SB. J. Chem. Theory Comput..

[48] Marion A., Góra J., Kracker O., Fröhr T., Latajka R., Sewald N., Antes I. (2018). Amber-Compatible Parametrization Procedure for Peptide-like Compounds: Application to 1,4- and 1,5-Substituted Triazole-Based Peptidomimetics. J. Chem. Inf. Model..

[49] Michaud-Agrawal N., Denning E.J., Woolf T.B., Beckstein O. (2011). MDAnalysis: A toolkit for the analysis of molecular dynamics simulations. J. Comput. Chem..

[50] Pettersen E.F., Goddard T.D., Huang C.C., Couch G.S., Greenblatt D.M., Meng E.C., Ferrin T.E. (2004). UCSF Chimera—A Visualization System for Exploratory Research and Analysis. J. Comput. Chem..

