# Supplementary material for: Triazolopeptides Inhibiting the Interaction between Neuropilin-1 and Vascular Endothelial Growth Factor-165
Source: Molecules. 2019 May 6;24(9):1756. doi: 10.3390/molecules24091756 (PMC6539594; doi:10.3390/molecules24091756)
Supplement: Supplementary file 1 [file molecules-24-01756-s001.pdf]

Supplementary Materials to:

Triazolopeptides inhibiting the interaction between Neuropilin-1 and Vascular Endothelial Growth Factor-165

by

Bartlomiej Fedorczyk*, Piotr F.J. Lipinski, Anna K. Puszko, Dagmara Tymecka, Beata Wilenska, Wioleta Dudka, Gerard Y. Perret, Rafal Wieczorek and Aleksandra Misicka*

***Corresponding authors:** Aleksandra Misicka, misicka@chem.uw.edu.pl, Bartlomiej Fedorczyk, bfedorczyk@chem.uw.edu.pl

Table of contents

SM-SYN. Synthetic data 2

SM-INH. Inhibitory activity 27

SM-COR. Correlational analysis 28

SM-SIM. Simulations 30

SM-RES. Proteolytic resistance 40

SM-SUR. Preliminary cell survival test 46

SM-SYN. Synthetic data

Compound 1

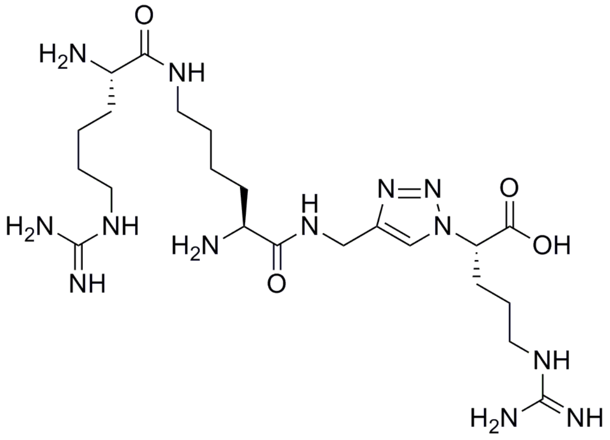

Figure SM-SYN-1a. Structure of compound **1** Lys(Har)-GlyΨ[Trl]Arg.

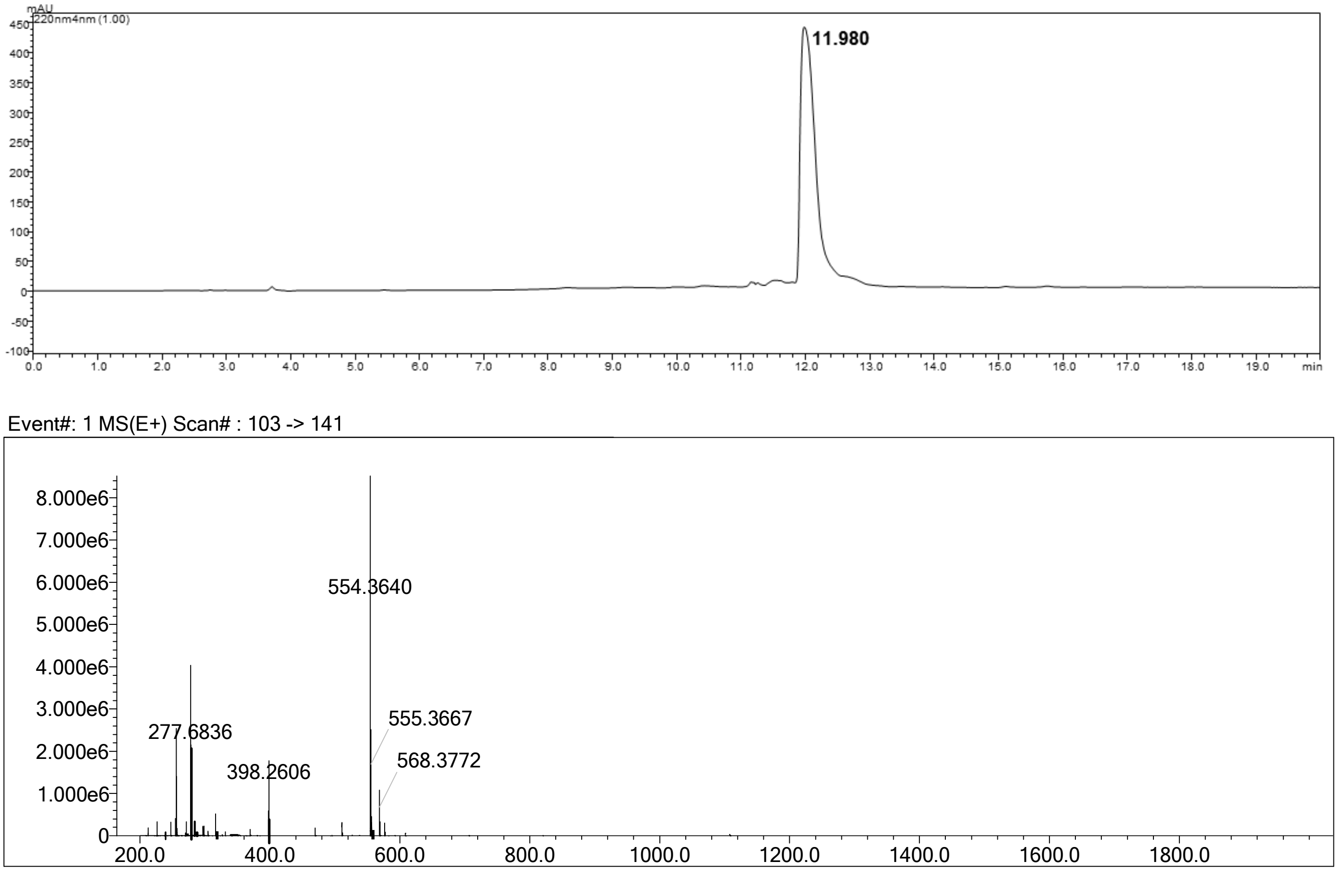

Rank	Score	Formula (M)	Ion	Meas. m/z	Pred. m/z	Df. (mDa)	Df. (ppm)	Iso	DBE
1	99.80	C22 H43 N13 O4	[M+H]+	554.3640	554.3634	0.6	1.08	100.00	8.0

Figure SM-SYN-1b. HPLC chromatogram of compound 1 at 220 nm and MS spectrum

Compound 2

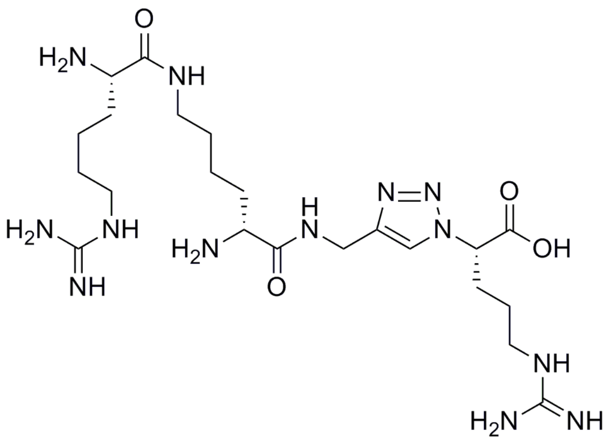

Figure SM-SYN-2a. Structure of compound **2** D-Lys(Har)-GlyΨ[Trl]Arg.

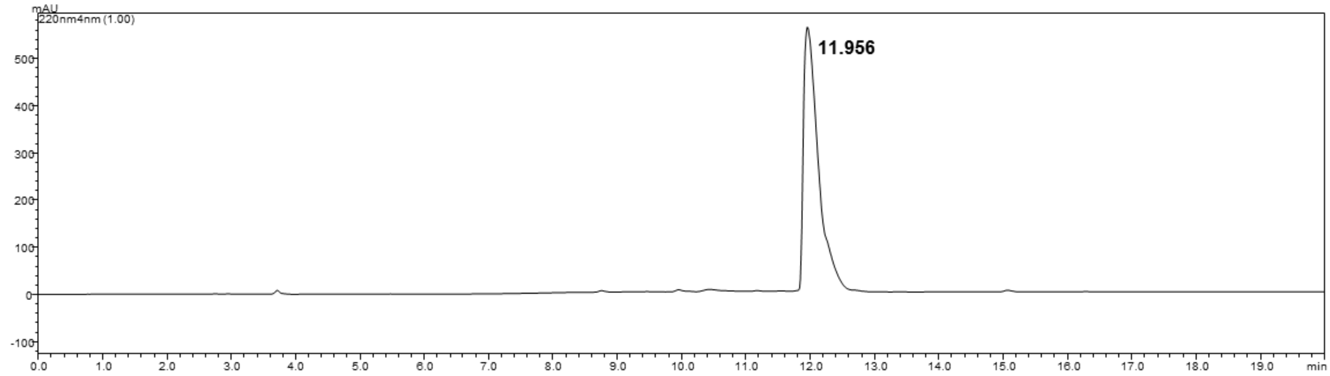

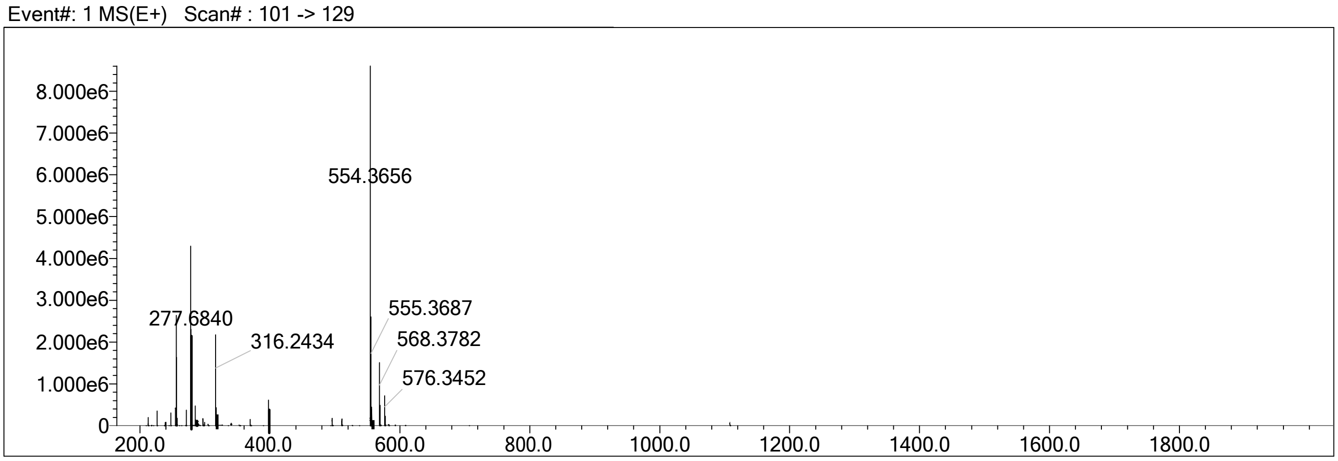

Rank	Score	Formula (M)	Ion	Meas. m/z	Pred. m/z	Df. (mDa)	Df. (ppm)	Iso	DBE
4	92.58	C22 H43 N13 O4	[M+H]+	554.3656	554.3634	2.2	3.97	100.00	8.0

Figure SM-SYN-2b. HPLC chromatogram of compound **2** at 220 nm and MS spectrum

Compound 3

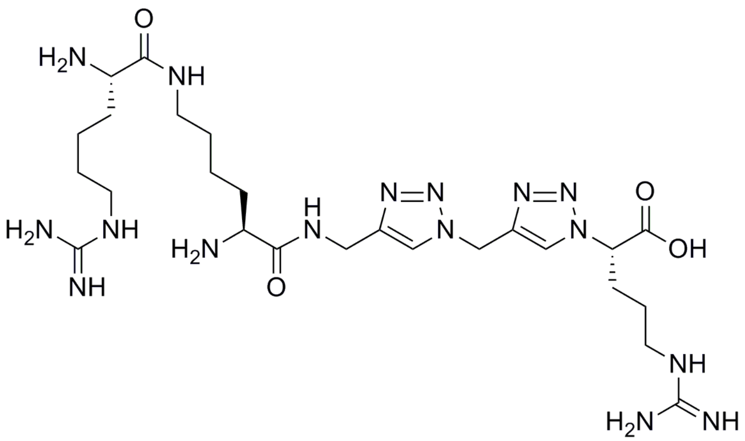

Figure SM-SYN-3a. Structure of compound **3** Lys(Har)-GlyΨ[Trl]GlyΨ[Trl]Arg.

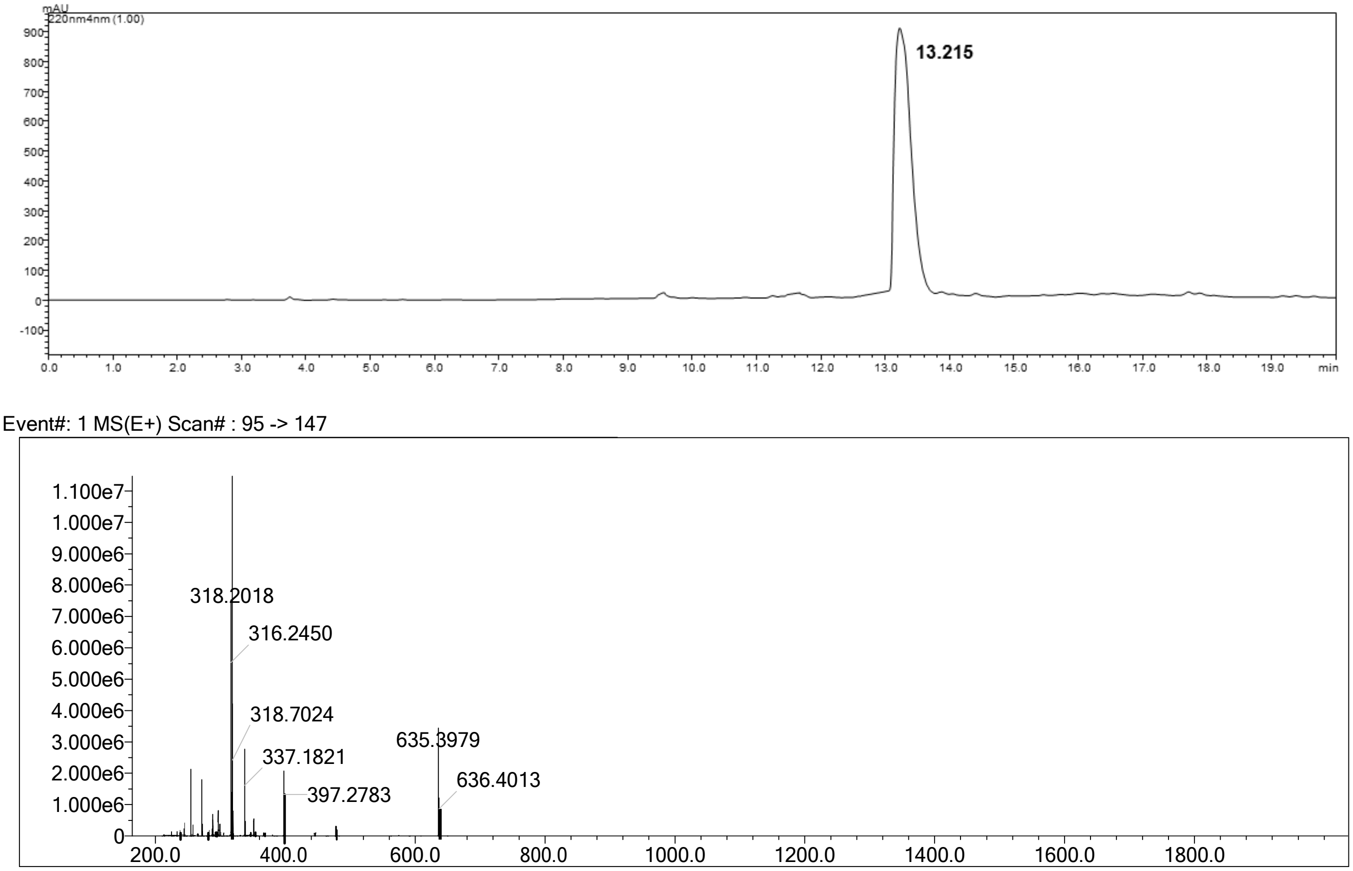

Rank	Score	Formula (M)	Ion	Meas. m/z	Pred. m/z	Df. (mDa)	Df. (ppm)	Iso	DBE
4	95.42	C25 H46 N16 O4	[M+H]+	635.3979	635.3961	1.8	2.83	100.00	11.0

Figure SM-SYN-3b. HPLC chromatogram of compound **3** at 220 nm and MS spectrum

Compound 4

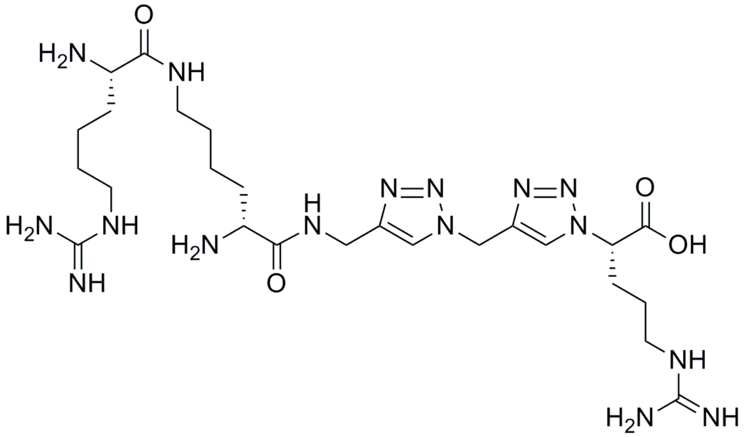

Figure SM-SYN-4a. Structure of compound **4** D-Lys(Har)-GlyΨ[Trl]GlyΨ[Trl]Arg.

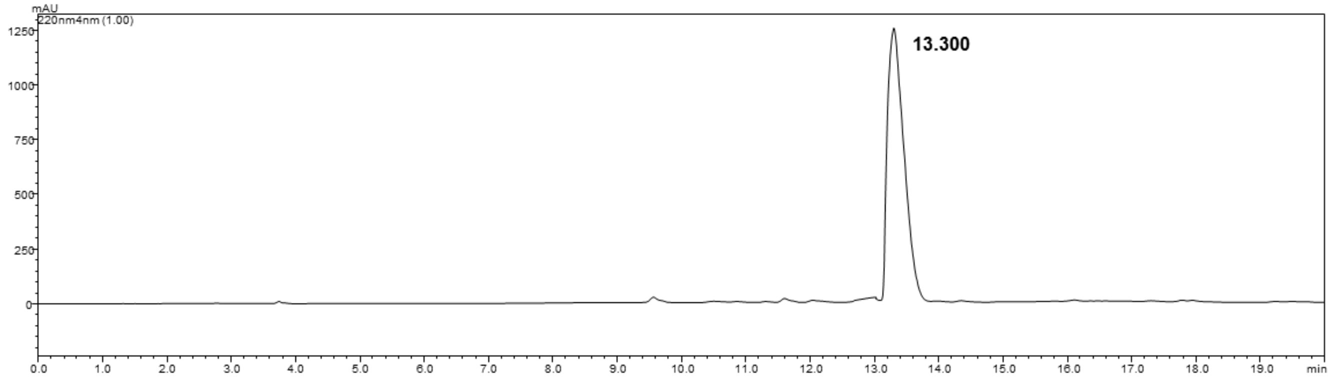

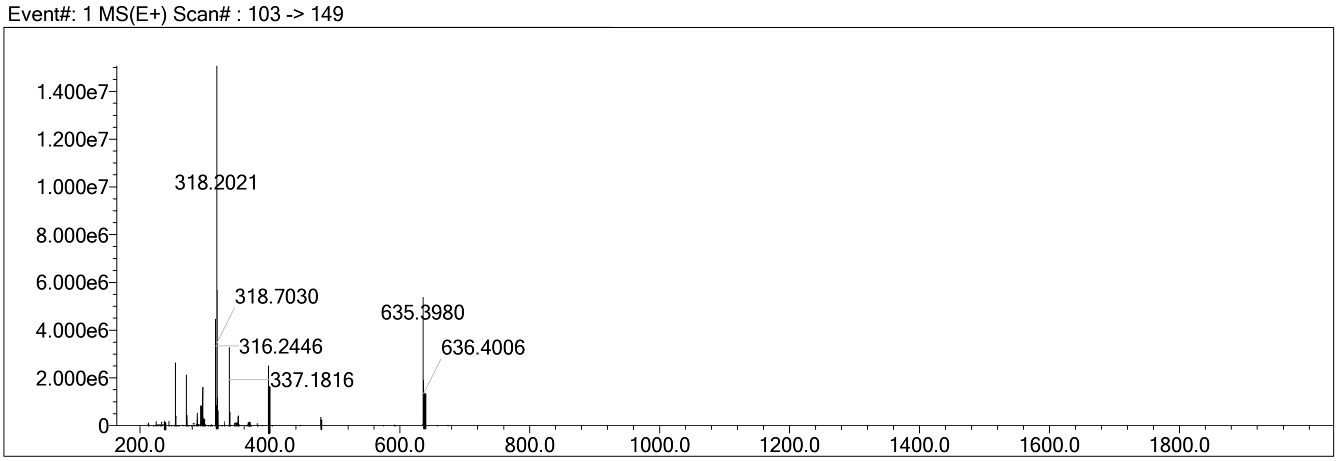

Rank	Score	Formula (M)	Ion	Meas. m/z	Pred. m/z	Df. (mDa)	Df. (ppm)	Iso	DBE
4	95.03	C25 H46 N16 O4	[M+H]+	635.3980	635.3961	1.9	2.99	100.00	11.0

Figure SM-SYN-4b. HPLC chromatogram of compound **4** at 220 nm and MS spectrum

Compound 5

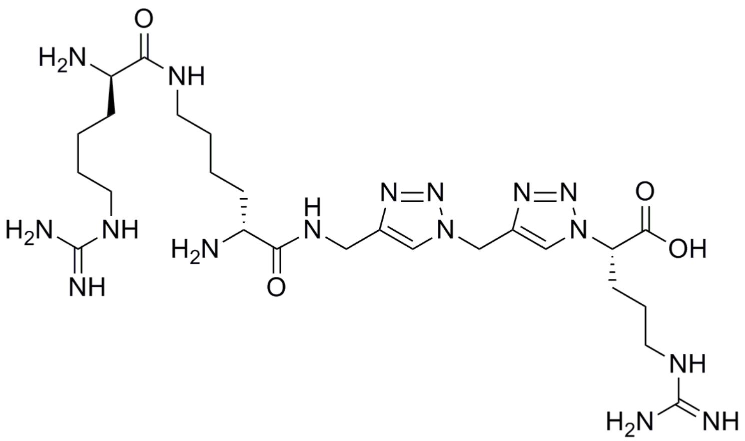

Figure SM-SYN-5a. Structure of compound **5** D-Lys(D-Har)-GlyΨ[Trl]GlyΨ[Trl]Arg.

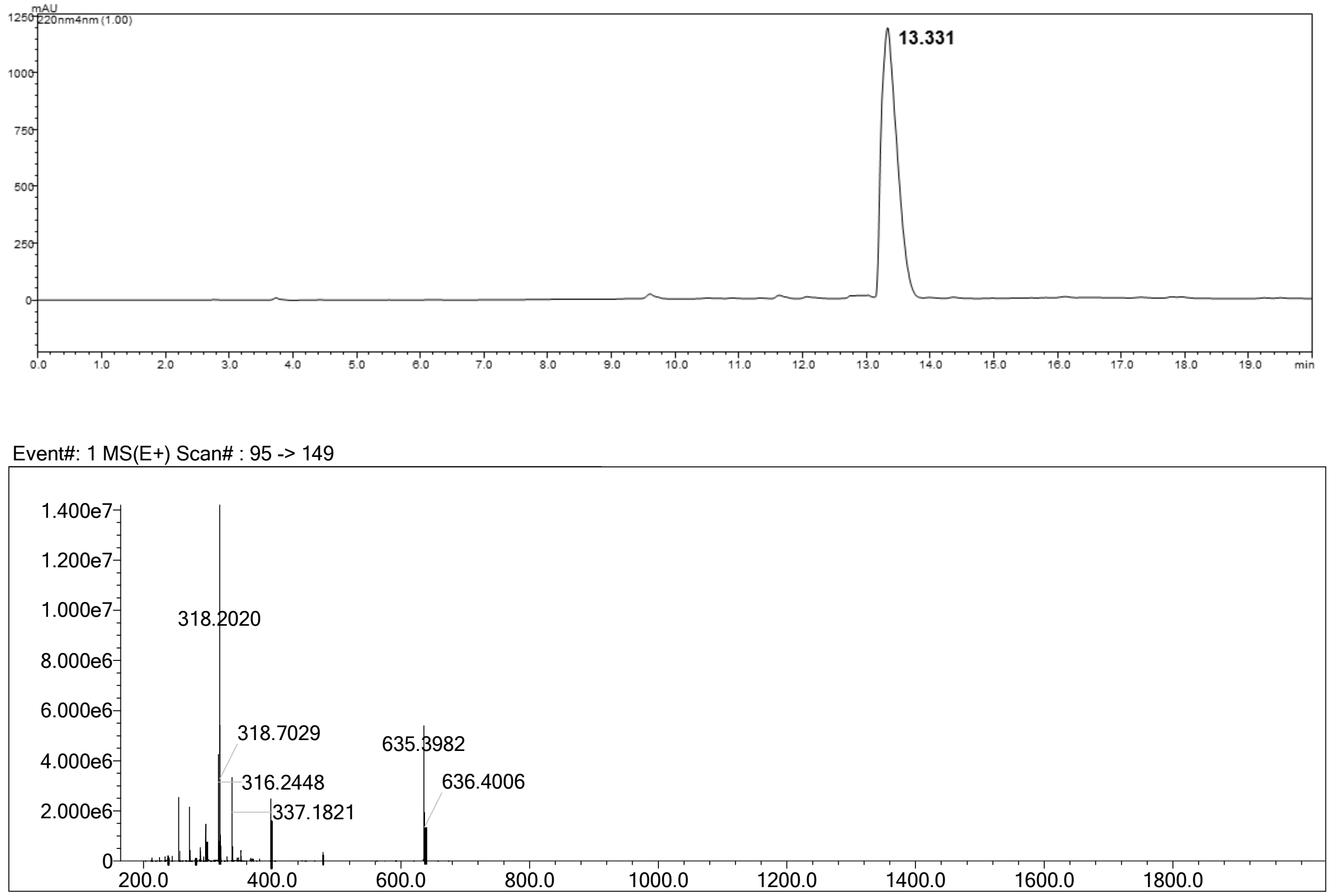

Rank	Score	Formula (M)	Ion	Meas. m/z	Pred. m/z	Df. (mDa)	Df. (ppm)	Iso	DBE
4	94.22	C25 H46 N16 O4	[M+H]+	635.3982	635.3961	2.1	3.31	100.00	11.0

Figure SM-SYN-5b. HPLC chromatogram of compound **5** at 220 nm and MS spectrum

Compound 6

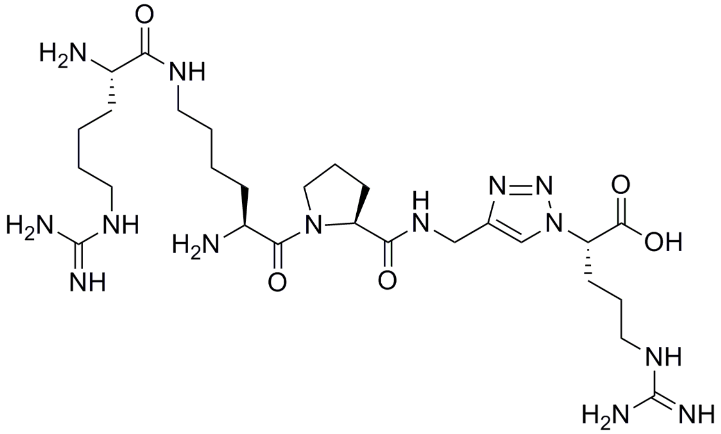

Figure SM-SYN-6a. Structure of compound **6** Lys(Har)-Pro-GlyΨ[Trl]Arg.

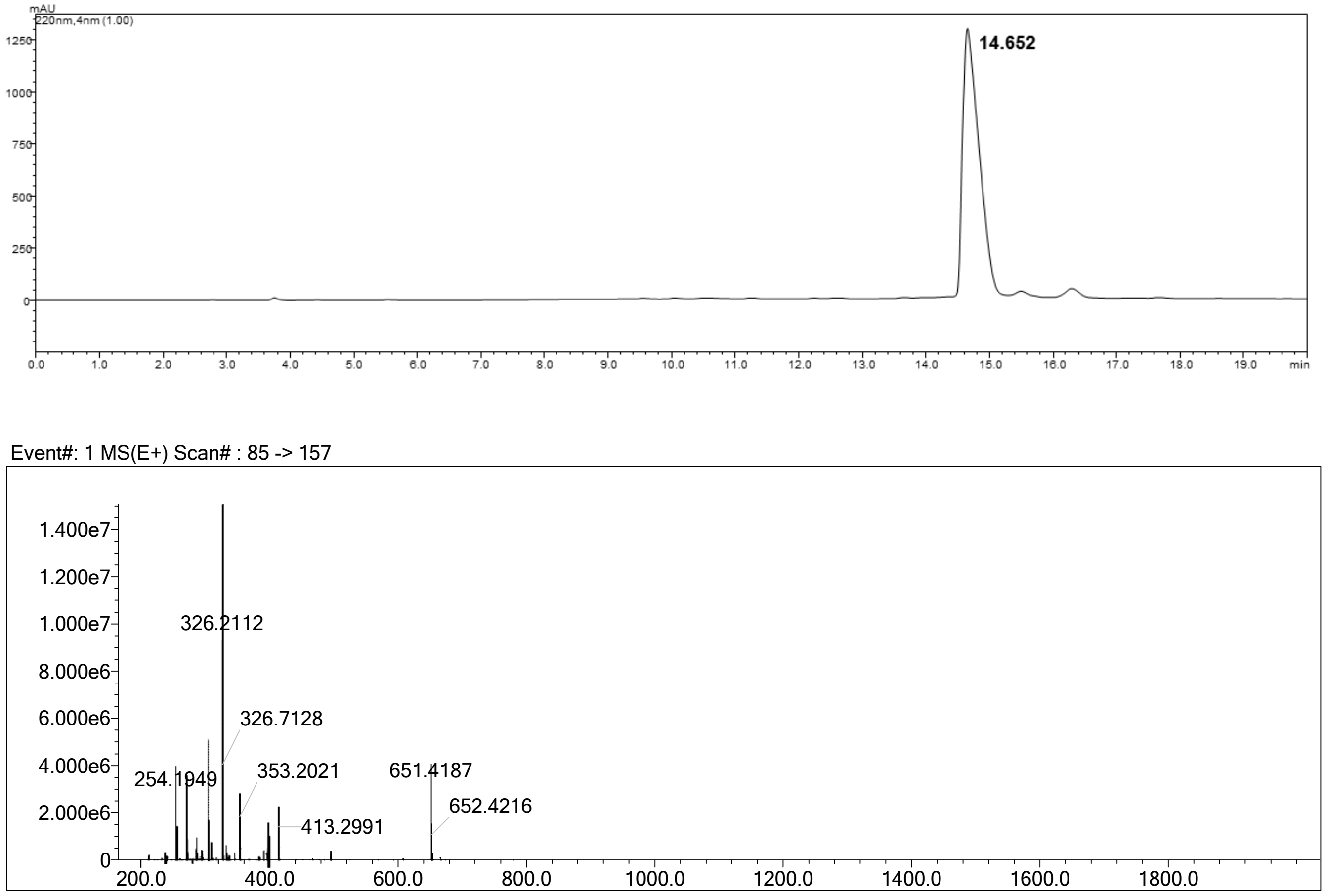

Rank	Score	Formula (M)	Ion	Meas. m/z	Pred. m/z	Df. (mDa)	Df. (ppm)	Iso	DBE
4	92.53	C27 H50 N14 O5	[M+H]+	651.4187	651.4161	2.6	3.99	100.00	10.0

Figure SM-SYN-6b. HPLC chromatogram of compound **6** at 220 nm and MS spectrum

Compound 7

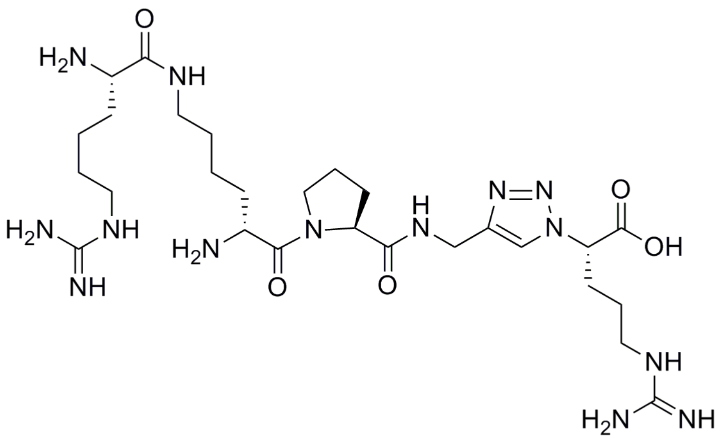

Figure SM-SYN-7a. Structure of compound **7** D-Lys(Har)-Pro-GlyΨ[Trl]Arg.

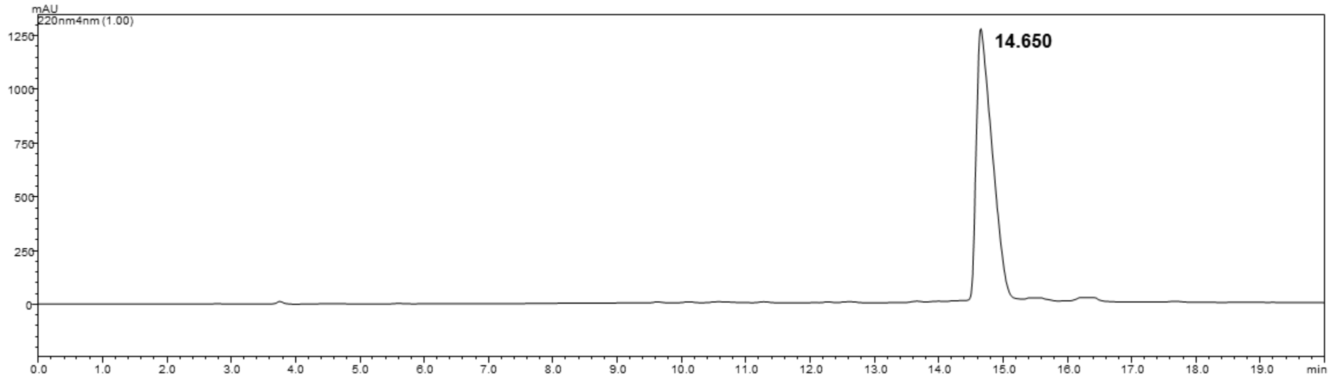

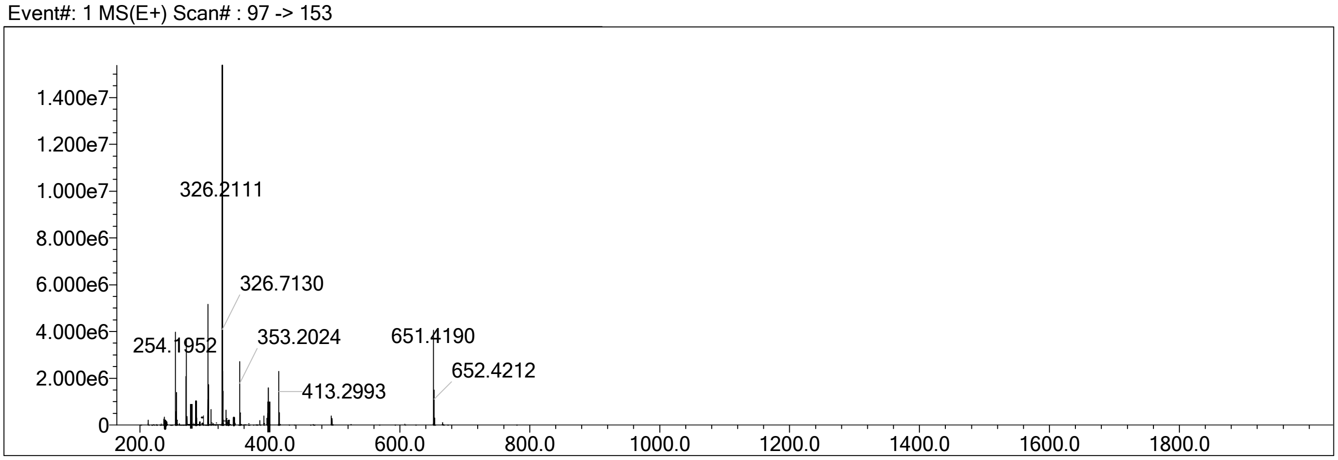

Rank	Score	Formula (M)	Ion	Meas. m/z	Pred. m/z	Df. (mDa)	Df. (ppm)	Iso	DBE
4	91.38	C27 H50 N14 O5	[M+H]+	651.4190	651.4161	2.9	4.45	100.00	10.0

Figure SM-SYN-7b. HPLC chromatogram of compound **7** at 220 nm and MS spectrum

Compound 8

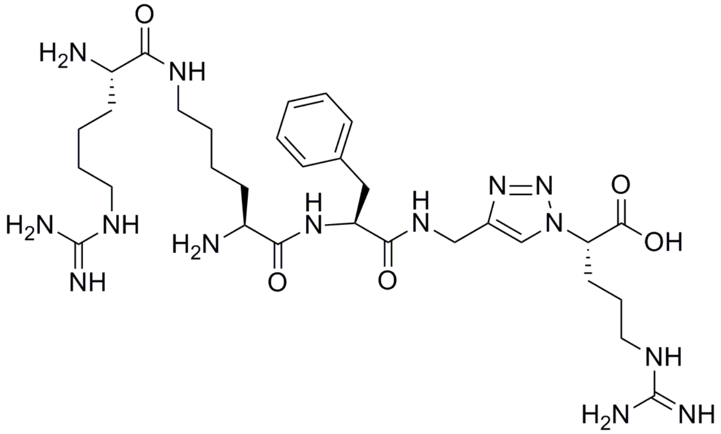

Figure SM-SYN-8a. Structure of compound **8** Lys(Har)-Phe-GlyΨ[Trl]Arg.

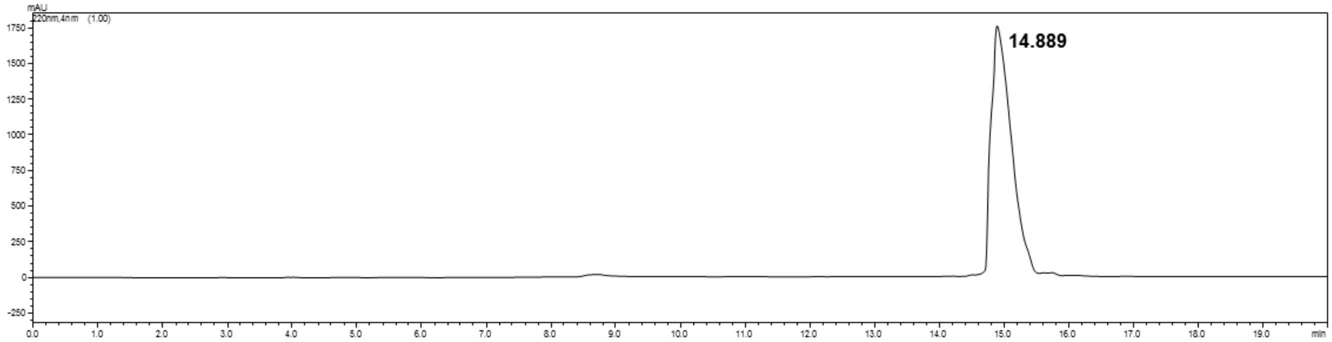

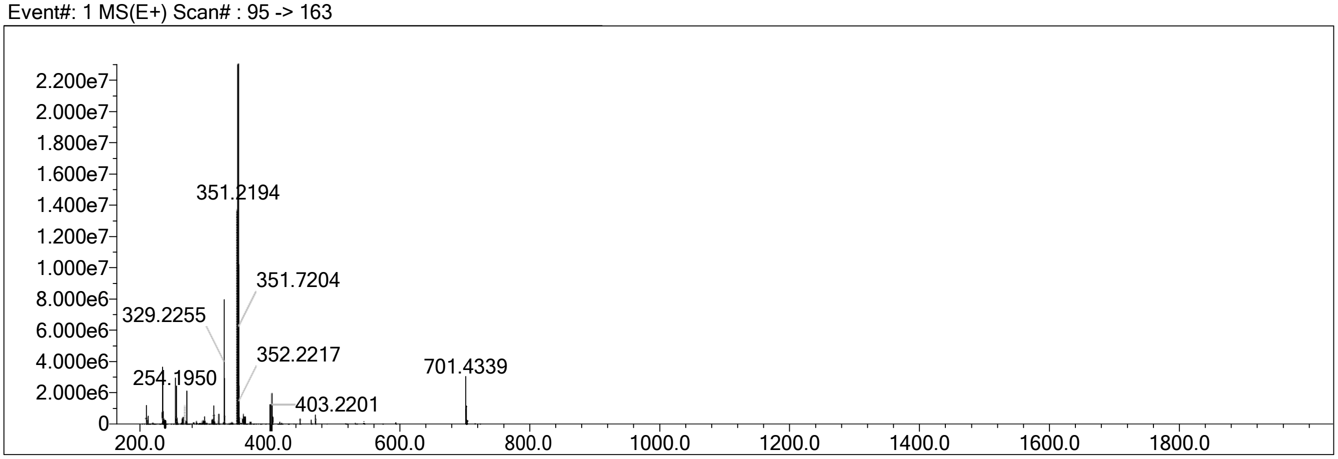

Rank	Score	Formula (M)	Ion	Meas. m/z	Pred. m/z	Df. (mDa)	Df. (ppm)	Iso	DBE
1	95.03	C31 H52 N14 O5	[M+H]+	701.4339	701.4318	2.1	2.99	100.00	13.0

Figure SM-SYN-8b. HPLC chromatogram of compound **8** at 220 nm and MS spectrum

Compound 9

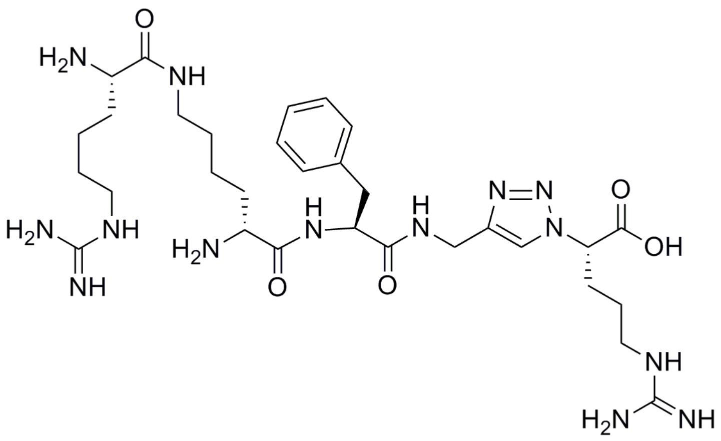

Figure SM-SYN-9a. Structure of compound **9** D-Lys(Har)-Phe-GlyΨ[Trl]Arg.

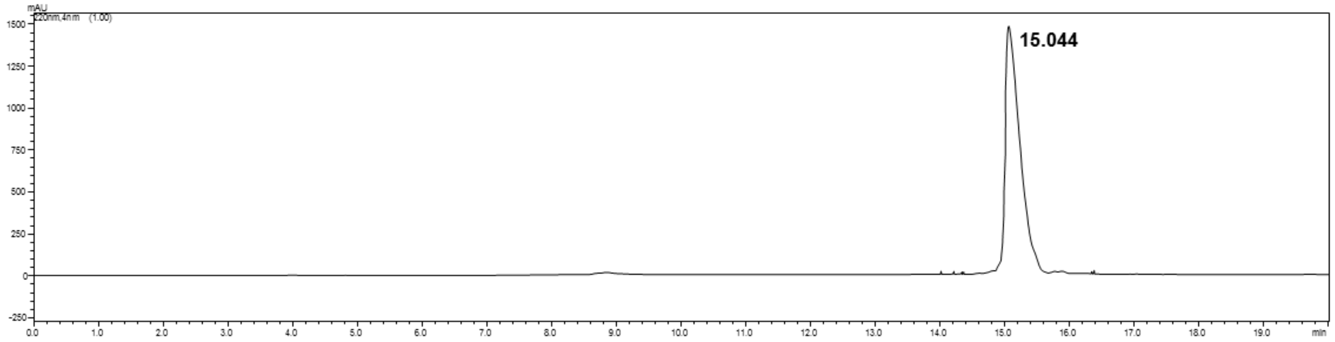

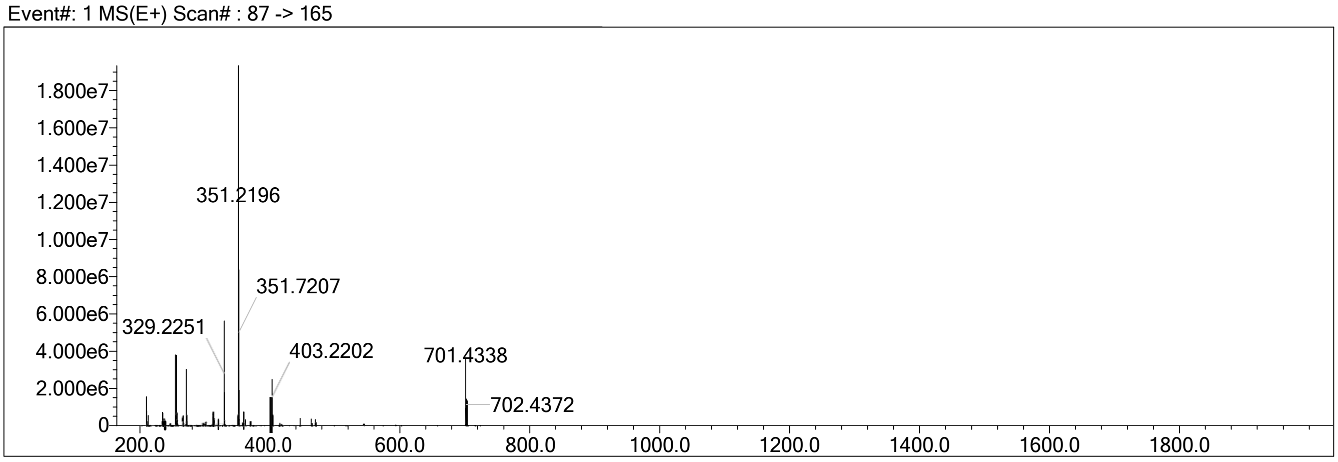

Rank	Score	Formula (M)	Ion	Meas. m/z	Pred. m/z	Df. (mDa)	Df. (ppm)	Iso	DBE
2	94.20	C31 H52 N14 O5	[M+H]+	701.4338	701.4318	2.0	2.85	98.77	13.0

Figure SM-SYN-9b. HPLC chromatogram of compound **9** at 220 nm and MS spectrum

Compound 10

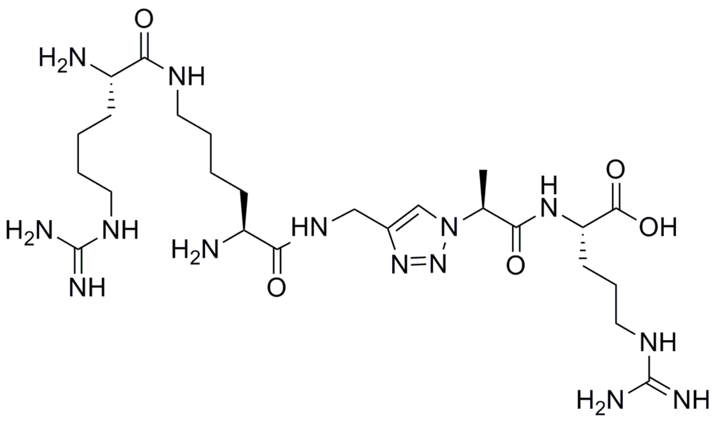

Figure SM-SYN-10a. Structure of compound **10** Lys(Har)-GlyΨ[Trl]Ala-Arg.

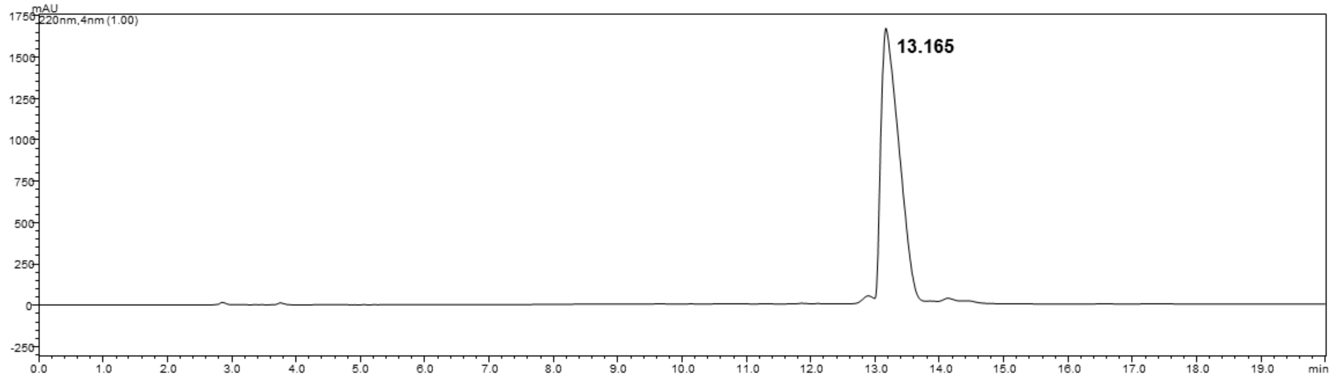

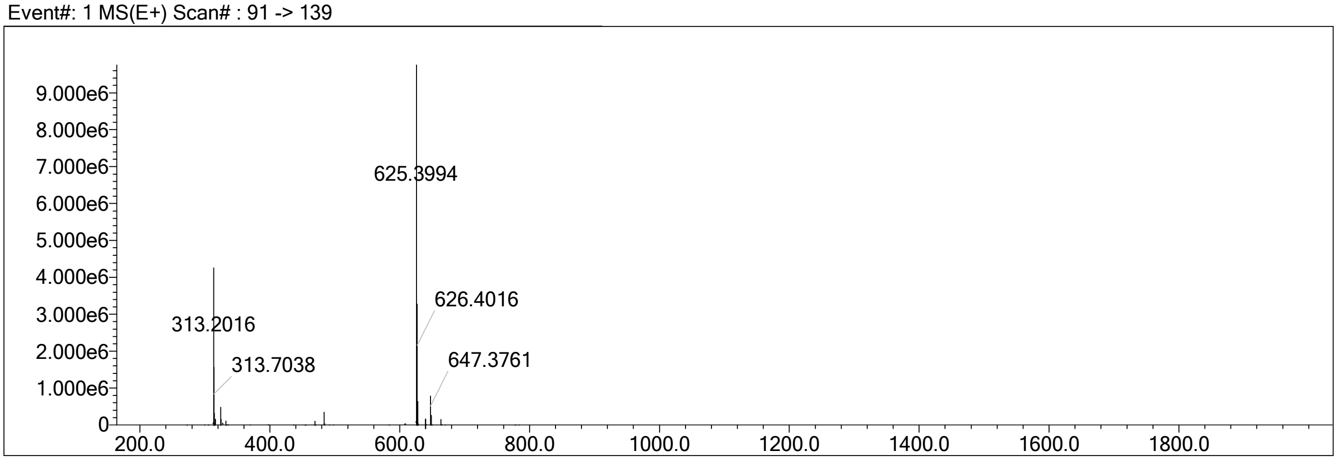

Rank	Score	Formula (M)	Ion	Meas. m/z	Pred. m/z	Df. (mDa)	Df. (ppm)	Iso	DBE
2	98.10	C25 H48 N14 O5	[M+H]+	625.3994	625.4005	-1.1	-1.76	100.00	9.0

Figure SM-SYN-10b. HPLC chromatogram of compound **10** at 220 nm and MS spectrum

Compound 11

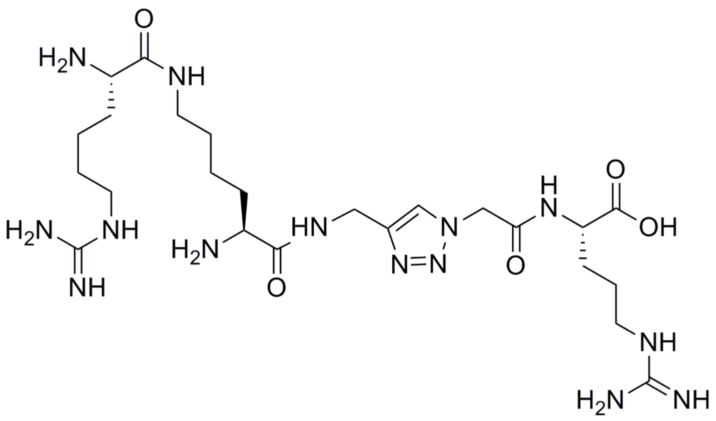

Figure SM-SYN-11a. Structure of compound **11** Lys(Har)-GlyΨ[Trl]Gly-Arg.

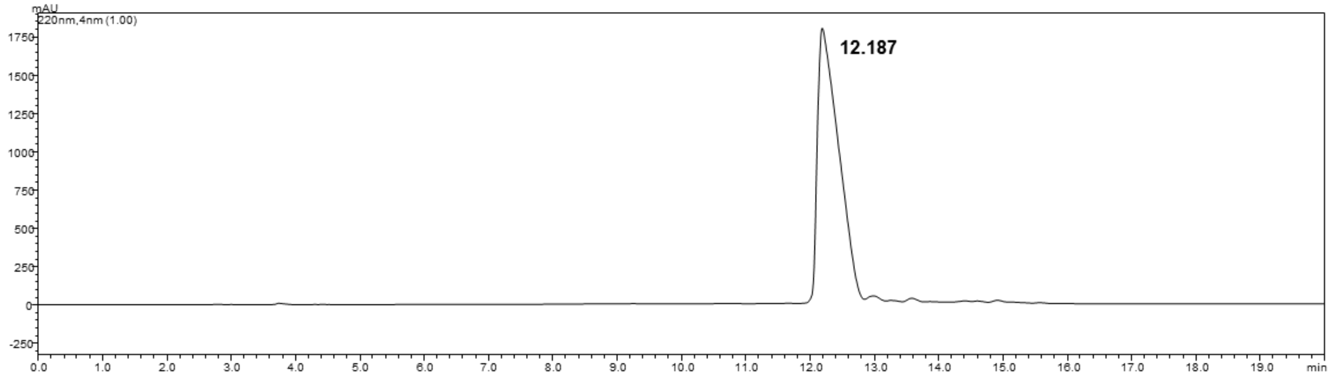

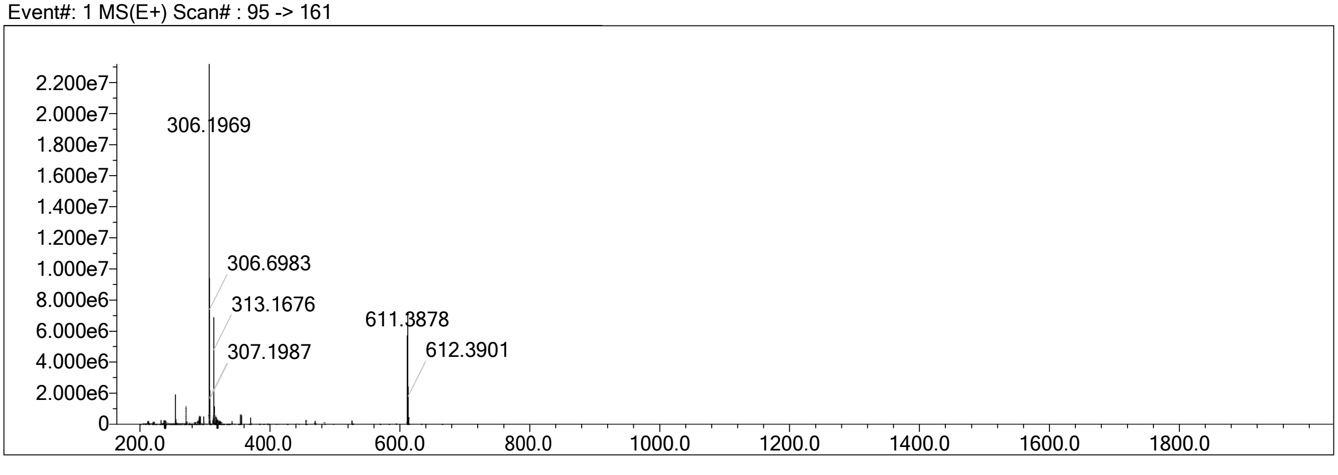

Rank	Score	Formula (M)	Ion	Meas. m/z	Pred. m/z	Df. (mDa)	Df. (ppm)	Iso	DBE
4	90.22	C24 H46 N14 O5	[M+H]+	611.3878	611.3848	3.0	4.91	100.00	9.0

Figure SM-SYN-11b. HPLC chromatogram of compound **11** at 220 nm and MS spectrum

Compound 12

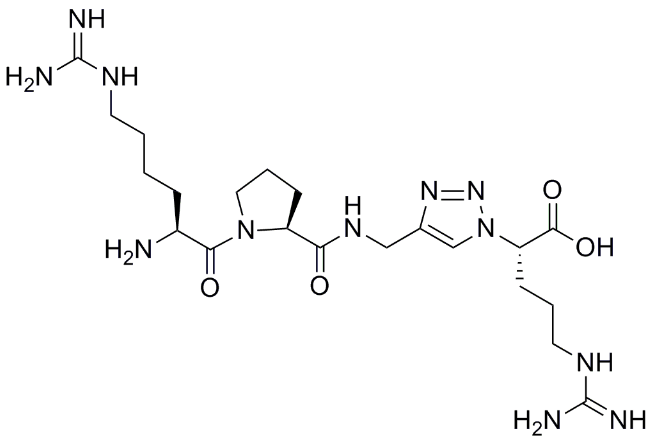

Figure SM-SYN-12a. Structure of compound **12** Har-Pro-GlyΨ[Trl]Arg.

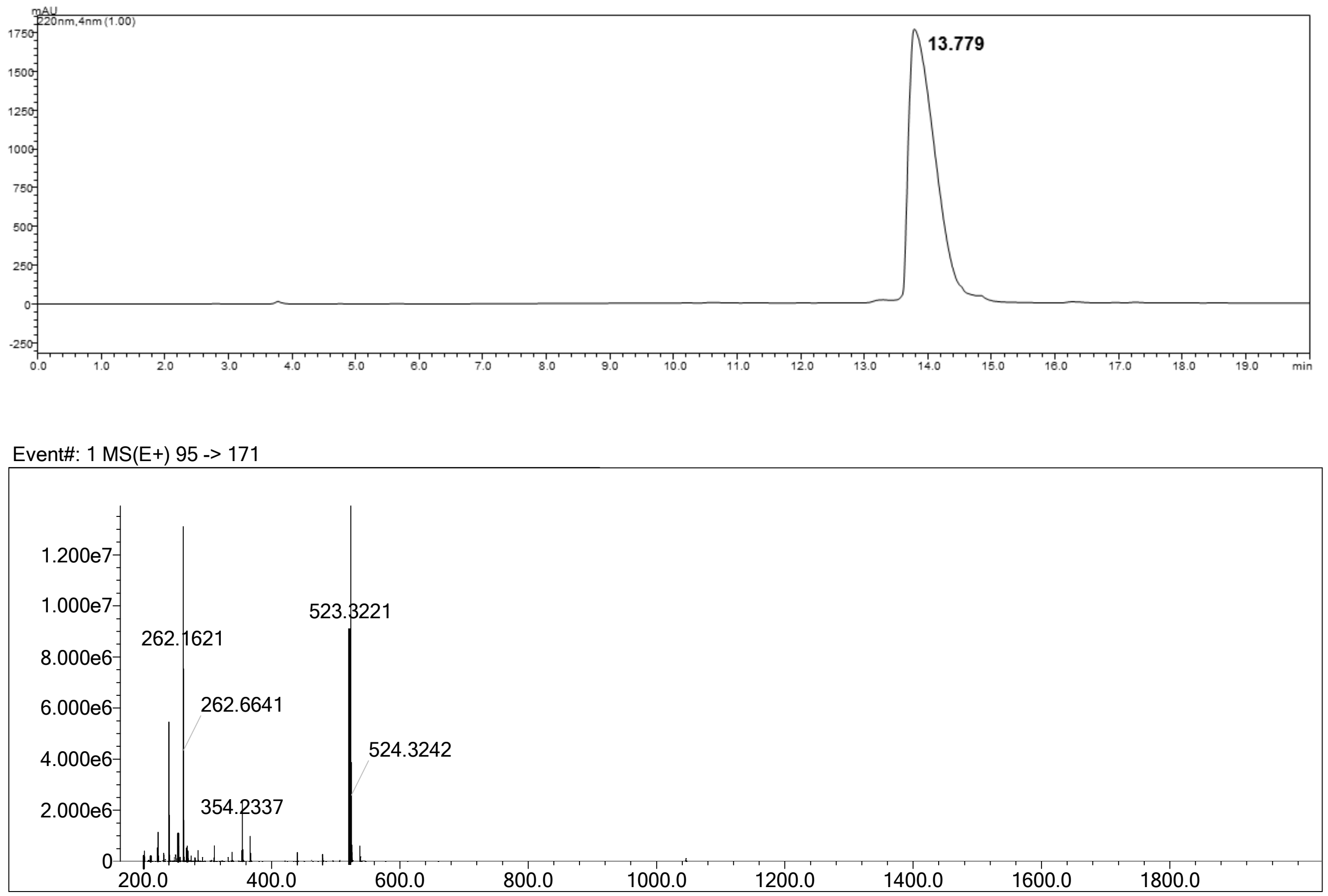

Rank	Score	Formula (M)	Ion	Meas. m/z	Pred. m/z	Df. (mDa)	Df. (ppm)	Iso	DBE
1	98.20	C21 H38 N12 O4	[M+H]+	523.3221	523.3212	0.9	1.72	100.00	9.0

Figure SM-SYN-12b. HPLC chromatogram of compound **12** at 220 nm and MS spectrum

Compound 13

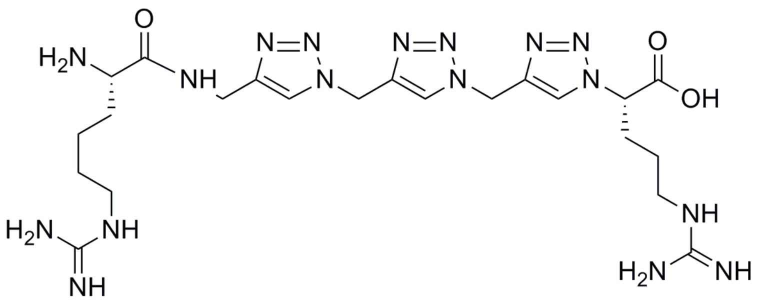

Figure SM-SYN-13a. Structure of compound **13** Har-GlyΨ[Trl]GlyΨ[Trl]GlyΨ[Trl]Arg.

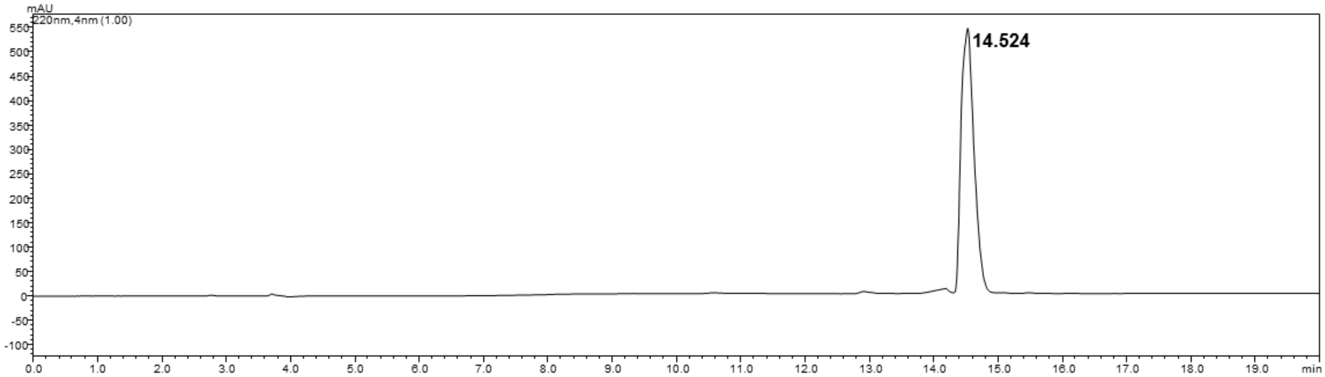

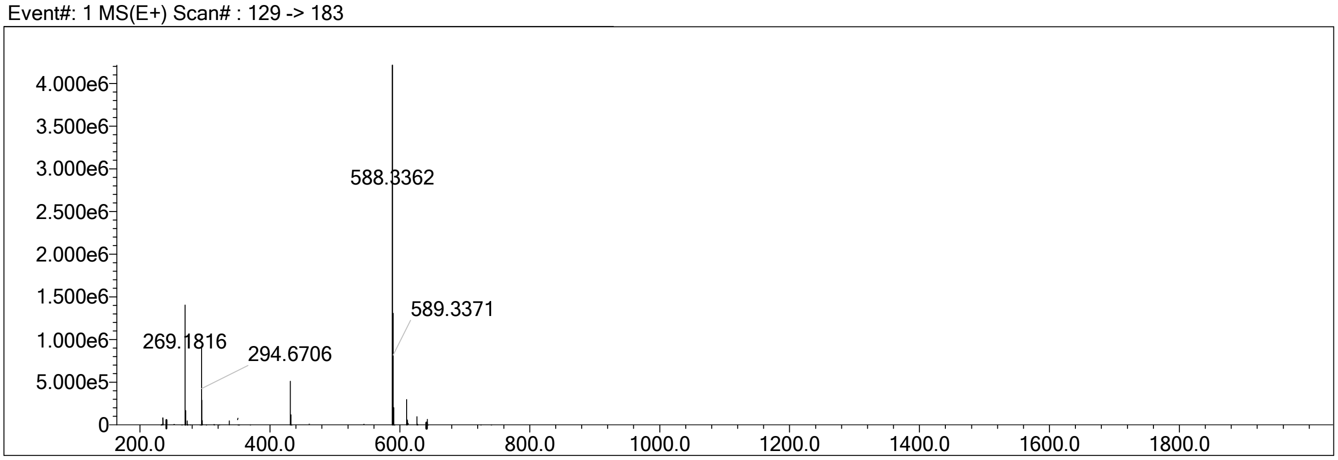

Rank	Score	Formula (M)	Ion	Meas. m/z	Pred. m/z	Df. (mDa)	Df. (ppm)	Iso	DBE
3	89.50	C22 H37 N17 O3	[M+H]+	588.3362	588.3338	2.4	4.08	96.97	13.0

Figure SM-SYN-13b. HPLC chromatogram of compound **13** at 220 nm and MS spectrum

Compound 14

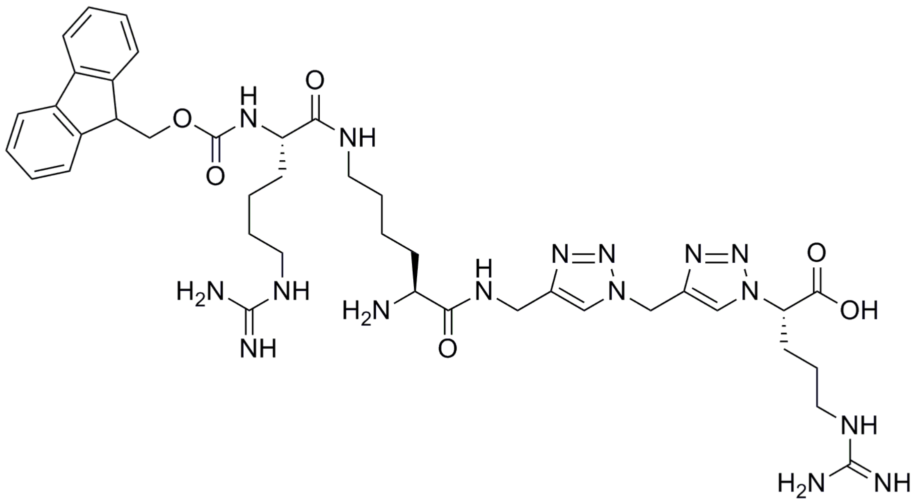

Figure SM-SYN-14a. Structure of compound **14** Lys(Fmoc-Har)-GlyΨ[Trl]GlyΨ[Trl]Arg.

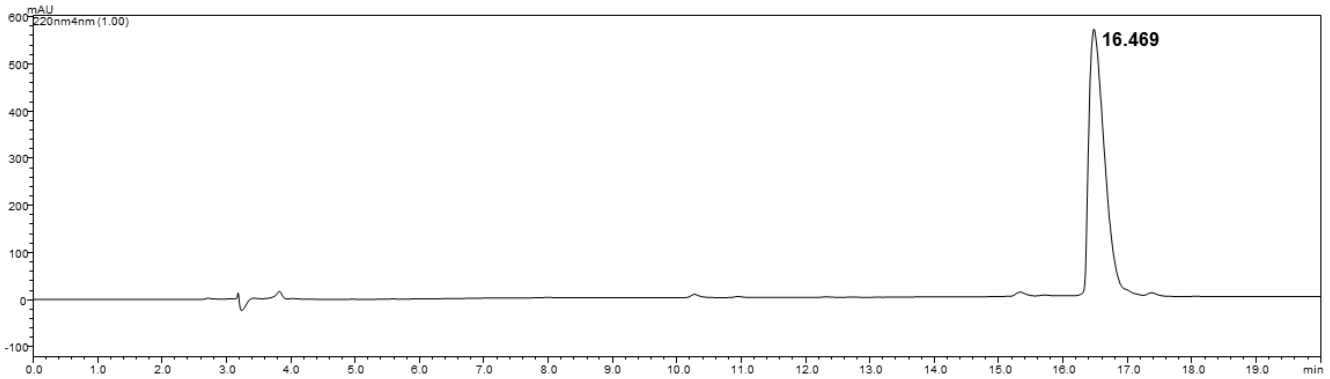

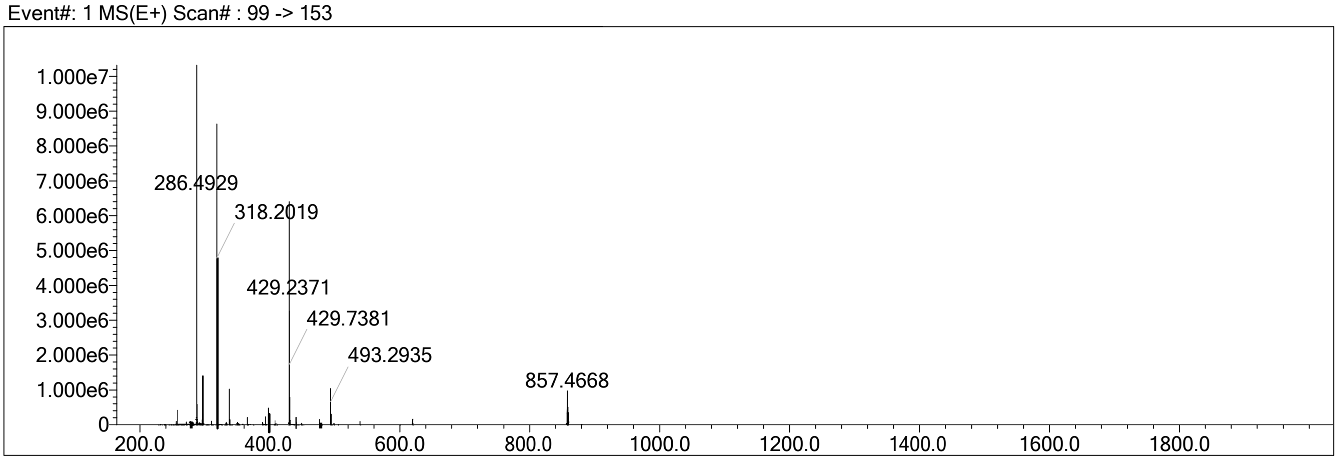

Rank	Score	Formula (M)	Ion	Meas. m/z	Pred. m/z	Df. (mDa)	Df. (ppm)	Iso	DBE
4	79.55	C40 H56 N16 O6	[M+2H]2+	429.2371	429.2357	1.4	3.26	84.31	21.0

Figure SM-SYN-14b. HPLC chromatogram of compound **14** at 220 nm and MS spectrum

Compound 15

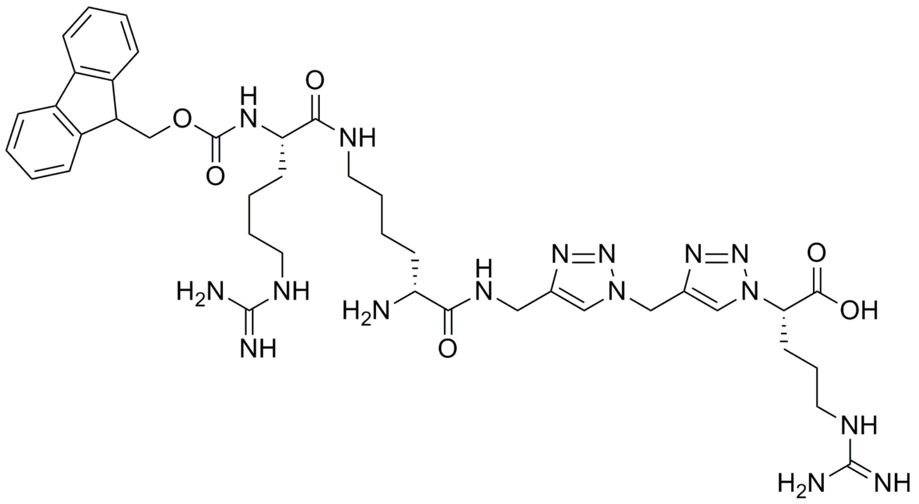

Figure SM-SYN-15a. Structure of compound **15** D-Lys(Fmoc-Har)-GlyΨ[Trl]GlyΨ[Trl]Arg.

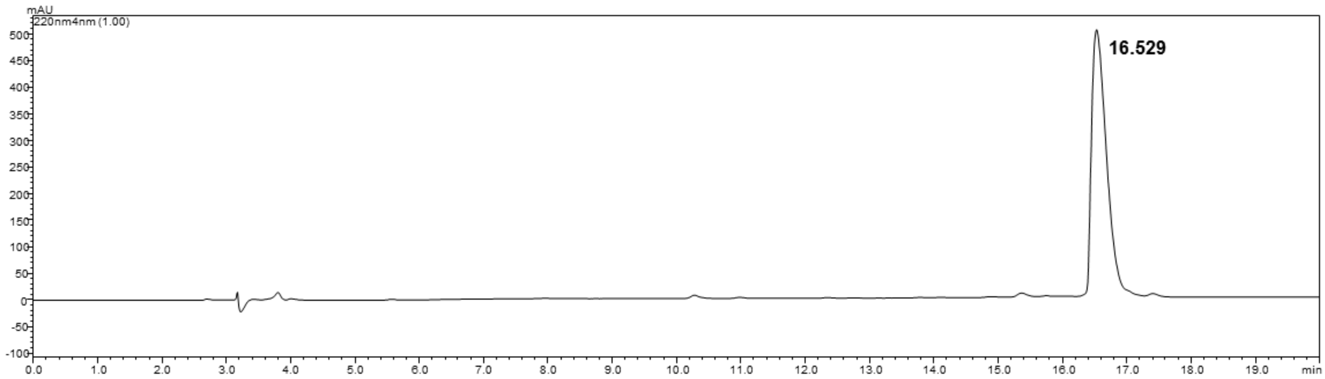

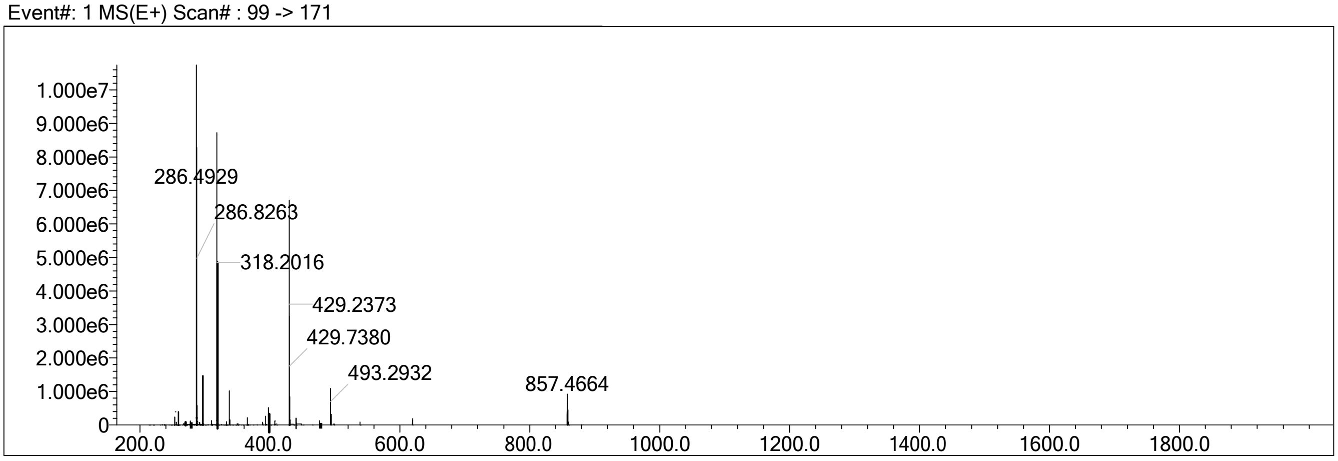

Rank	Score	Formula (M)	Ion	Meas. m/z	Pred. m/z	Df. (mDa)	Df. (ppm)	Iso	DBE
4	76.97	C40 H56 N16 O6	[M+2H]2+	429.2373	429.2357	1.6	3.73	82.61	21.0

Figure SM-SYN-15b. HPLC chromatogram of compound **15** at 220 nm and MS spectrum

Compound 16

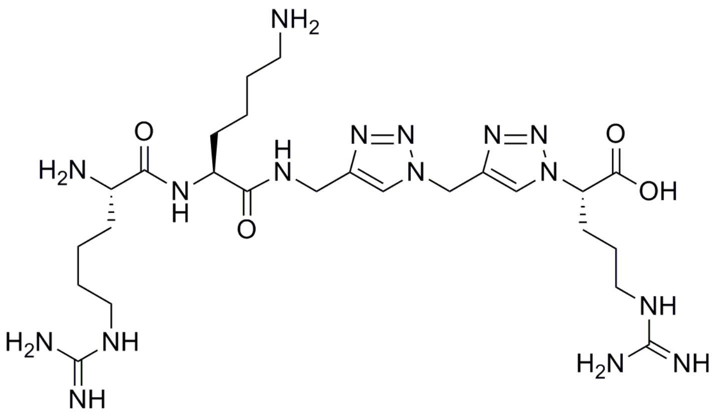

Figure SM-SYN-16a. Structure of compound **16** Har-Lys-GlyΨ[Trl]GlyΨ[Trl]Arg.

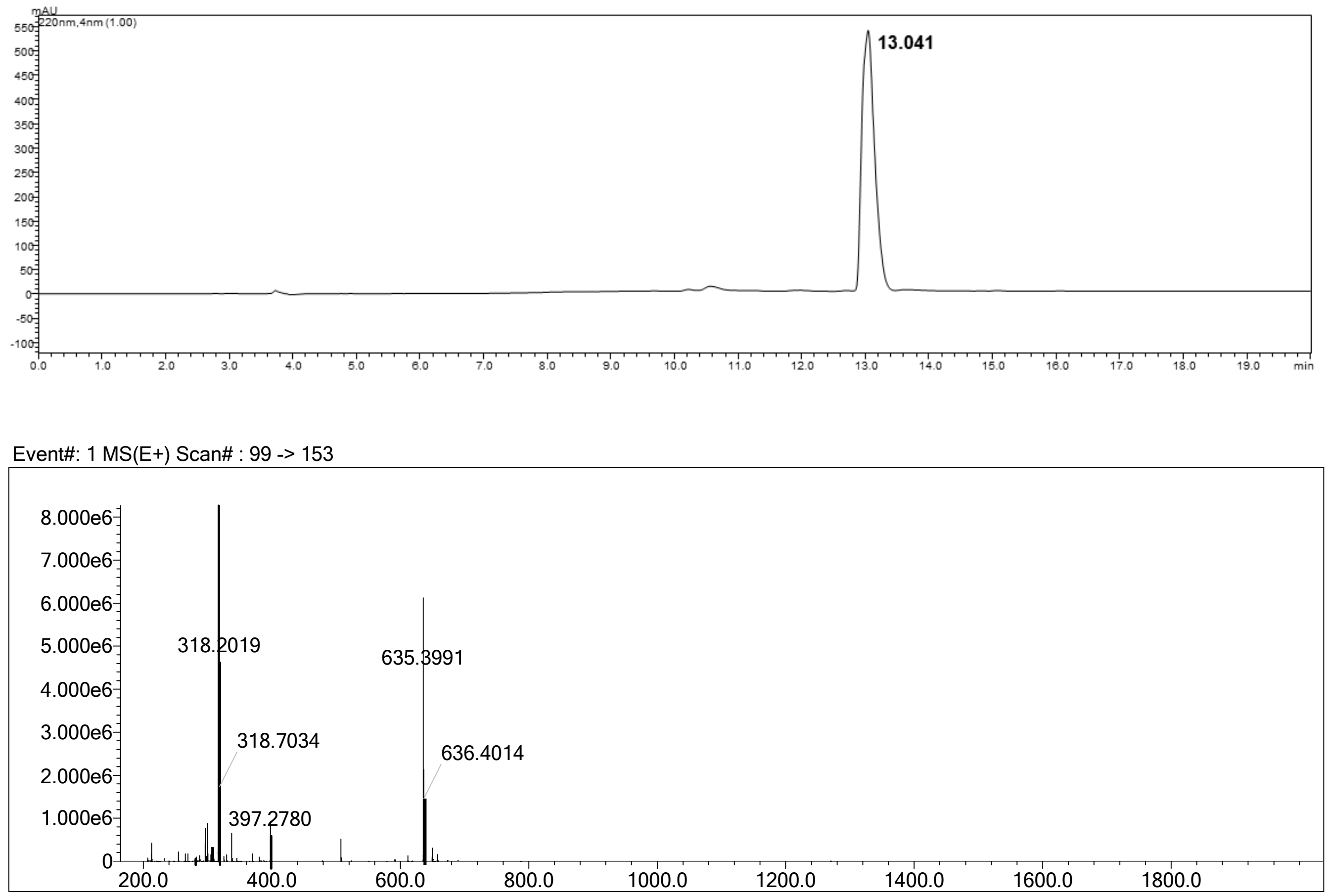

Rank	Score	Formula (M)	Ion	Meas. m/z	Pred. m/z	Df. (mDa)	Df. (ppm)	Iso	DBE
4	90.70	C25 H46 N16 O4	[M+H]+	635.3991	635.3961	3.0	4.72	100.00	11.0

Figure SM-SYN-16b. HPLC chromatogram of compound **16** at 220 nm and MS spectrum

Compound 17

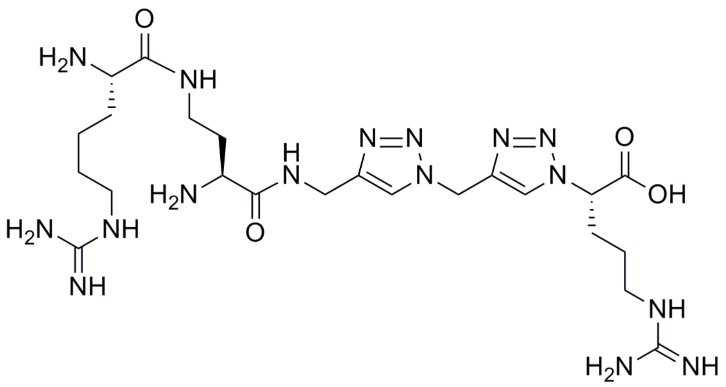

Figure SM-SYN-17a. Structure of compound **17** Dab(Har)-GlyΨ[Trl]GlyΨ[Trl]Arg.

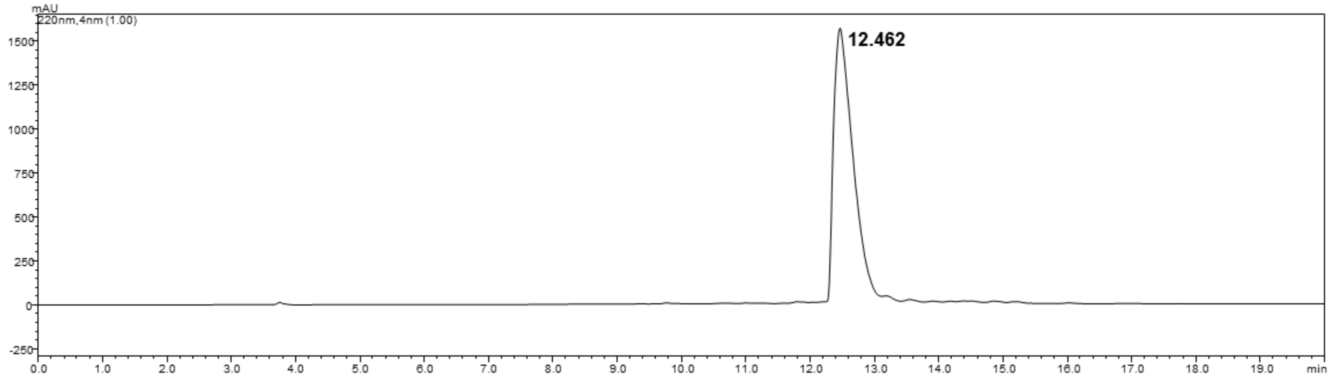

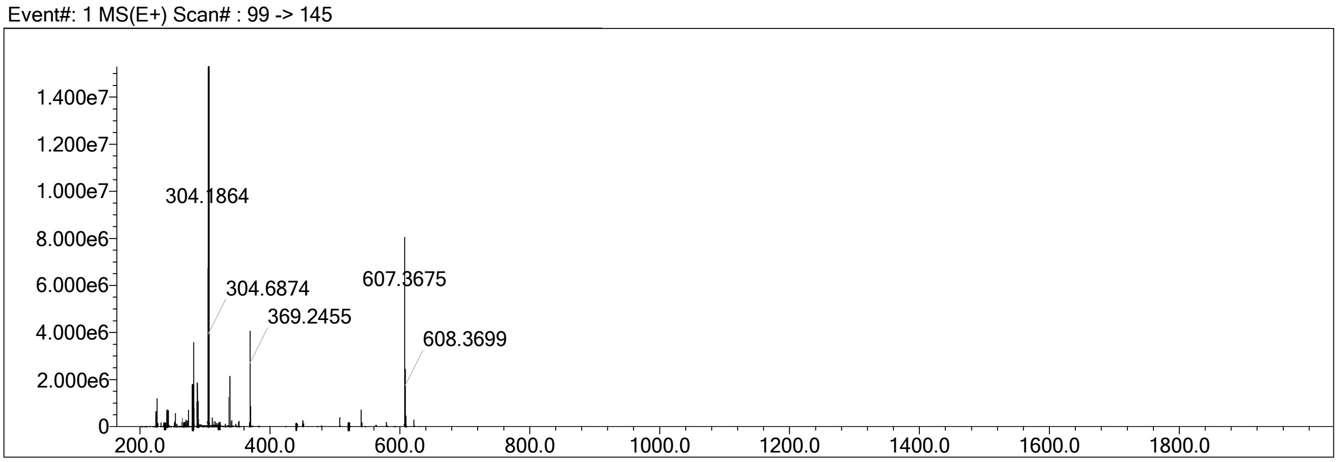

Rank	Score	Formula (M)	Ion	Meas. m/z	Pred. m/z	Df. (mDa)	Df. (ppm)	Iso	DBE
5	82.68	C23 H42 N16 O4	[M+H]+	607.3675	607.3648	2.7	4.45	90.49	11.0

Figure SM-SYN-17b. HPLC chromatogram of compound **17** at 220 nm and MS spectrum

Compound 18

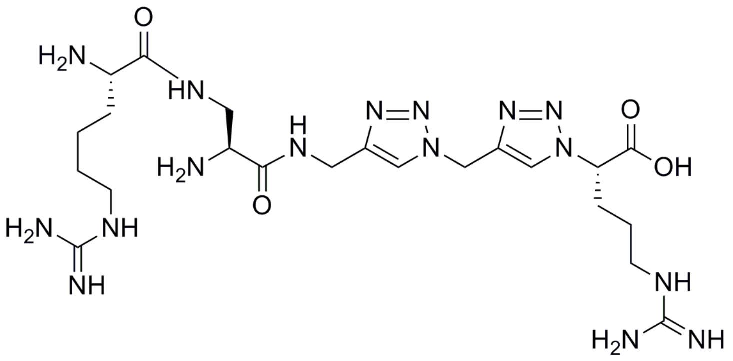

Figure SM-SYN-18a. Structure of compound **18** Dap(Har)-GlyΨ[Trl]GlyΨ[Trl]Arg.

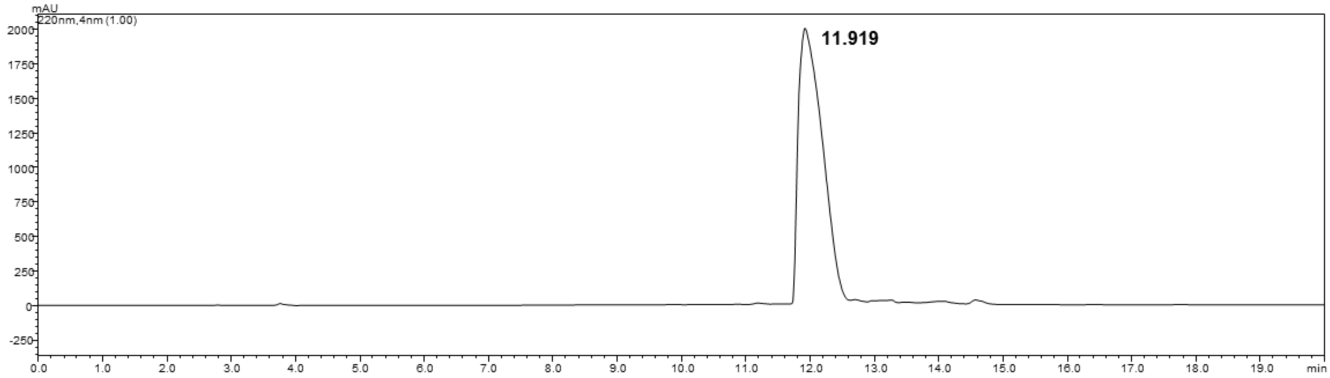

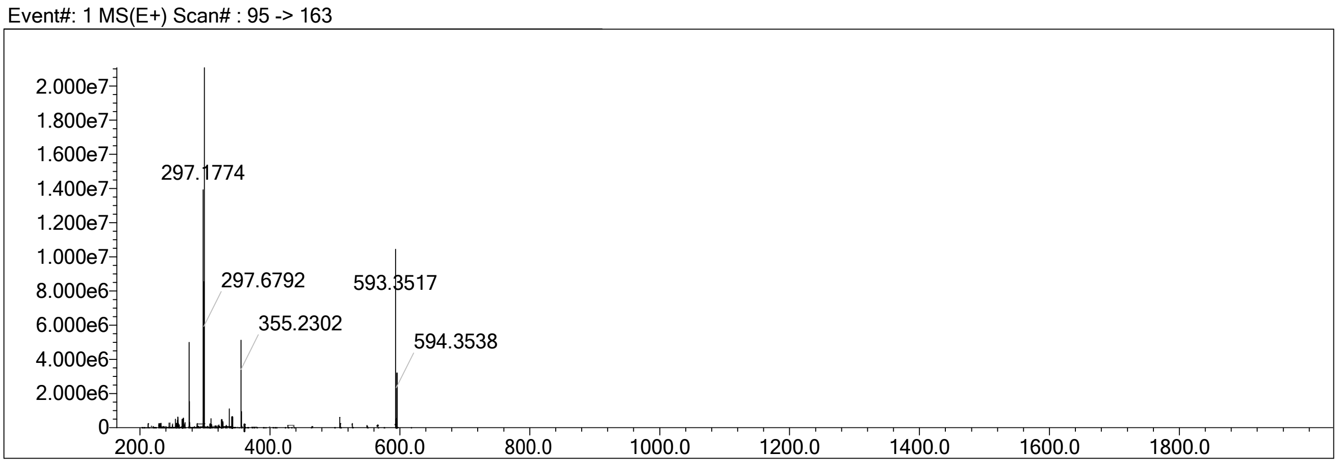

Rank	Score	Formula (M)	Ion	Meas. m/z	Pred. m/z	Df. (mDa)	Df. (ppm)	Iso	DBE
4	91.55	C22 H40 N16 O4	[M+H]+	593.3517	593.3491	2.6	4.38	100.00	11.0

Figure SM-SYN-18b. HPLC chromatogram of compound **18** at 220 nm and MS spectrum

Compound 19

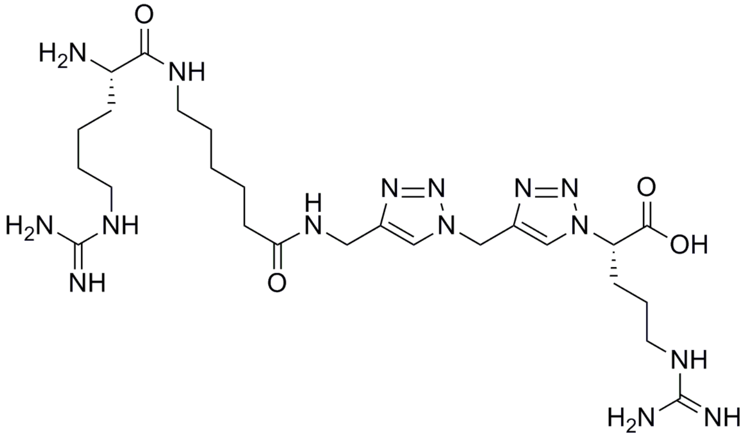

Figure SM-SYN-19a. Structure of compound **19** Har-6Ahx-GlyΨ[Trl]GlyΨ[Trl]]Arg.

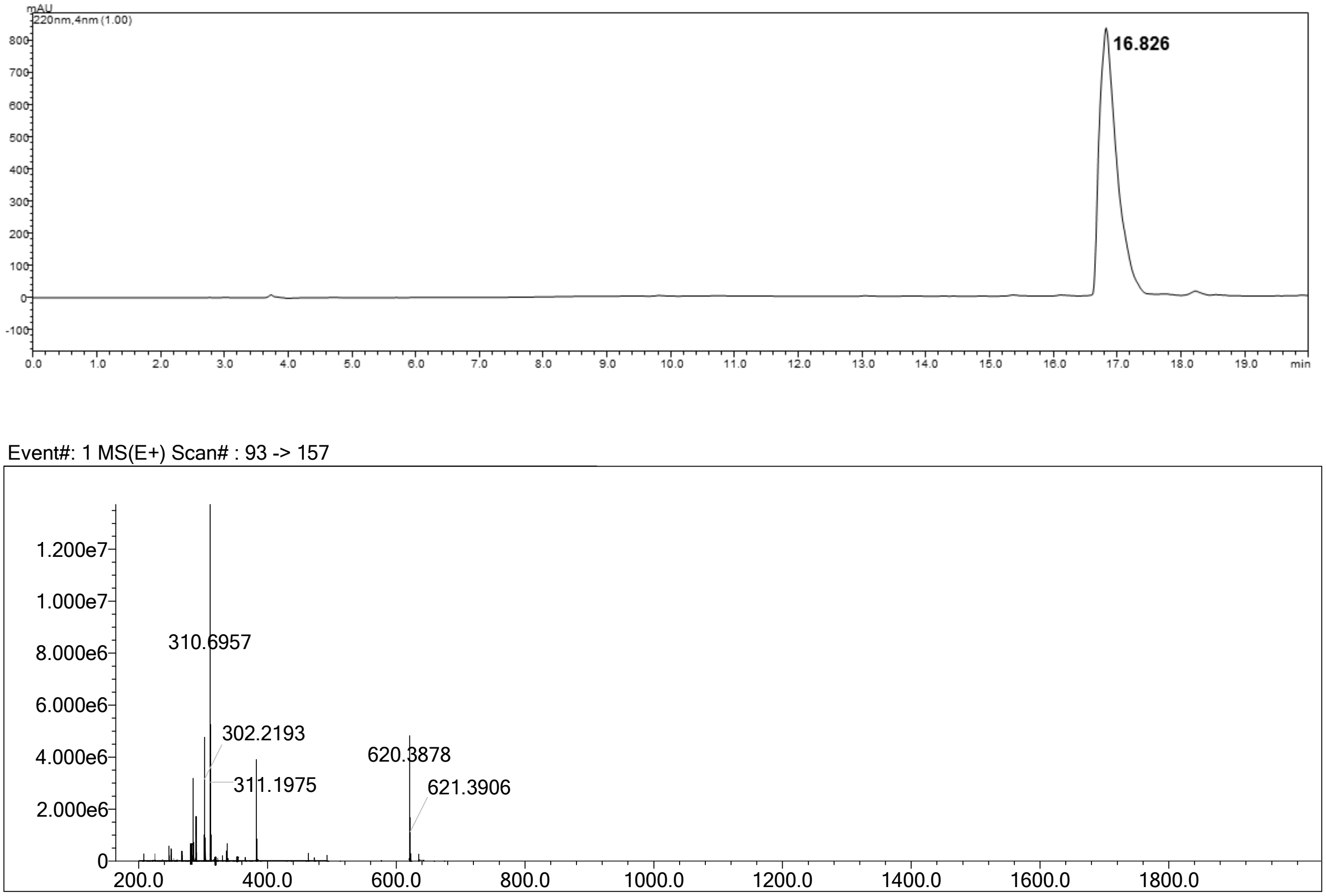

Rank	Score	Formula (M)	Ion	Meas. m/z	Pred. m/z	Df. (mDa)	Df. (ppm)	Iso	DBE
5	89.70	C25 H45 N15 O4	[M+H]+	620.3878	620.3852	2.6	4.19	97.47	11.0

Figure SM-SYN-19b. HPLC chromatogram of compound **19** at 220 nm and MS spectrum

Compound 20

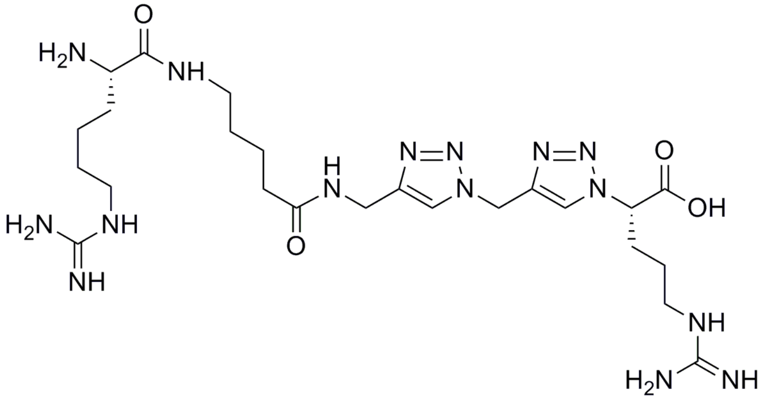

Figure SM-SYN-20a. Structure of compound **20** Har-5Ava-GlyΨ[Trl]GlyΨ[Trl]Arg.

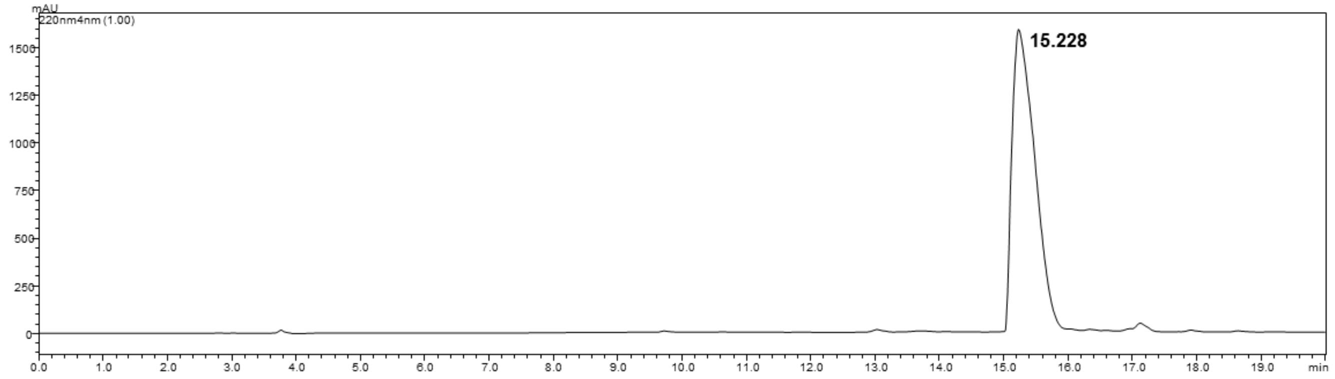

Event#: 1 MS(E+) Scan# : 89 -> 159

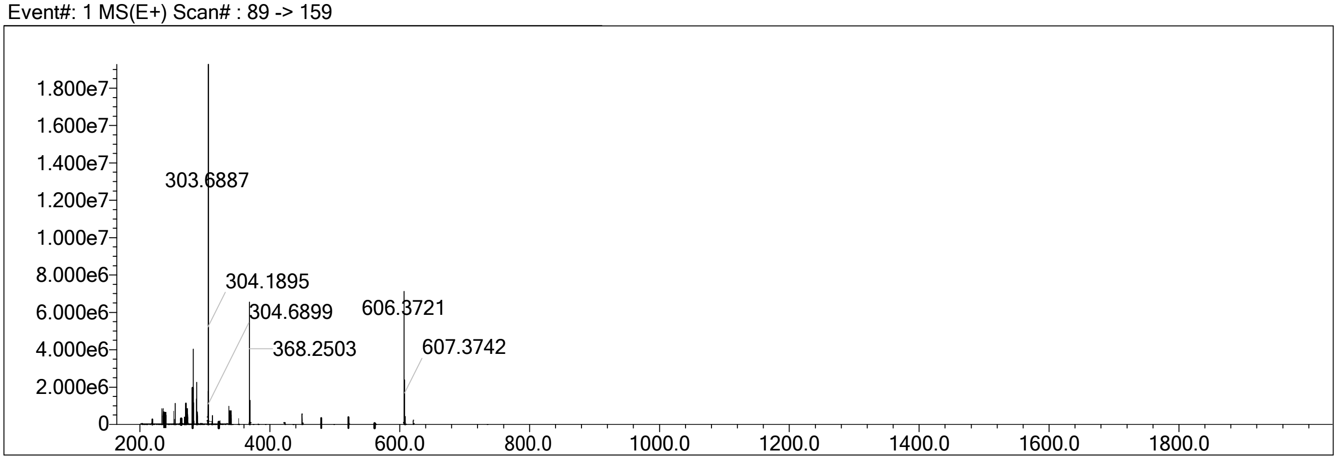

Rank	Score	Formula (M)	Ion	Meas. m/z	Pred. m/z	Df. (mDa)	Df. (ppm)	Iso	DBE
4	91.78	C24 H43 N15 O4	[M+H]+	606.3721	606.3695	2.6	4.29	100.00	11.0

Figure SM-SYN-20b. HPLC chromatogram of compound **20** at 220 nm and MS spectrum

Compound 21

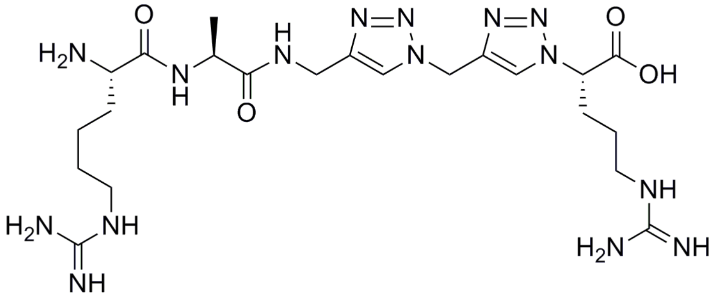

Figure SM-SYN-21a. Structure of compound **21** Har-Ala-GlyΨ[Trl]GlyΨ[Trl]Arg.

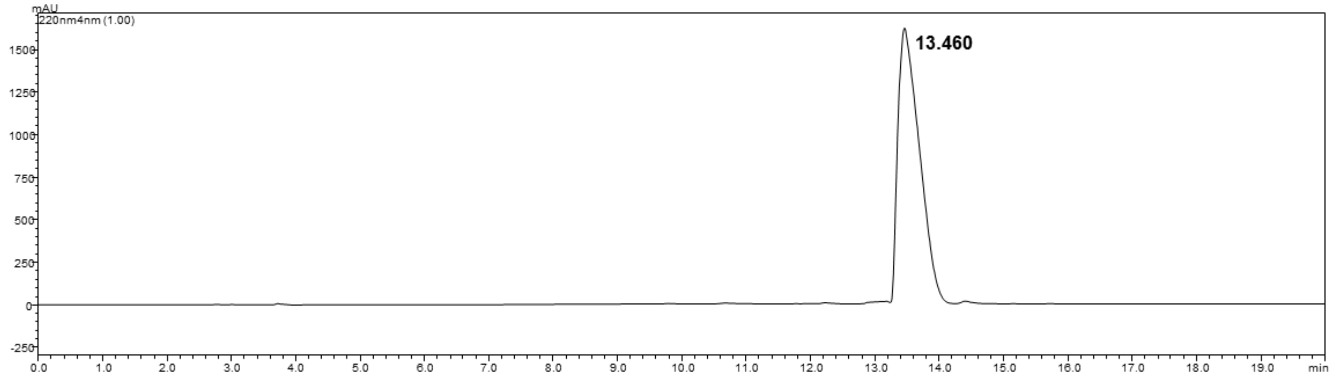

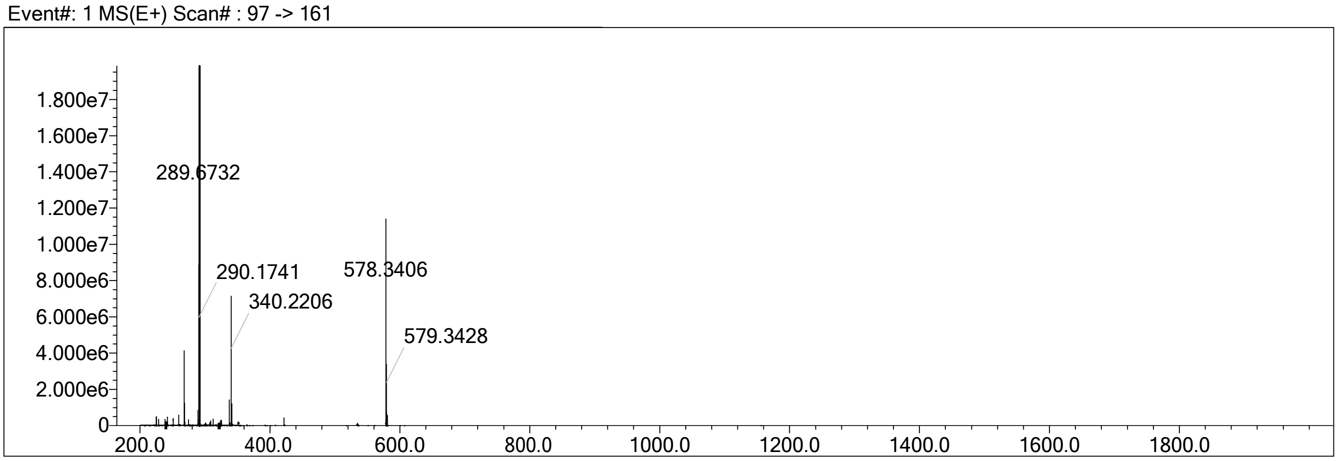

Rank	Score	Formula (M)	Ion	Meas. m/z	Pred. m/z	Df. (mDa)	Df. (ppm)	Iso	DBE
4	92.13	C22 H39 N15 O4	[M+H]+	578.3406	578.3382	2.4	4.15	100.00	11.0

Figure SM-SYN-21b. HPLC chromatogram of compound **21** at 220 nm and MS spectrum

Compound 22

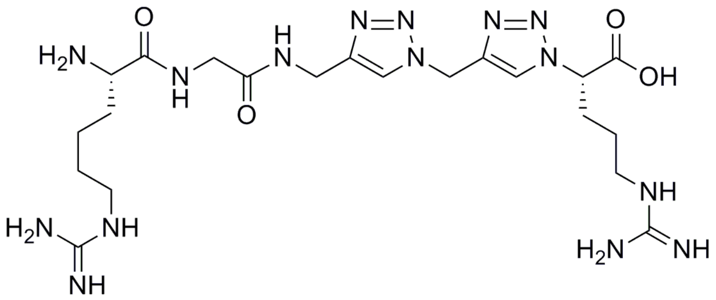

Figure SM-SYN-22a. Structure of compound **22** Har-Gly-GlyΨ[Trl]GlyΨ[Trl]Arg.

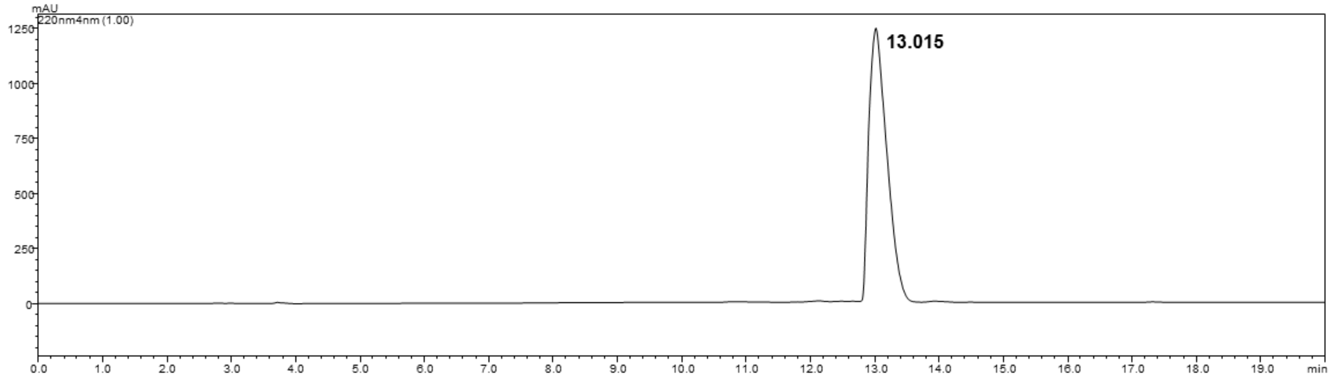

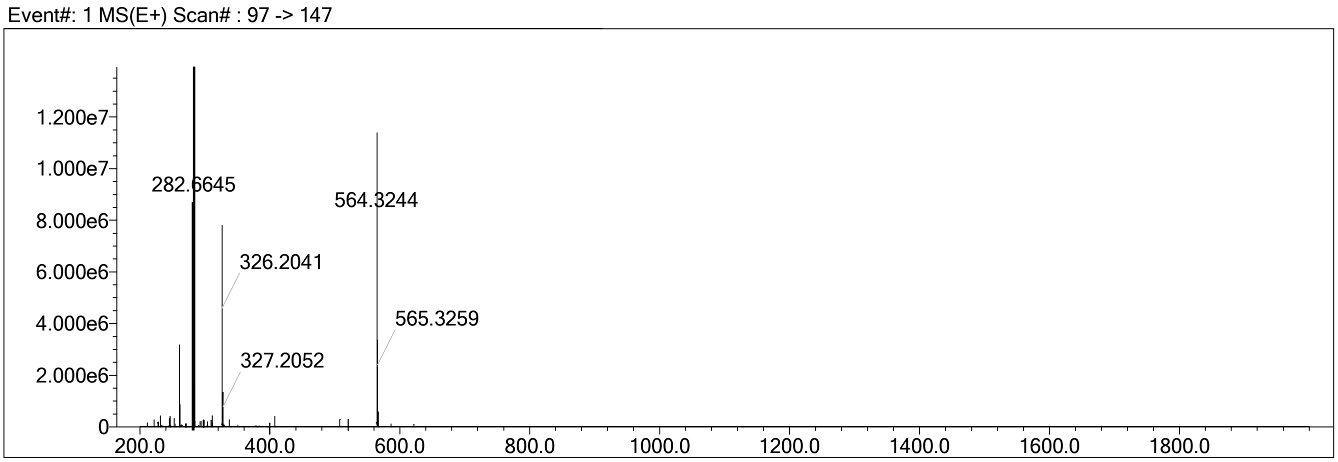

Rank	Score	Formula (M)	Ion	Meas. m/z	Pred. m/z	Df. (mDa)	Df. (ppm)	Iso	DBE
4	94.53	C21 H37 N15 O4	[M+H]+	564.3244	564.3226	1.8	3.19	100.00	11.0

Figure SM-SYN-22b. HPLC chromatogram of compound **22** at 220 nm and MS spectrum

Compound 23

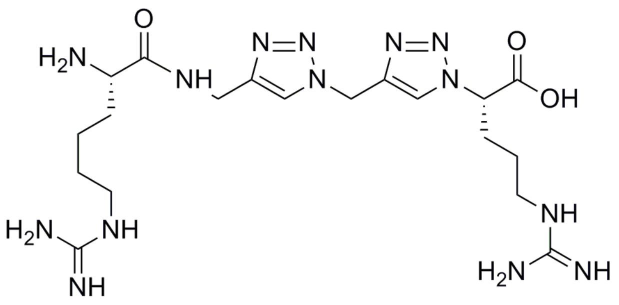

Figure SM-SYN-23a. Structure of compound **23** Har-GlyΨ[Trl]GlyΨ[Trl]Arg.

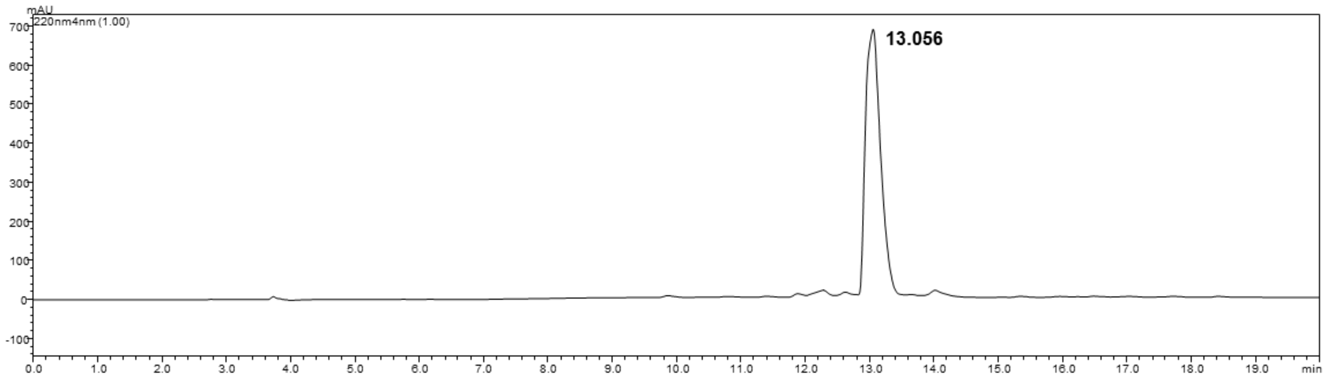

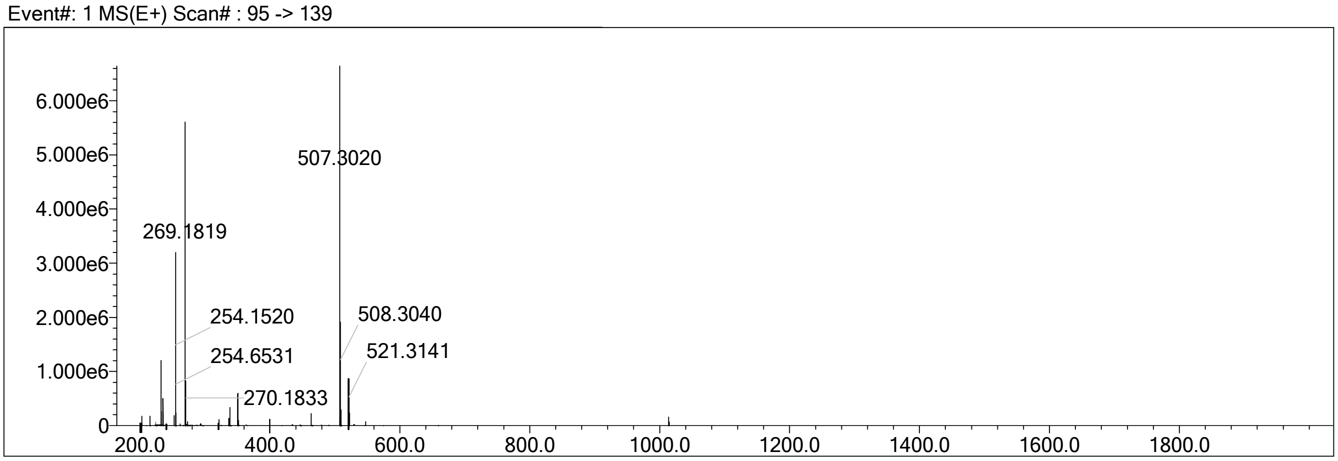

Rank	Score	Formula (M)	Ion	Meas. m/z	Pred. m/z	Df. (mDa)	Df. (ppm)	Iso	DBE
2	98.08	C19 H34 N14 O3	[M+H]+	507.3020	507.3011	0.9	1.77	100.00	10.0

Figure SM-SYN-23b. HPLC chromatogram of compound **23** at 220 nm and MS spectrum

Compound (24)

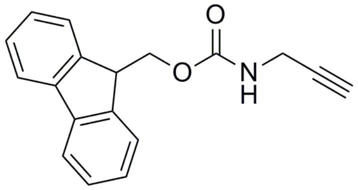

Figure SM-SYN-24a. Structure of compound **24** Fmoc-propargylamine

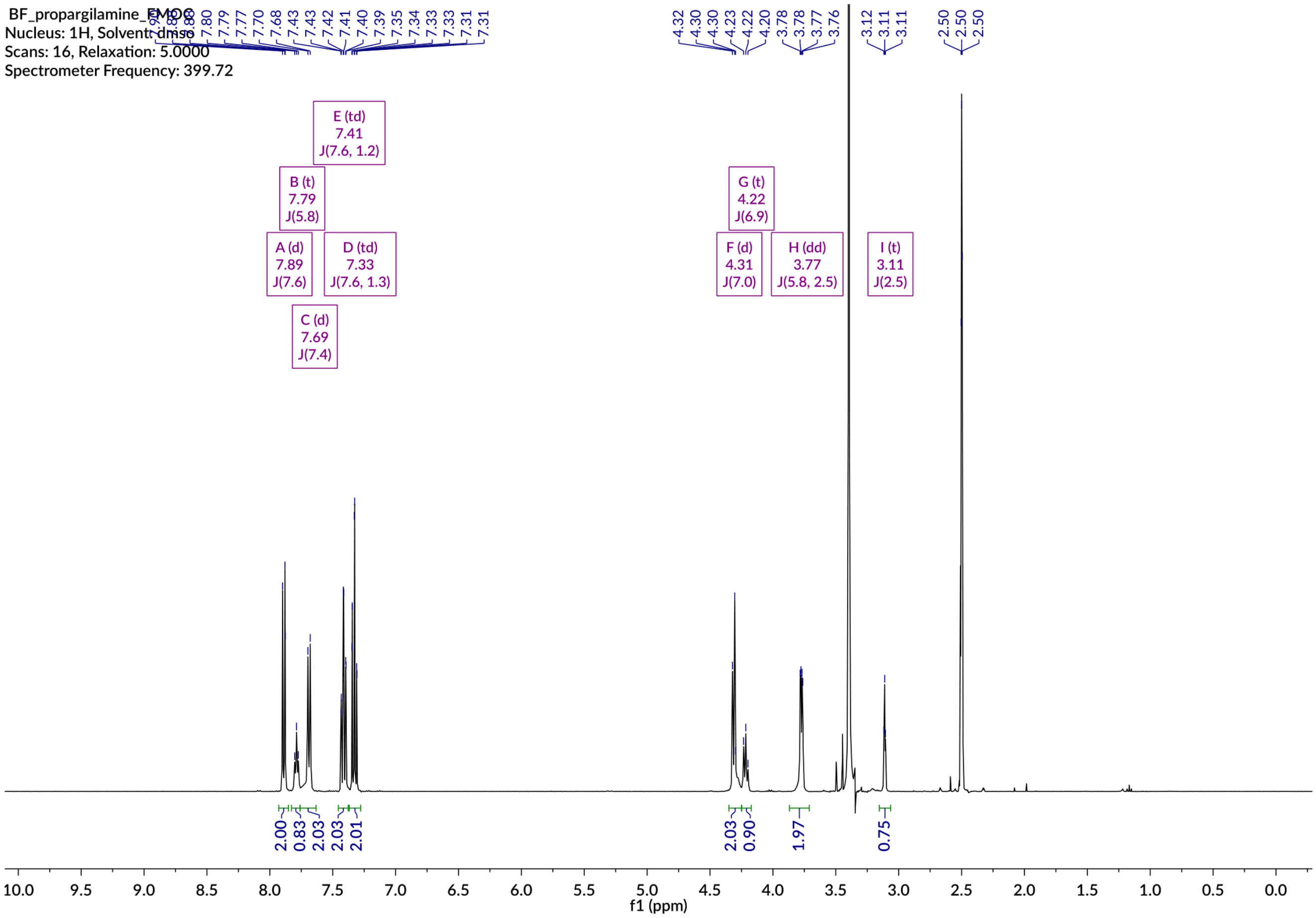

Figure SM-SYN-24b. ^{1}H NMR spectrum of compound **24** Fmoc-propargylamine

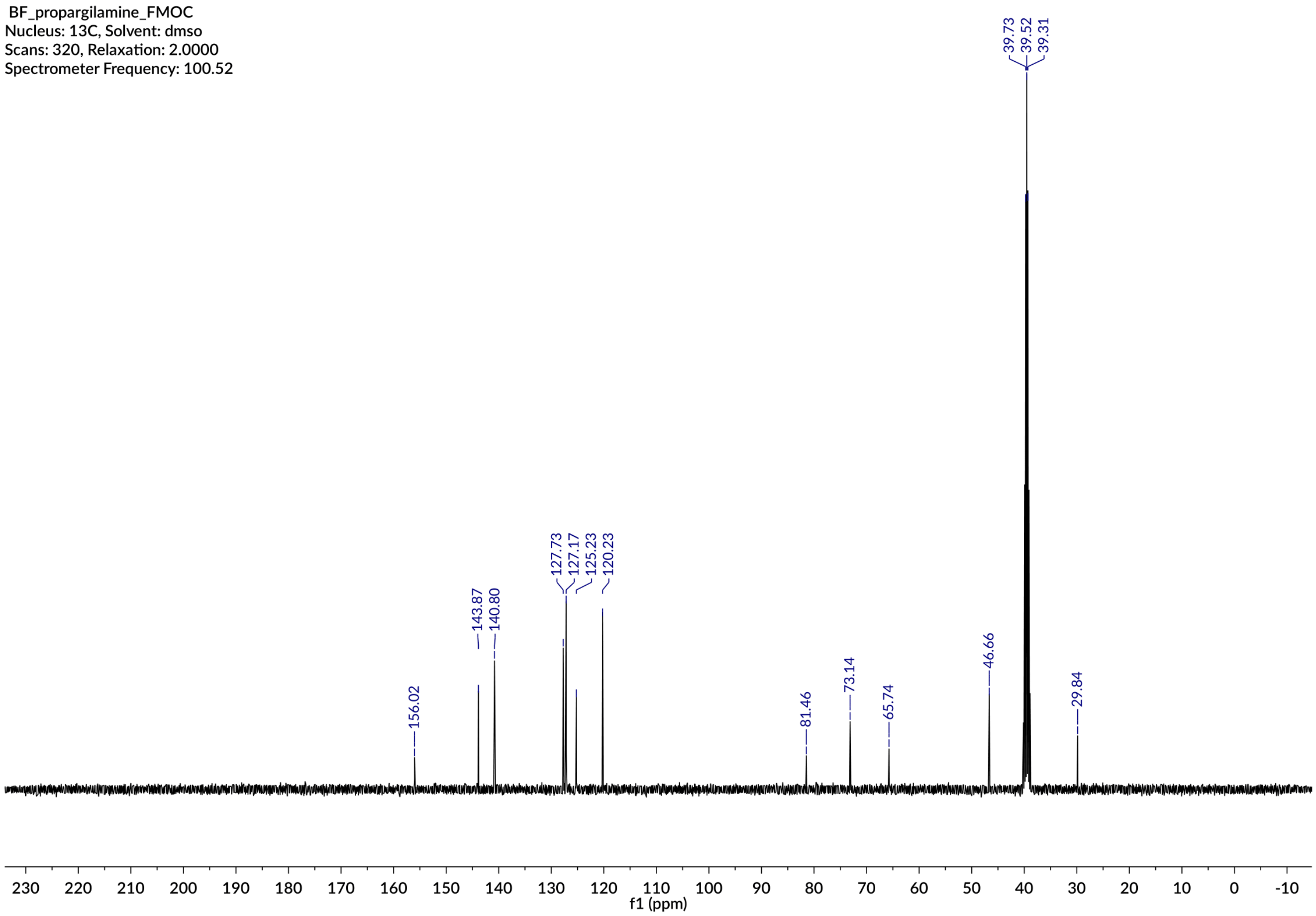

Figure SM-SYN-24c. ^{13}C NMR spectrum of compound **24** Fmoc-propargylamine

SM-INH. Inhibitory activity

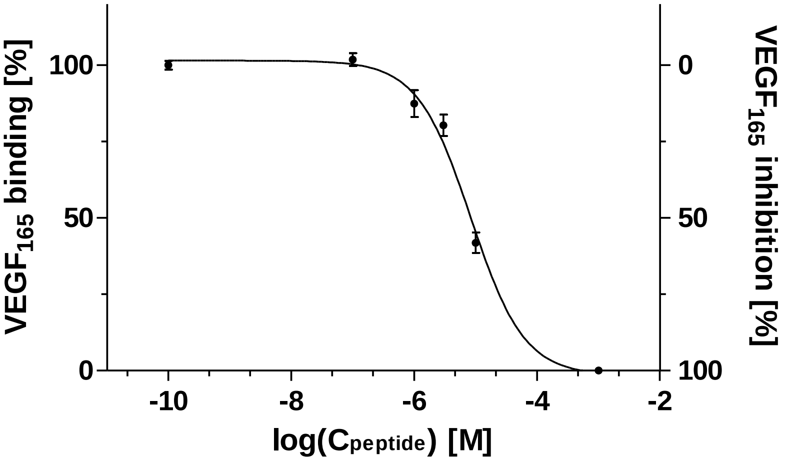

R^2 = 0,9859

logIC50 = -5,076 ± 0,06335

IC50 = 8,390e-006 M

IC50 = 8,39 ± 1,023 uM

Figure SM-INH-1. Dose-response curve for compound **3** Lys(Har)-GlyΨ[Trl]GlyΨ[Trl]Arg

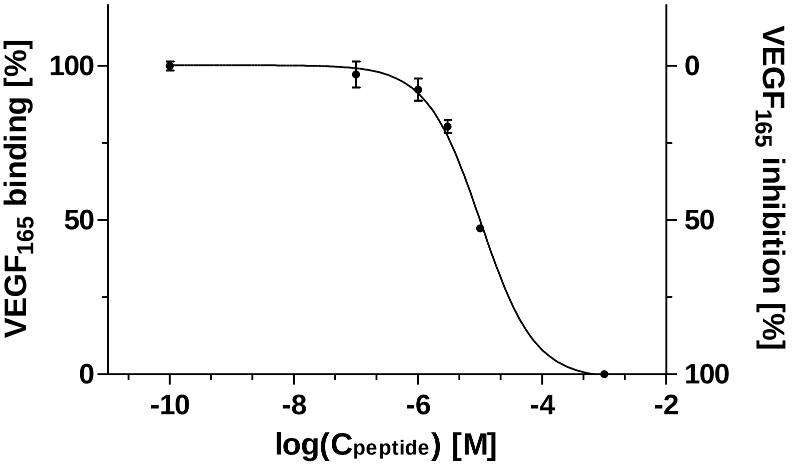

R^2 = 0,9921

logIC50 = -4,991 ± 0,04799

IC50 = 1,022e-005 M

IC50 = 10,22 ± 0,638 uM

Figure SM-INH-2. Dose-response curve for compound **4** D-Lys(Har)-GlyΨ[Trl]GlyΨ[Trl]Arg

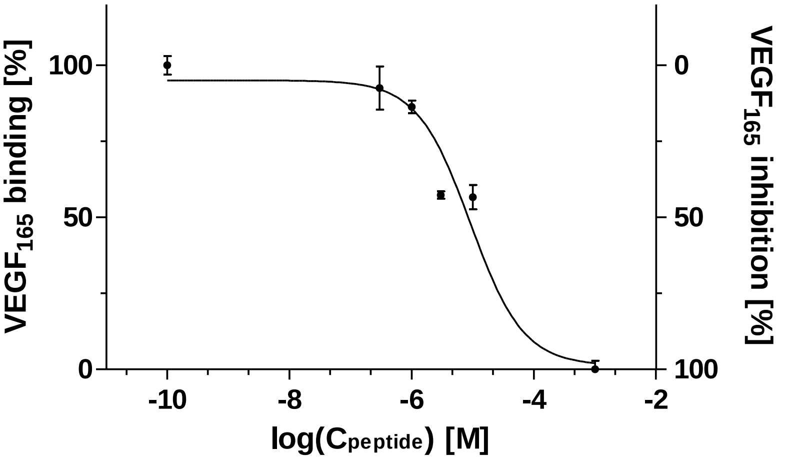

R^2 = 0,9358

logIC50 = -5,040 ± 0,1417

IC50 = 9,111e-006 M

IC50 = 9,11 ± 1,1 uM

Figure SM-INH-3. Dose-response curve for compound **5** D-Lys(D-Har)-GlyΨ[Trl]GlyΨ[Trl]Arg.

SM-COR. Correlational analysis

Table SM-COR-1. Variable matrix for correlation analysis. Variable symbols explained in Table SM-COR-2.

	sequence	% activity at 10 µM	dis_{N-C}	$dis_{N-C, norm}$	$1Trl_{C-N}$	$2Trl_{C-N}$	$3Trl_{C-N}$	am_{1N}	am_{2N}	bb	L_{BR}	L_{2N}
**1**	H-Lys(hArg)-GlyΨ[Trl]Arg-OH	28.1	25	0.44	1	0	0	1	1	0	1	1
**2**	H-D-Lys(hArg)-GlyΨ[Trl]Arg-OH	33.1	25	0.44	1	0	0	1	1	0	1	0
**3**	H-Lys(hArg)-GlyΨ[Trl]GlyΨ[Trl]Arg-OH	58.1	30	1.00	1	1	0	1	1	0	1	1
**4**	H-D-Lys(hArg)-GlyΨ[Trl]GlyΨ[Trl]Arg-OH	52.6	30	1.00	1	1	0	1	1	0	1	0
**5**	H-D-Lys(D-hArg)-GlyΨ[Trl]GlyΨ[Trl]Arg-OH	48.5	30	1.00	1	1	0	1	1	0	0	0
**6**	H-Lys(hArg)-Pro-GlyΨ[Trl]Arg-OH	18.3	28	0.78	1	0	0	1	1	0	1	1
**7**	H-D-Lys(hArg)-Pro-GlyΨ[Trl]Arg-OH	38.4	28	0.78	1	0	0	1	1	0	1	0
**8**	H-Lys(hArg)-Phe-GlyΨ[Trl]Arg-OH	30.9	28	0.78	1	0	0	1	1	0	1	1
**9**	H-D-Lys(hArg)-Phe-GlyΨ[Trl]Arg-OH	37.9	28	0.78	1	0	0	1	1	0	1	0
**10**	H-Lys(hArg)-GlyΨ[Trl]Ala-Arg-OH	43.3	28	0.78	0	1	0	1	1	0	1	1
**11**	H-Lys(hArg)-GlyΨ[Trl]Gly-Arg-OH	30.6	28	0.78	0	1	0	1	1	0	1	1
**12**	H-hArg-Pro-GlyΨ[Trl]Arg-OH	9.2	21	0.00	1	0	0	1	0	1	1	1
**13**	H-hArg-GlyΨ[Trl]GlyΨ[Trl]GlyΨ[Trl]Arg-OH	34.7	28	0.78	1	1	1	1	0	1	1	1
**14**	H-Lys(Fmocα-hArg)-GlyΨ[Trl]GlyΨ[Trl]Arg-OH	57.7	30	1.00	1	1	0	0	1	0	1	1
**15**	H-D-Lys(Fmocα-hArg)-GlyΨ[Trl]GlyΨ[Trl]Arg-OH	43.7	30	1.00	1	1	0	0	1	0	1	0
**16**	H-hArg-Lys-GlyΨ[Trl]GlyΨ[Trl]Arg-OH	36.5	26	0.56	1	1	0	1	0	1	1	1
**17**	H-Dab(hArg)-GlyΨ[Trl]GlyΨ[Trl]Arg-OH	41.3	28	0.78	1	1	0	1	1	0	1	1
**18**	H-Dap(hArg)-GlyΨ[Trl]GlyΨ[Trl]Arg-OH	25.3	27	0.67	1	1	0	1	1	0	1	1
**19**	H-hArg-6Ahx-GlyΨ[Trl]GlyΨ[Trl]]Arg-OH	30.5	30	1.00	1	1	0	1	0	1	1	0.5
**20**	H-hArg-5Ava-GlyΨ[Trl]GlyΨ[Trl]Arg-OH	29.7	29	0.89	1	1	0	1	0	1	1	0.5
**21**	H-hArg-Ala-GlyΨ[Trl]GlyΨ[Trl]]Arg-OH	27.8	26	0.56	1	1	0	1	0	1	1	1
**22**	H-hArg-Gly-GlyΨ[Trl]GlyΨ[Trl]Arg-OH	35.7	26	0.56	1	1	0	1	0	1	1	0.5
**23**	H-hArg-GlyΨ[Trl]GlyΨ[Trl]Arg-OH	20	23	0.22	1	1	0	1	0	1	1	1

Table SM-COR-2. Explanation of variables symbols.

$\boldsymbol{dis_{N\text{-}C}}$	topological distance between guanidines at N to C direction
$\boldsymbol{1Trl_{C\text{-}N}}$	triazole unit instead of the 1st amide/peptide bond C to N direction
$\boldsymbol{2Trl_{C\text{-}N}}$	triazole unit instead of the 2nd amide/peptide bond C to N direction
$\boldsymbol{3Trl_{C\text{-}N}}$	triazole unit instead of the 3rd amide/peptide bond C to N direction
$\boldsymbol{am_{1N}}$	free amine present by the 1st amide/peptide bond (going from the N-terminus or from the residue attached to the N-terminal side chain)
$\boldsymbol{am_{2N}}$	free amine present by the 2nd amide/peptide bond (going from the N-terminus or from the residue attached to the N-terminal side chain)
bb	backbone (1) or sidechain (0) coupling at N-terminus
$\boldsymbol{L_{1N}}$	L-amino acid at the residue attached to the N-terminal side chain
$\boldsymbol{L_{2N}}$	L-amino acid at the N-terminal residue; 0.5 value means achiral AA

Listing SM-COR-1. Equations of the models developed by correlational analysis.

1. $act = 3.7 * dis_{N\text{-}C} - 67.3$, $R^2 = 0.54$, n = 23 (MODEL 1)
2. $act = 8.6\ (\pm 5.0) + 20.9\ (\pm 8.6) * dis_{N\text{-}C,\ norm} + 8.7\ (\pm 4.5) * am_{2N} + 8.6\ (\pm 4.6) * 2Trl_{C\text{-}N}$, $R^2 = 0.63$, n = 23. (MODEL 2)
3. $act = -\ 34.7\ (\pm 27.2) + 2.0\ (\pm 1.1) * dis_{N\text{-}C} + 6.2\ (\pm 6.9) * 1Trl_{C\text{-}N} + 10.0\ (\pm 5.3) * 2Trl_{C\text{-}N} + 2.0\ (\pm 9.0) * 3Trl_{C\text{-}N} - 3.6\ (\pm 6.8) * am_{1N} + 10.5\ (\pm 5.5) * am_{2N}$, $R^2 = 0.66$, n = 23.
4. $act = -\ 41.8\ (\pm 23.0) + 2.0\ (\pm 1.0) * dis_{N\text{-}C} + 7.0\ (\pm 6.4) * 1Trl_{C\text{-}N} + 10.6\ (\pm 4.9) * 2Trl_{C\text{-}N} + 10.8\ (\pm 4.9) * am_{2N}$, $R^2 = 0.66$, n = 23.
5. $act = -\ 25.9\ (\pm 26.0) + 1.9\ (\pm 1.0) * dis_{N\text{-}C} + 10.3\ (\pm 4.8) * 2Trl_{C\text{-}N} + 9.1\ (\pm 4.5) * am_{2N} - 4.9\ (\pm 4.2) * L_{2N}$, $R^2 = 0.66$, n = 23.

SM-SIM. Simulations

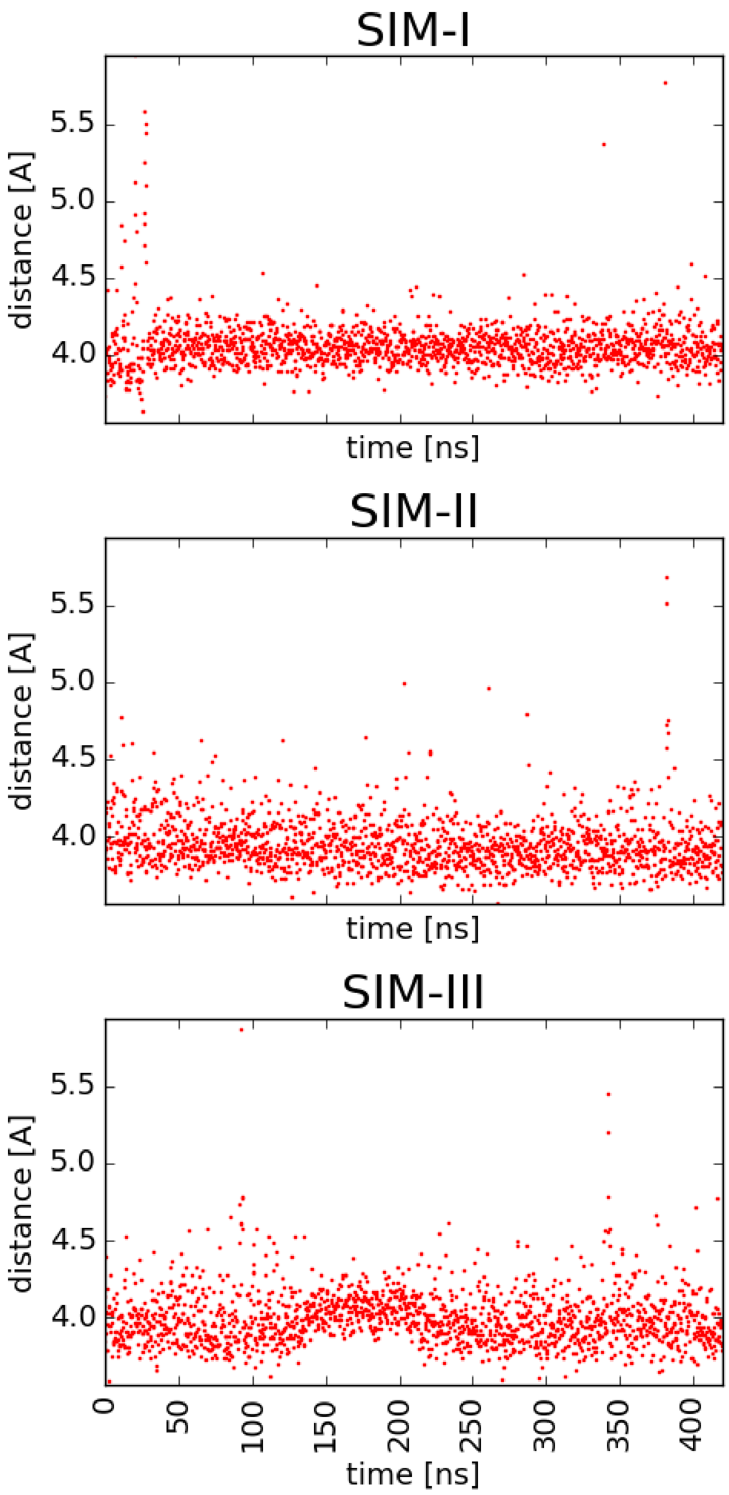

Figure SM-SIM-1. Time evolution of distance between Cγ atom of Asp320 and Cζ of Arg residue in compound 3. Data come from simulations SIM-I, SIM-II, SIM-III.

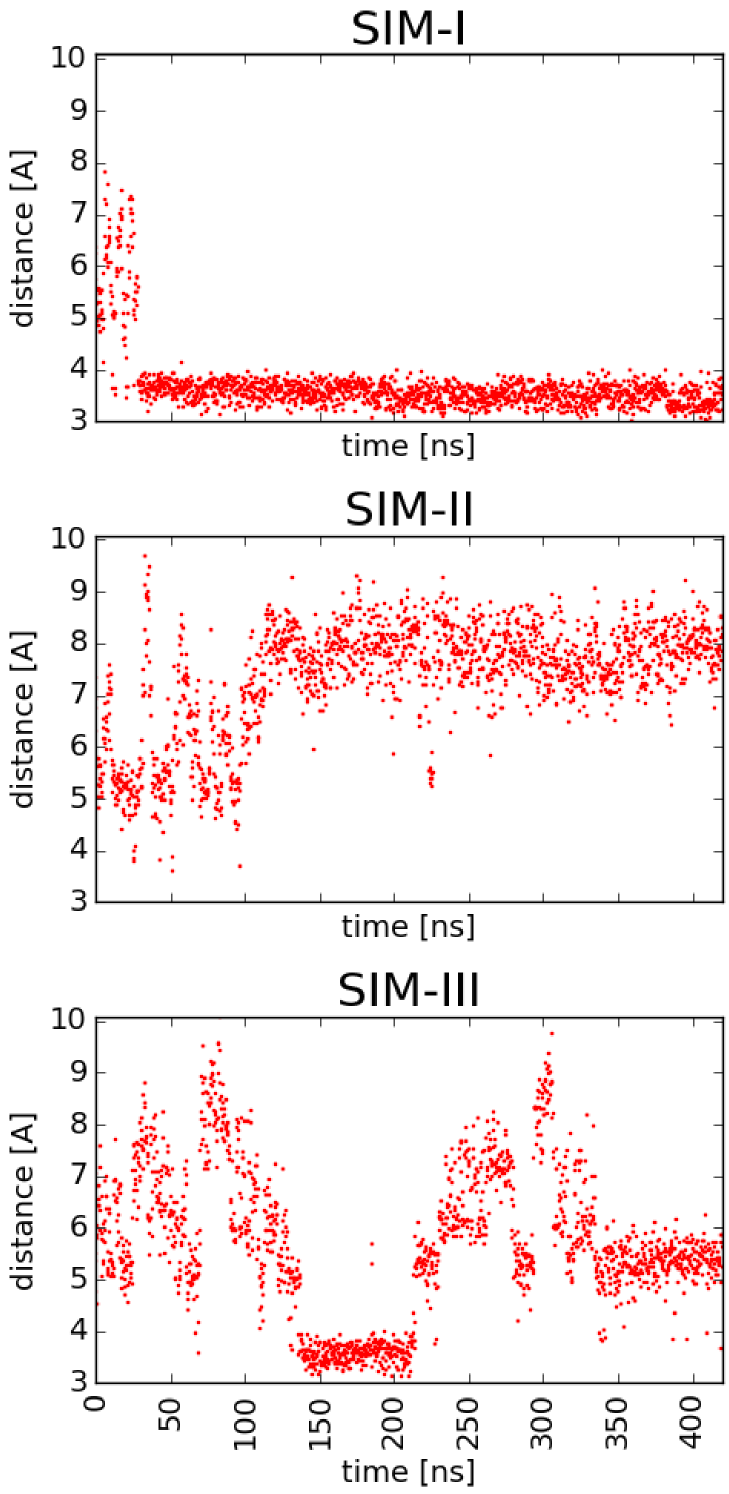

Figure SM-SIM-2. Time evolution of distance between Oγ atom of Ser346 and C of Arg residue in compound 3. Data come from simulations SIM-I, SIM-II, SIM-III.

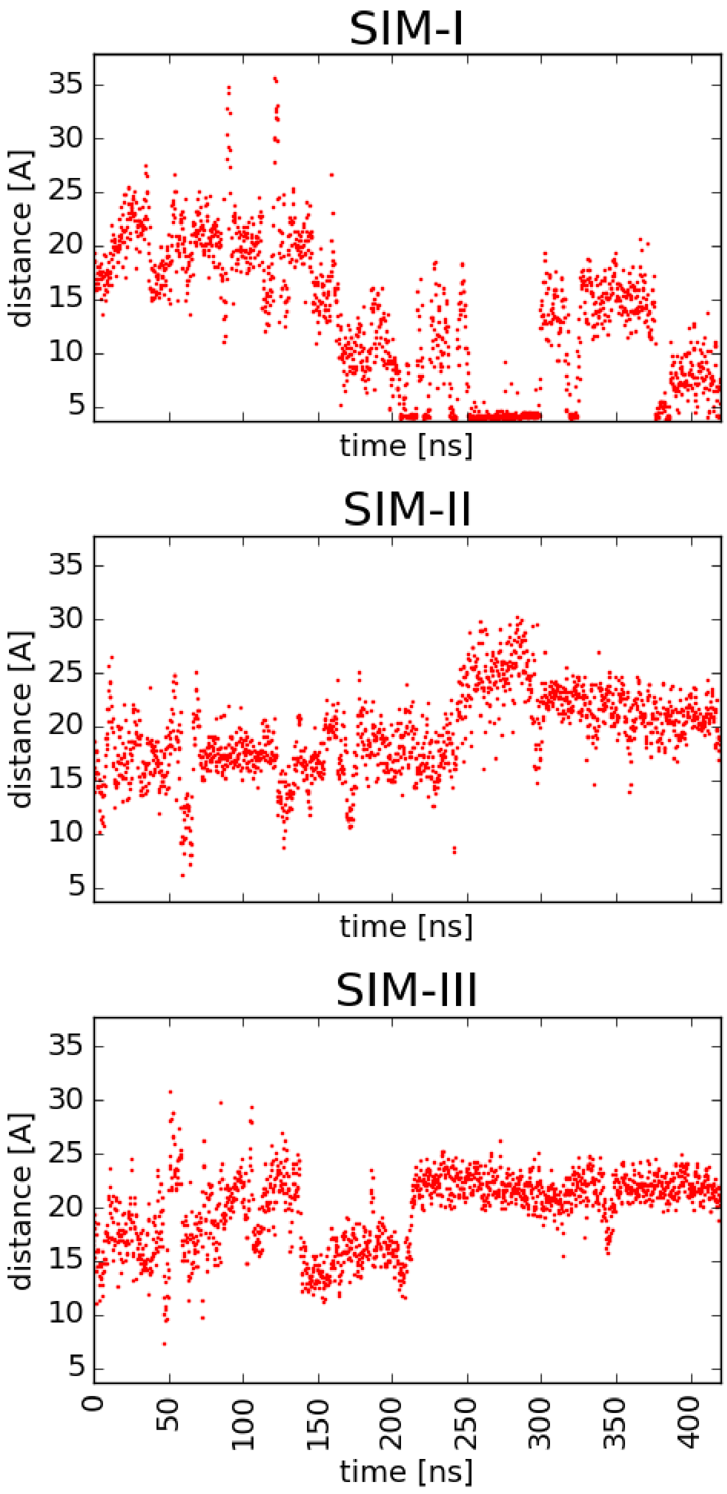

Figure SM-SIM-3. Time evolution of distance between Cδ atom of Glu319 and Cζ of Har residue in compound 3. Data come from simulations SIM-I, SIM-II, SIM-III.

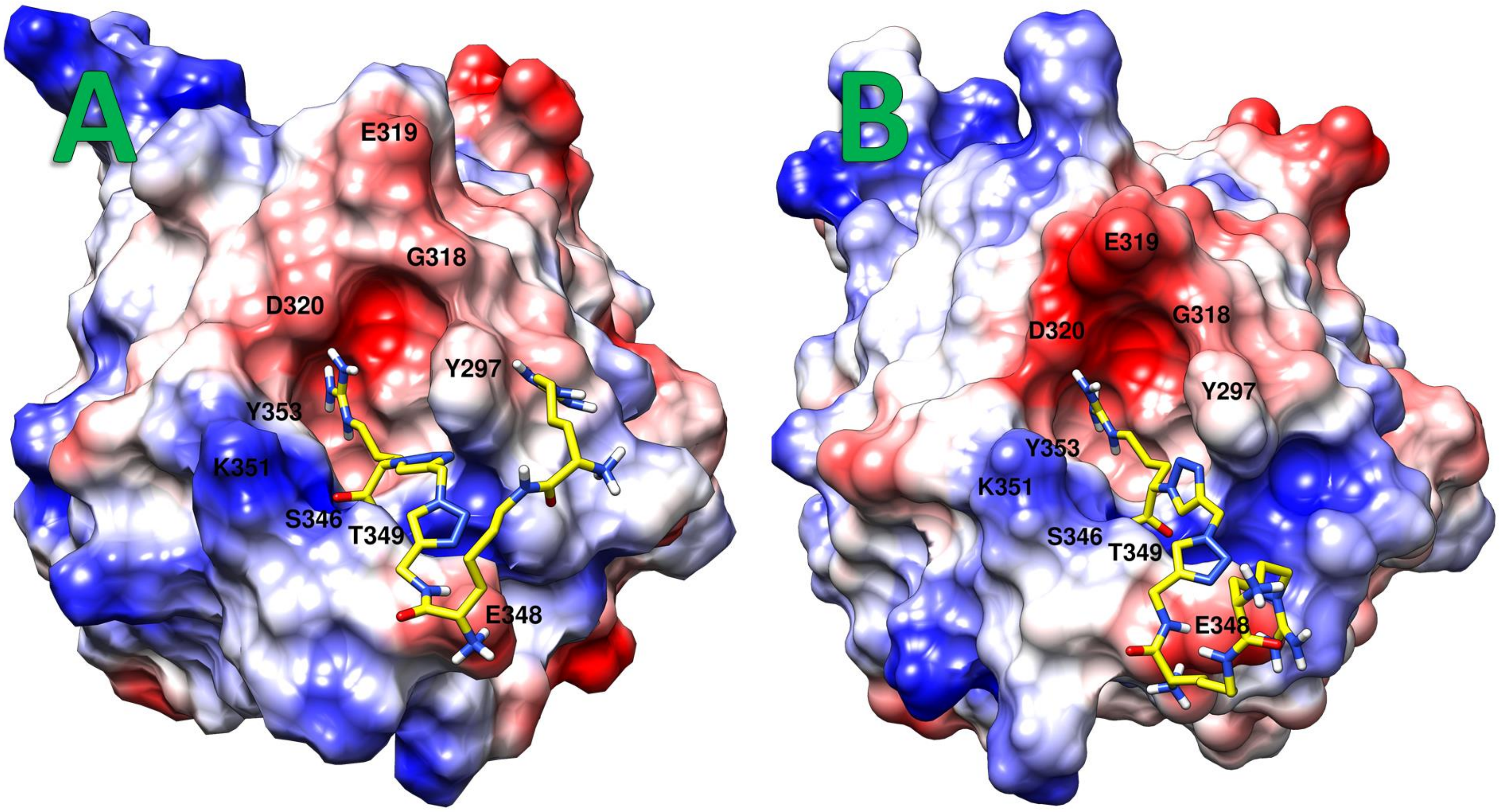

Figure SM-SIM-4. Representative snapshots out of the trajectories presented according to the convention given in the main paper. **A.** SIM-I, t = 50.0 ns **B.** SIM-I, t = 100.0 ns. *continued*

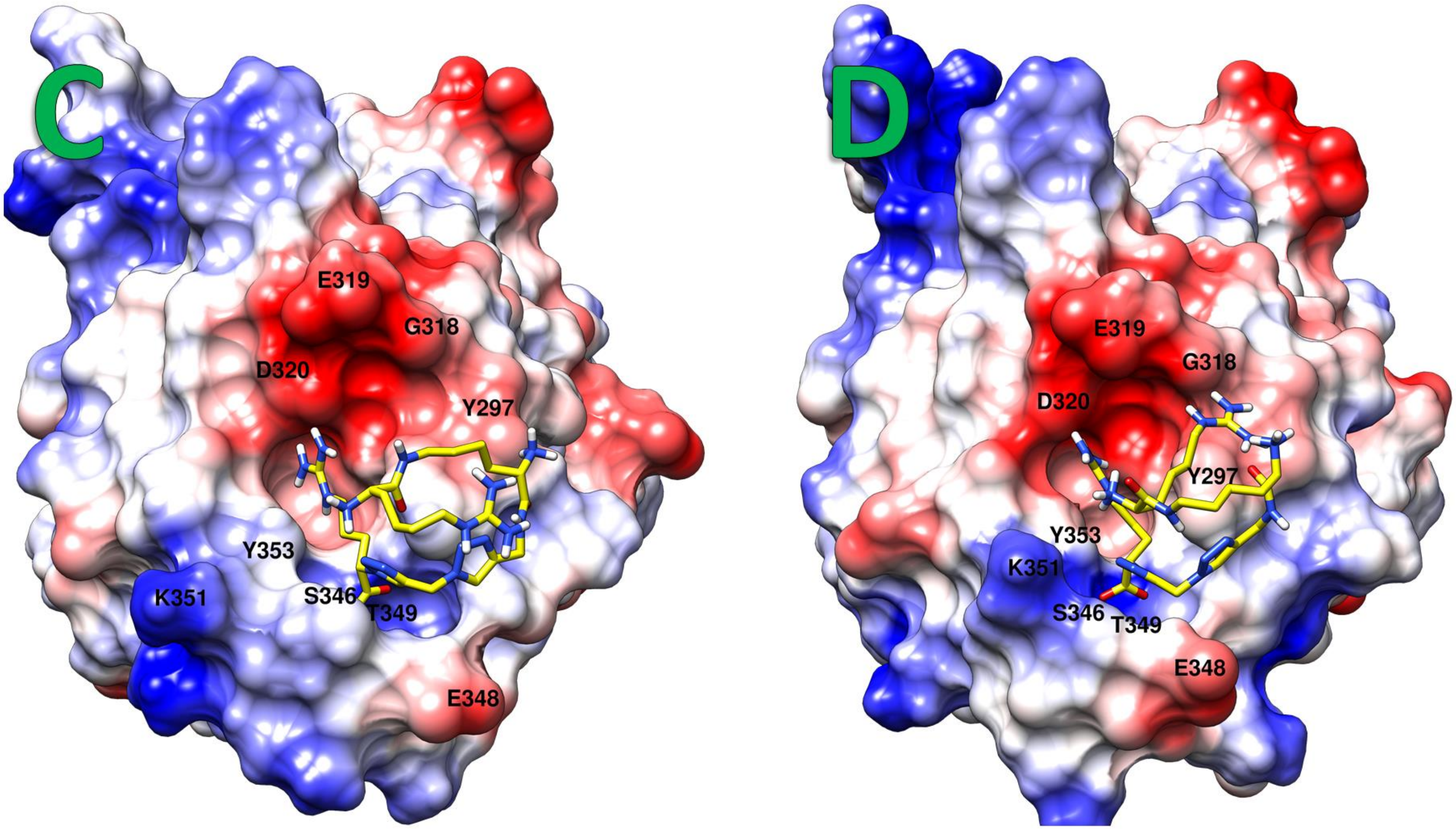

Figure SM-SIM-4. *Continuation* Representative snapshots out of the trajectories presented according to the convention given in the main paper. **C.** SIM-I, t = 150.0 ns **D.** SIM-I, t = 350.0 ns. *continued*

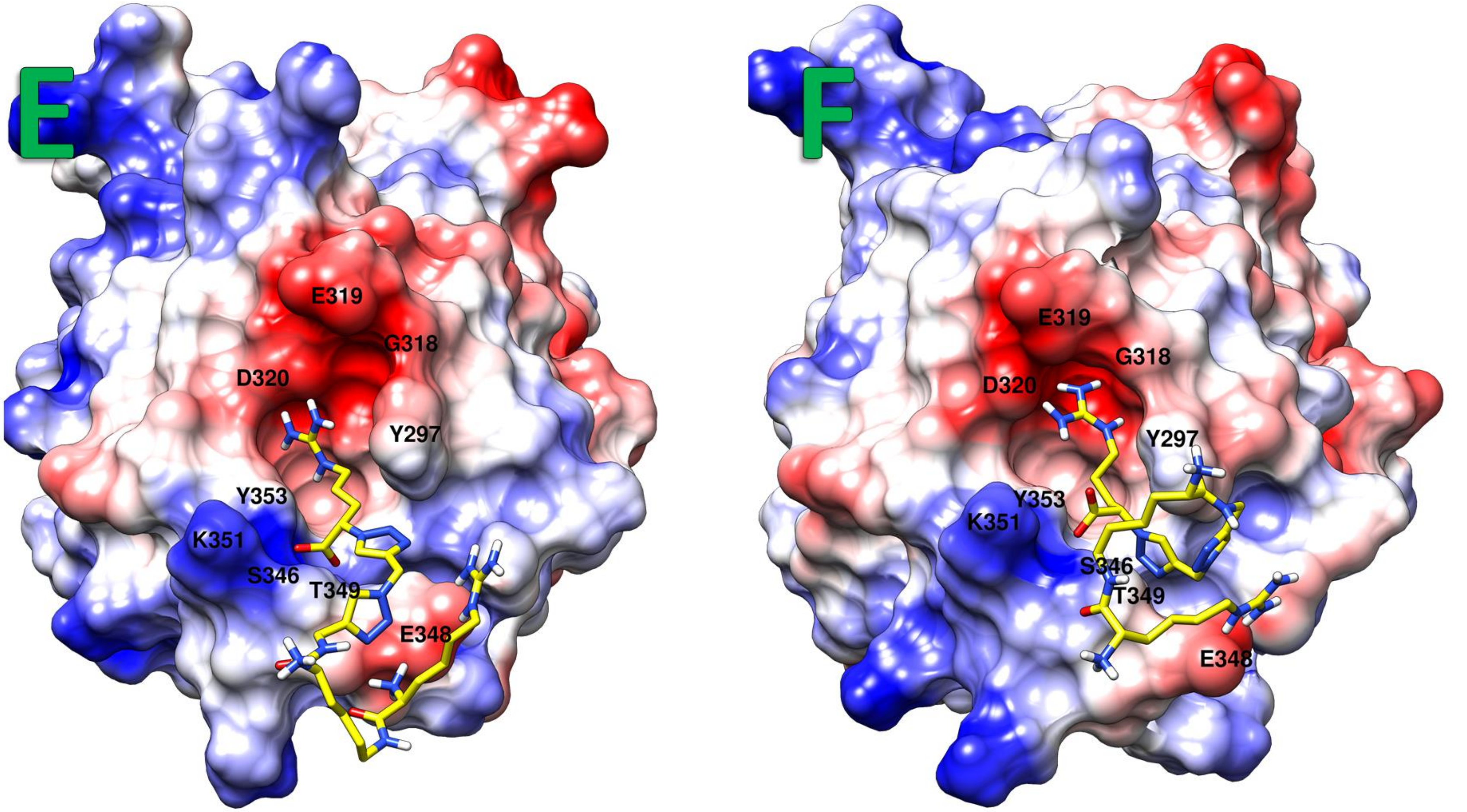

Figure SM-SIM-4. *Continuation* Representative snapshots out of the trajectories presented according to the convention given in the main paper. **E.** SIM-I, t = 400.0 ns **F.** SIM-II, t = 100.0 ns. *continued*

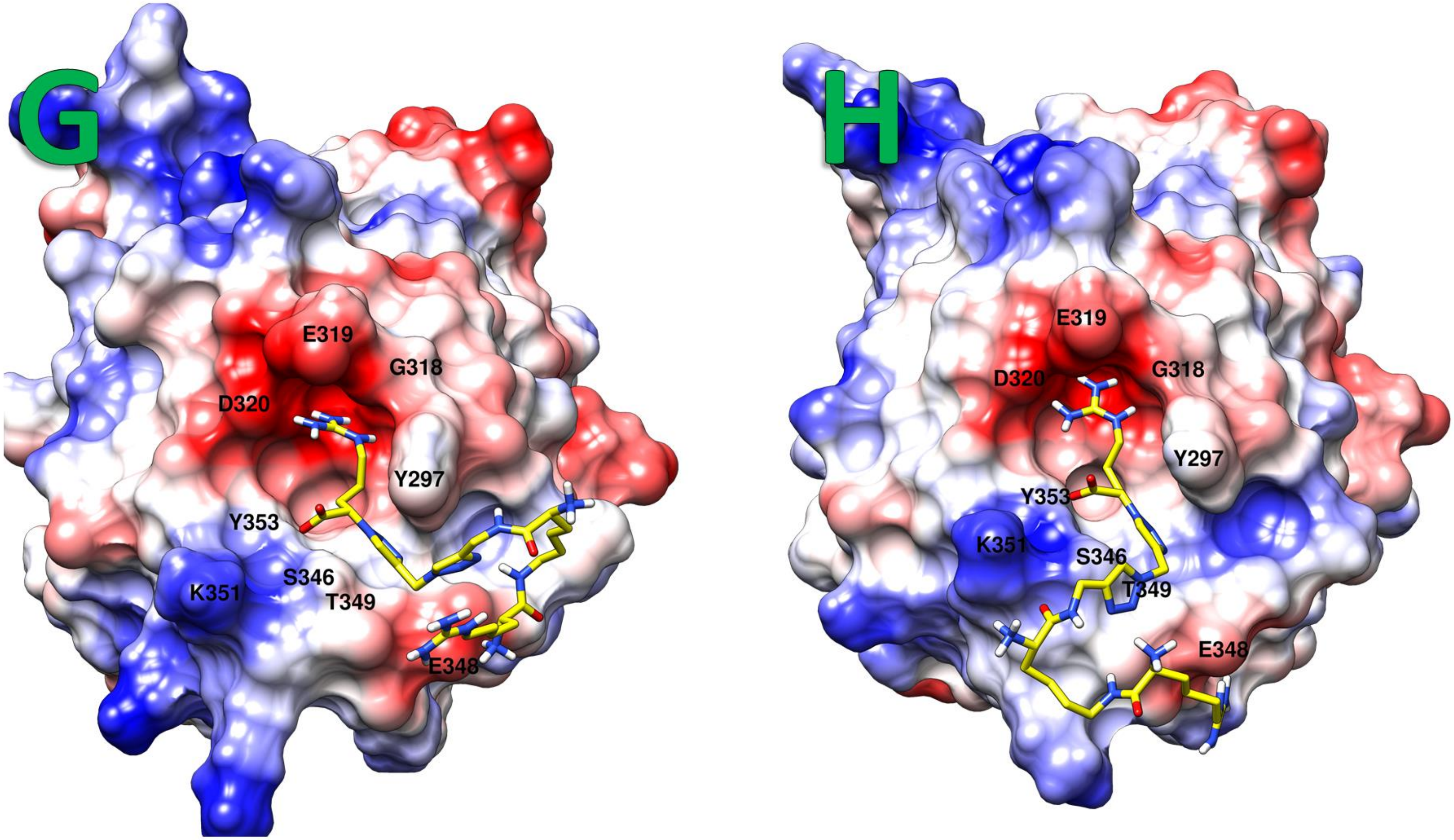

Figure SM-SIM-4. *Continuation* Representative snapshots out of the trajectories presented according to the convention given in the main paper. **G.** SIM-II, t = 200.0 ns **H.** SIM-II, t = 350.0 ns. *continued*

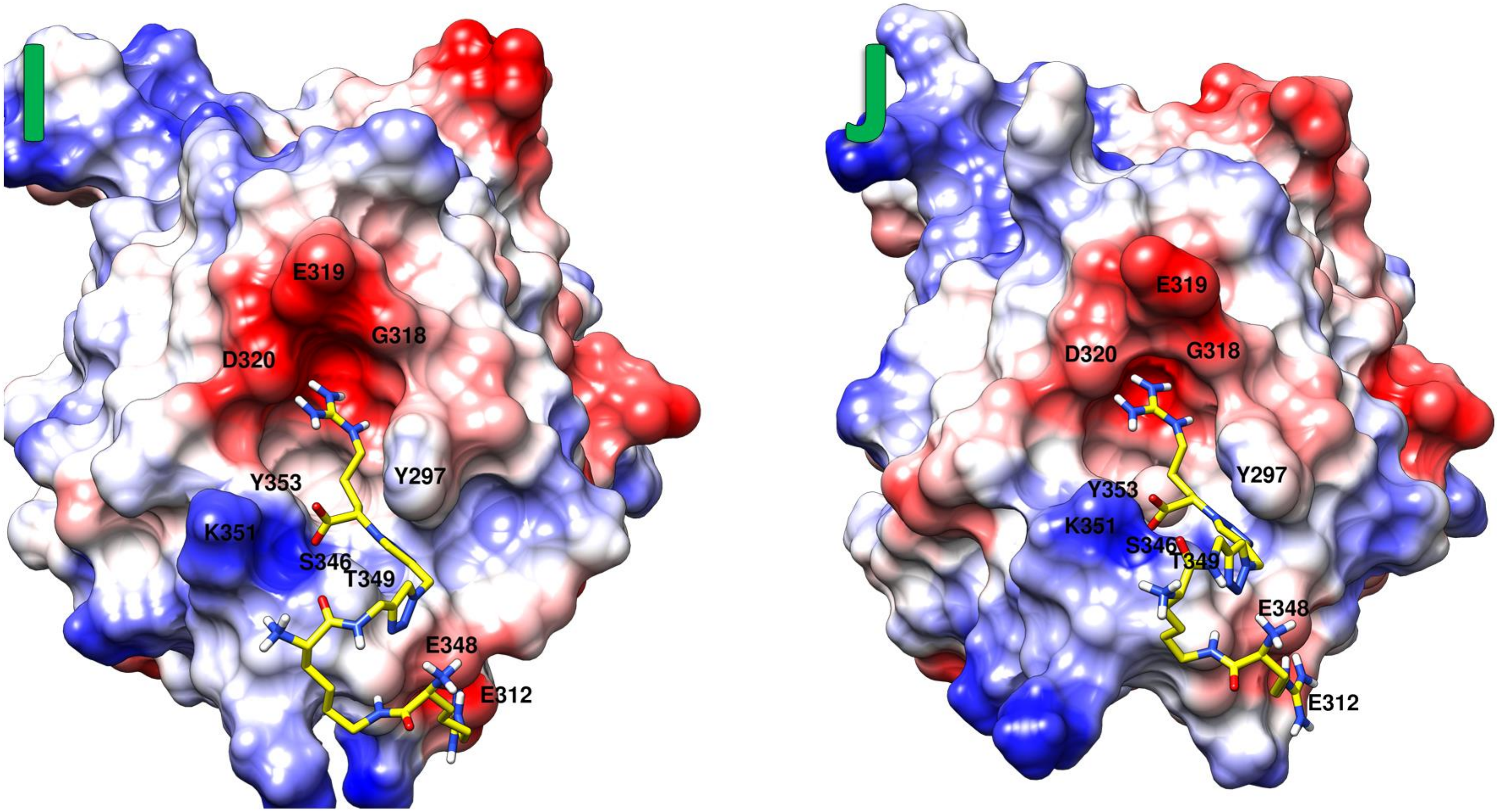

Figure SM-SIM-4. *Continuation* Representative snapshots out of the trajectories presented according to the convention given in the main paper. **I.** SIM-III, t = 250.0 ns **J.** SIM-III, t = 400.0 ns.

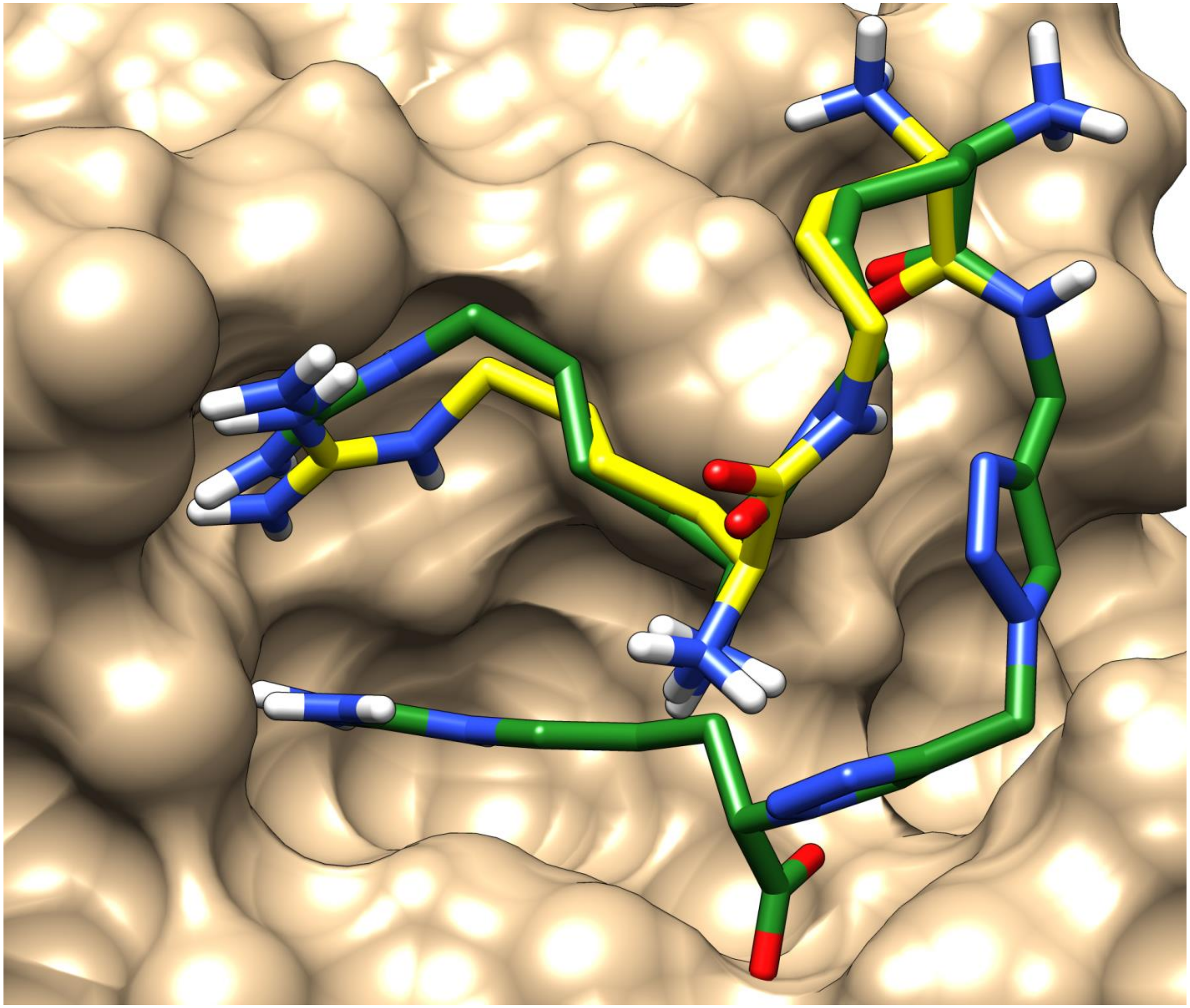

Figure SM-SIM-5. Complex of compound **3** (yellow) with NRP-1 as found in SIM-I, a representative snapshot of the binding mode discussed in the main text. In forest green given is a copy of molecule 3 but with inverted stereochemistry at Cα of Lys residue and optimized geometry of the Lys(Har) fragment (restraints of protein atoms and the remaining part of compound **3**). As it can be seen, the inversion of stereochemistry does not require major readjustment of the Lys(Har) fragment and allows for retaining of the key contacts.

Table SM-SIM-1. Persistency of the interactions between compound **3** and Neuropilin-1 as found in molecular dynamics simulations.

	**SIM-I**	**SIM-IA**	**SIM-IB**	**SIM-IC**	**SIM-II**	**SIM-III**
C-terminal Arg guanidine with Asp320	99%	100%	100 %	100%	100%	100%
C-terminal Arg carboxylate with Ser346	93%	100%	100%	100%	7%	26%
C-terminal Arg carboxylate with Thr349	96%	75%	98%	99%	20%	67%
Har guanidine with Glu319	19%	27%	50%	42%	0%	0%
hydrogen of the amide bond joining Har and Lys with the phenolic function of Tyr297	28%	30%	48%	45%	8%	2%
2Trl$_{C-N}$ (C5H) with Glu348	24%	16%	36%	47%	1%	0%

SM-RES. Proteolytic resistance

Compound 3

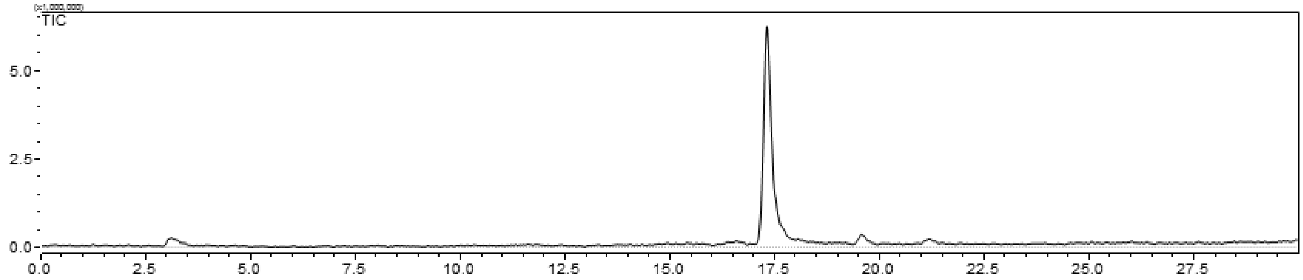

Figure SM-STA-1. TIC chromatogram for the blank compound **3**.

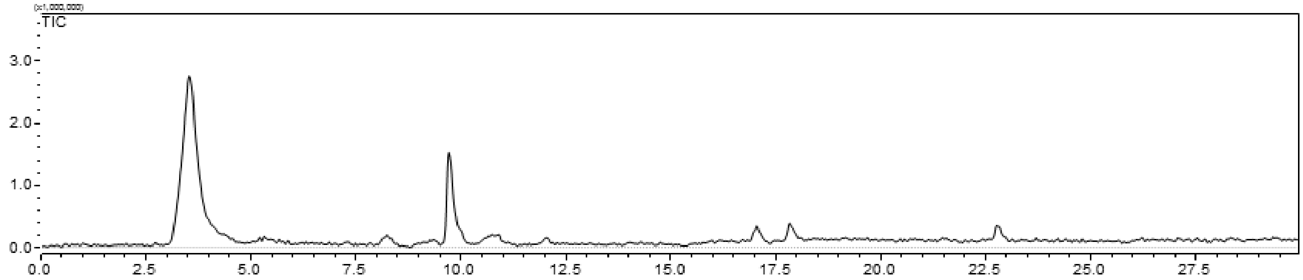

Figure SM-STA-2. TIC chromatogram for the blank human plasma.

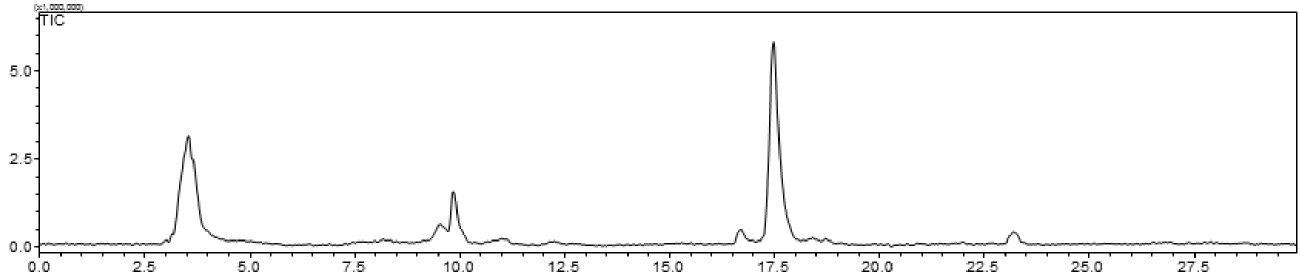

Figure SM-STA-3. TIC chromatogram for the compound **3** at starting point (0 h).

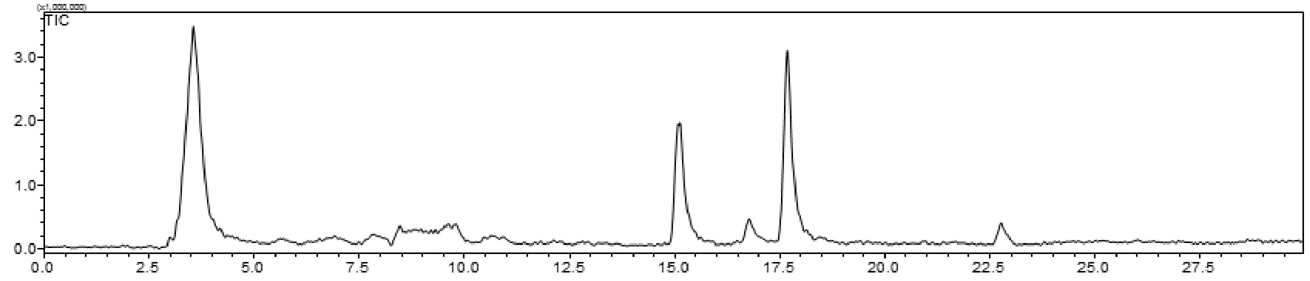

Figure SM-STA-4. TIC chromatogram for the compound **3** at ending point (48 h).

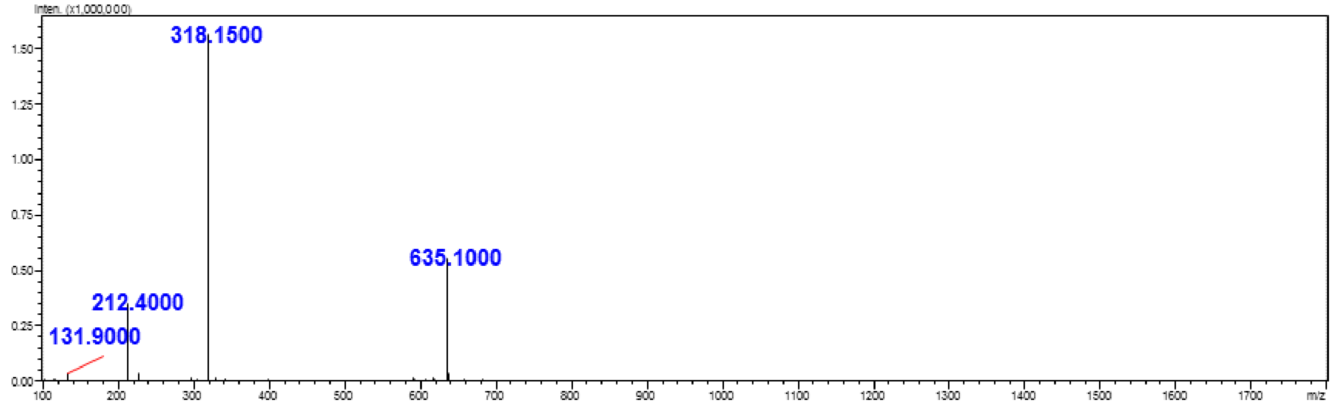

Figure SM-STA-5. Mass spectrum for the compound **3** (RT c.a. 17.5 min).

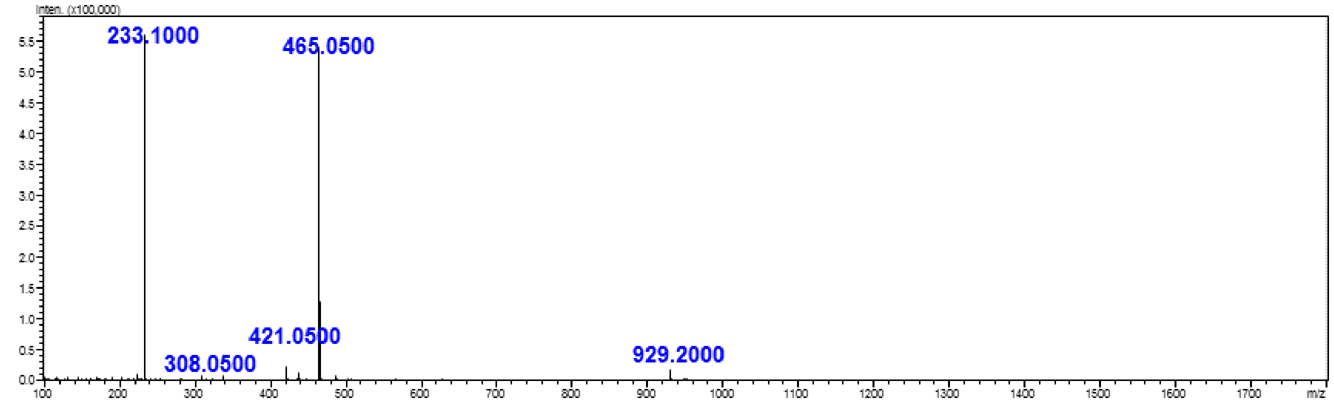

Figure SM-STA-6. Mass spectrum for metabolite (RT c.a. 15 min).

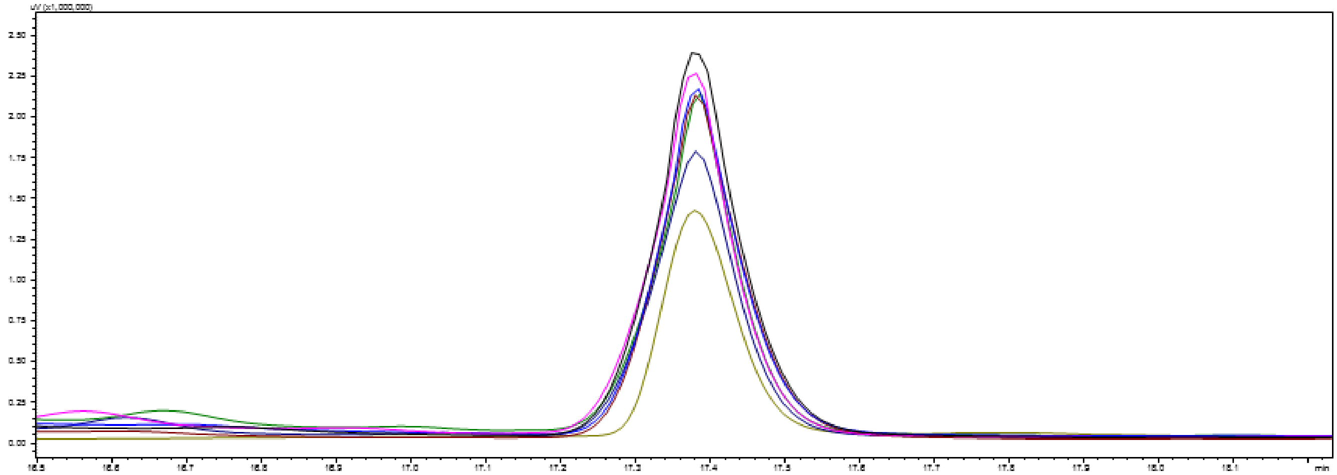

Figure SM-STA-7. Overlap of chromatograms at 210 nm for the compound **3** at all timepoints.

Table SM-STA-1. Data sheet for figure 8 in main text taken from peak integration for compound **3**.

time [h]	Area 1st repet.	Area 2nd repet.	Area 3rd repet.	Area average (AV)	Standard deviation (SD)	AV/SD	Relative
0	18215057	18610127	18966987	18597390	307106	1.7%	100.0%
8		17025853	16948717	16987285	38568	0.2%	91.3%
16		16487412	16990000	16738706	251294	1.5%	90.0%
24	15387650	16649994	16850114	16295919	647419	4.0%	87.6%
32		15197203	15216682	15206943	9740	0.1%	81.8%
40	13337943	13648096	13983715	13656585	263704	1.9%	73.4%
48	12668865	14197196		13433031	764166	5.7%	72.2%

Compound 4

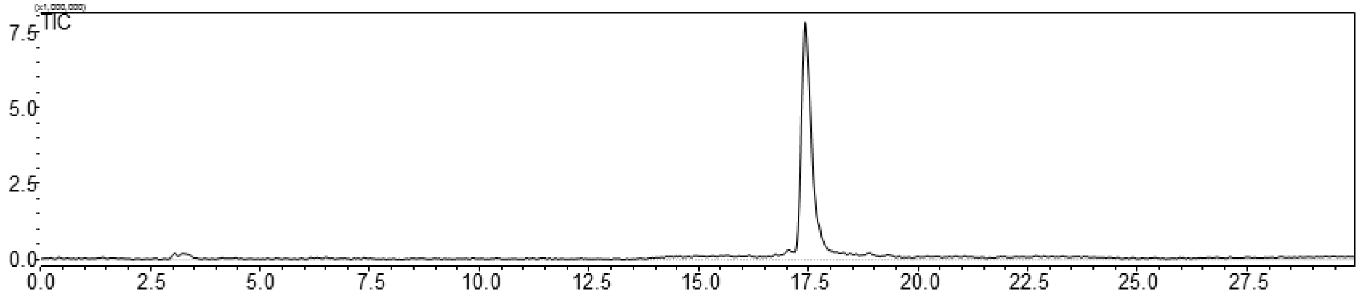

Figure SM-STA-8. TIC chromatogram for the blank compound **4**.

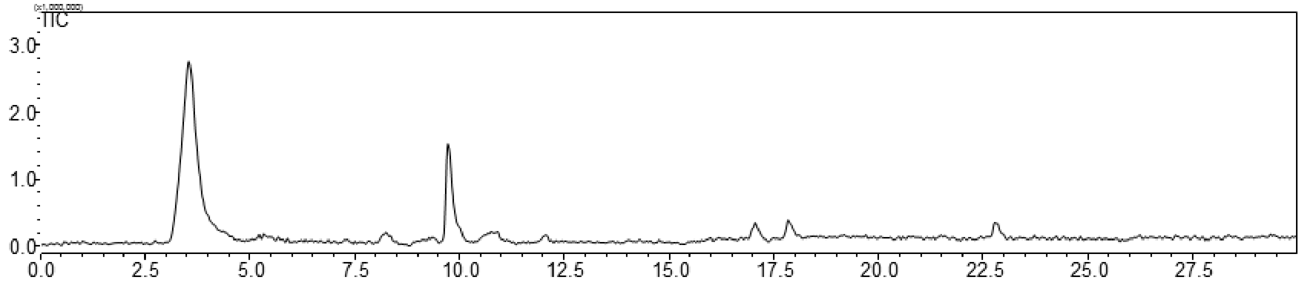

Figure SM-STA-9. TIC chromatogram for the blank human plasma.

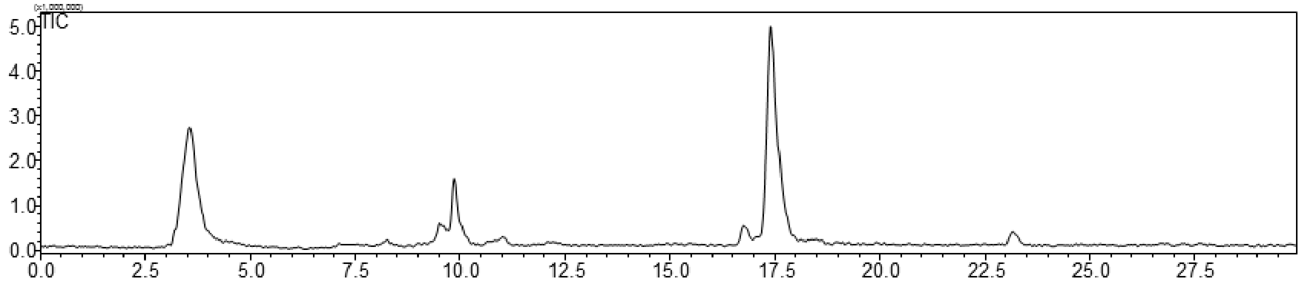

Figure SM-STA-10. TIC chromatogram for the compound **4** at starting point (0 h).

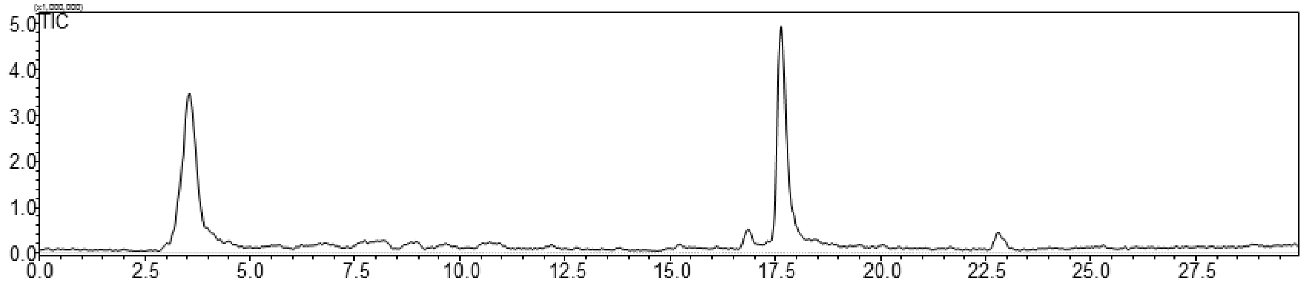

Figure SM-STA-11. TIC chromatogram for the compound **4** at ending point (48 h).

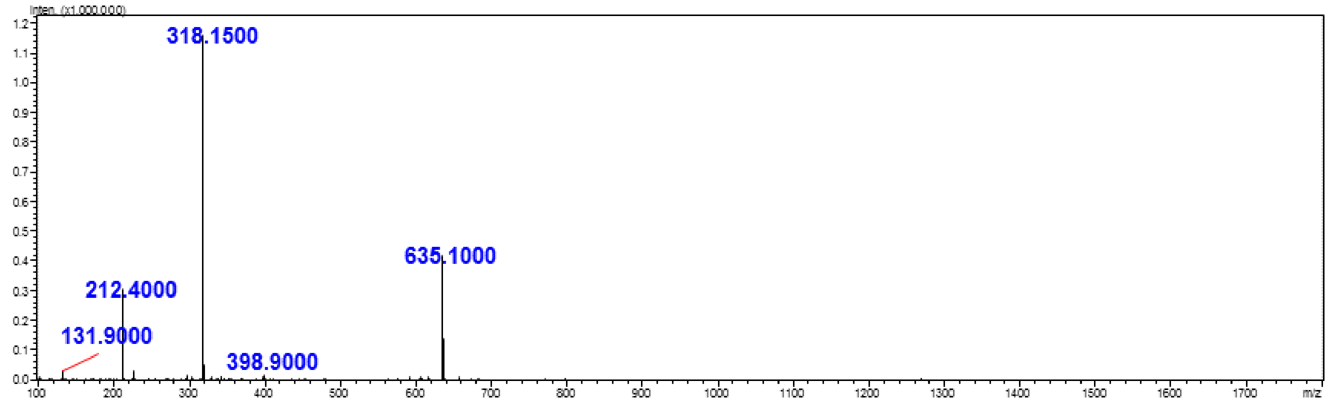

Figure SM-STA-12. Mass spectrum for the compound **4** (RT c.a. 17.5 min).

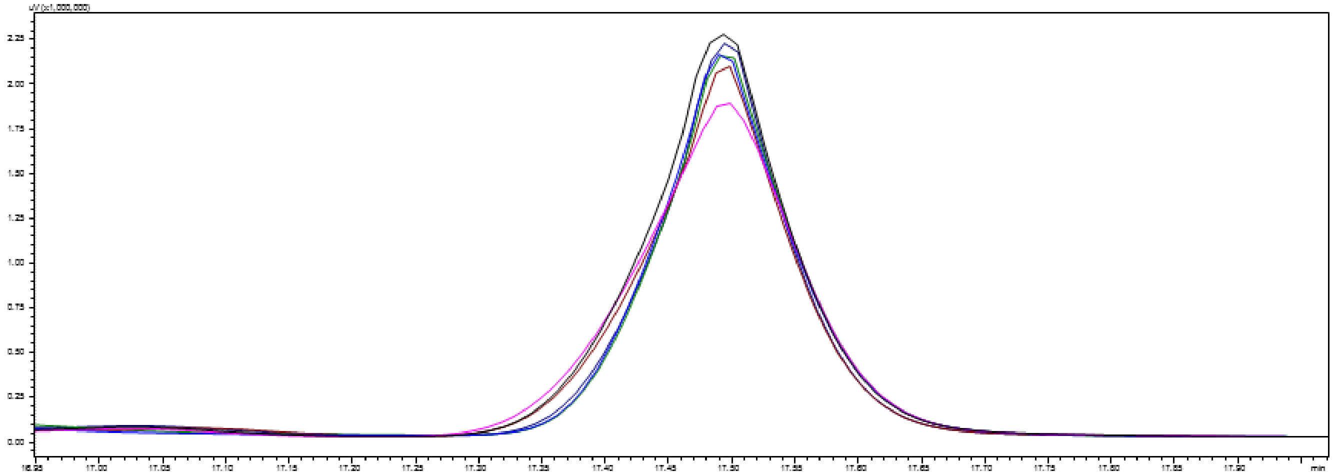

Figure SM-STA-13. Overlap of chromatograms at 210 nm for the compound **4** at all timepoints.

Table SM-STA-2. Data sheet for figure 8. in main text taken from peak integration for compound **4**.

time [h]	Area 1st repet.	Area 2nd repet.	Area 3rd repet.	Area average (AV)	Standard deviation (SD)	AV/SD	Relative
0	17745877	17829358	17962532	17845922	89221	0.5%	100.0%
8	17199190	17944581	18094145	17745972	391425	2.2%	99.4%
16	16969384	17225800	17049665	17081616	107092	0.6%	95.7%
24	16265501	17031240	out	16648371	382870	2.3%	93.3%
32	16380385	16589325	16886092	16618601	207489	1.2%	93.1%
40	16189564	16614762	17010034	16604787	335030	2.0%	93.0%
48	16223374	16328206	16609676	16387085	163110	1.0%	91.8%

SM-SUR. Preliminary cell survival test

Compound 4

Table SM-CYT-1. Data sheet for figure 9. in main text accessed by Muse Cell Analyser (all values in %).

	**24h**	**48h**	**24h**	**48h**	**24h**	**48h**
**Concentration [µM]**	**1st repetition**		**2nd repetition**		**3rd repetition**	
**k 1**	99.10	99.60	99.60	99.95	99.96	out
**k 2**	99.00	99.30	98.80	99.96	99.95	99.50
**k 3**	98.90	99.70	99.70	out	100.00	98.70
**0.1**	99.40	99.40	99.80	99.97	99.98	98.80
**0.1**	99.60	99.50	99.60	99.99	99.99	99.30
**0.1**	98.90	99.70	99.70	99.96	99.99	99.70
**1**	99.30	99.60	99.70	99.97	100.00	99.40
**1**	98.90	99.40	99.50	99.70	99.99	99.60
**1**	99.00	98.90	99.40	99.83	out	98.60
**10**	99.00	99.40	99.90	99.96	99.96	98.80
**10**	99.00	99.50	99.50	99.94	99.94	99.20
**10**	99.10	99.50	99.50	99.96	99.96	99.70
**50**	98.70	99.70	99.90	99.95	99.98	98.80
**50**	98.80	99.70	98.80	99.96	99.98	99.70
**50**	98.40	99.30	99.30	99.97	99.98	99.00
**100**	98.60	99.20	99.40	99.96	99.98	99.10
**100**	98.90	99.50	99.70	99.95	100.00	99.40
**100**	99.60	99.50	99.60	99.97	99.97	99.30
